# Colon cancer subtypes: concordance, effect on survival and selection of the most representative preclinical models

**DOI:** 10.1038/srep37169

**Published:** 2016-11-16

**Authors:** Zsófia Sztupinszki, Balázs Győrffy

**Affiliations:** 1 MTA TTK Lendület Cancer Biomarker Research Group, 1117, Budapest, Hungary; 2 2^nd^ Dept. of Pediatrics, Semmelweis University, 1094, Budapest, Hungary

## Abstract

Multiple gene-expression-based subtypes have been proposed for the molecular subdivision of colon cancer in the last decade. We aimed to cross-validate these classifiers to explore their concordance and their power to predict survival. A gene-chip-based database comprising 2,166 samples from 12 independent datasets was set up. A total of 22 different molecular subtypes were re-trained including the CCHS, CIN25, CMS, ColoGuideEx, ColoGuidePro, CRCassigner, MDA114, Meta163, ODXcolon, Oncodefender, TCA19, and V7RHS classifiers as well as subtypes established by Budinska, Chang, DeSousa, Marisa, Merlos, Popovici, Schetter, Yuen, and Watanabe (first authors). Correlation with survival was assessed by Cox proportional hazards regression for each classifier using relapse-free survival data. The highest efficacy at predicting survival in stage 2–3 patients was achieved by Yuen (p = 3.9e-05, HR = 2.9), Marisa (p = 2.6e-05, HR = 2.6) and Chang (p = 9e-09, HR = 2.35). Finally, 61 colon cancer cell lines from four independent studies were assigned to the closest molecular subtype.

Colorectal cancer (CRC) is the fourth leading cause of cancer-related death. Chemotherapy and targeted therapy double the survival for patients with advanced disease up to 30 months^1^. The current set of agents includes the cytotoxic agents 5-fluorouracil, capecitabine, irinotecan, and oxaliplatin; the angiogenesis inhibitors bevacizumab and aflibercept; the anti-EGFR antibodies cetuximab and panitumumab; and the multi-kinase inhibitor regorafenib. In addition, a multitude of additional agents are in development so that soon it will be possible to target almost any genetic alteration.

However, colorectal cancer is not a single disease but rather a set of molecularly distinctive diseases having the same clinical presentation^2^. Thus, to achieve our goal of personalized therapy, first we have to stratify patients into clinically relevant molecular subtypes. In principle, classification using single genes or gene signatures is possible. The most commonly mutated pathways in colorectal cancer include APC (80% of patients)^3^, the mutually exclusive RAS (hit in 43% of patients)^4^ and BRAF (15%)^5^ pathways and the Wnt pathway (93%)^6^. Of these, two isoforms of RAS, KRAS and NRAS, as well as BRAF are clinically used to select patients not eligible for anti-EGFR therapy. Markers that have been clinically introduced in colon cancer include RAS mutations that exclude anti-EGFR therapy and microsatellite instability (MSI) as a marker of good prognosis in stage II colon cancer.

On the other hand, a plethora of multigene signatures utilizing gene expression profiles obtained from tumour samples have been published in the last decade. These classify patients into molecular subtypes ranging from two up to six. Most of these classifiers focus on risk prediction – similarly to other oncologic disorders such as breast cancer (reviewed in ref. 7). In principle, this approach is therapeutically useful assuming that patients having a high risk of relapse will also benefit the most from chemotherapy.

However, there are multiple unsolved issues with the proposed subtypes. Although the performance of many of these classification schemes has been assessed using an independent set of patients, to date no study has compared them using the same set of patients. Secondly, the ability to select a clinically relevant subtype using patient-derived material does not ensure the immediate translation of these results into patient therapy. To this end, agents specific for the given subdivision have to be developed and tested in an appropriate model system. However, essentially none of the studies to date have provided guide for this initial step of any preclinical trial.

In this study, our goal was to evaluate published molecular subtypes using the same large set of patients. In addition to comparing the molecular subtypes to each other, we also ranked them according to their capability regarding prediction of survival. Moreover, we obtained gene expression signatures of colorectal cancer cell lines and evaluated each cell line to identify the most representative preclinical model for each subtype within each classifier.

## Results

### Validation datasets and clinical characteristics

**Figure 1:**
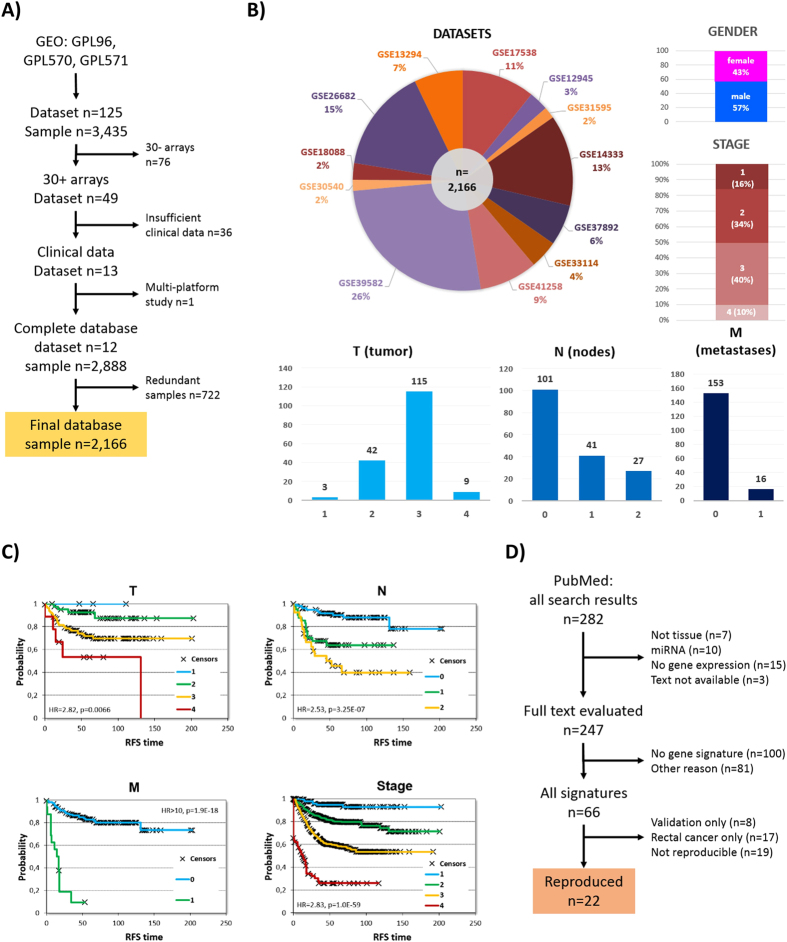
Summary of pooled database setup and classifier selection. A flowchart depicting dataset identification starting with the combination of “colon cancer” and the three different platforms in GEO (**A**). Composition of the entire database – the 12 datasets included and basic clinical characteristics – *sample numbers are given for TNM because these data were available for only a fraction of the patients* (**B**). Correlation between survival, TNM and stage in the entire database (**C**). Identification of classifiers through a PubMed search (**D**).

A summary of the database construction starting from a GEO search is presented in Fig. 1A. The complete database is based on following GEO datasets: GSE17538, GSE12945, GSE31595, GSE14333, GSE37892, GSE33114, GSE41258, GSE39582, GSE30540, GSE18088, GSE26682, and GSE13294. The largest dataset (GSE39582) with 566 samples accounted for 26% of the entire database. For all patients, 57.4% were male and 74.2% were either stage II or III. The median relapse-free survival was 43.12 months and the median age 69 years. Aggregate clinical parameters for the entire database utilized in the validation analysis are depicted in Fig. 1B, and the clinical properties for each of the datasets including gender, grade, MSI, stage, age and location are listed in Supplementary Table 1.

### Classification algorithms and most-central genes

After screening the abstracts of 282 publications and reading the full text of 247 papers, we narrowed down the list of classifiers to n = 41. Either by employing the published supplementary material or by utilizing the original training datasets we were able to re-programme 22 of these. A flowchart of candidate classifier identification is portrayed in Fig. 1D. A complete list of all 22 classifiers implemented including patient eligibility, details of the classification algorithm and the parameters of the training and validation sets utilized in the original publication are shown in Table 1.

To compare the composition of the classifiers and to assess the power of individual genes, we extracted the list of genes included in each of the classifiers (see Supplementary Table 2). Then, the genes were ranked after computing a gene score for each of the markers. The three highest scoring genes each included in four different classifiers were CTGF, GADD45B and FAP. When evaluating the correlation between these genes and survival in univariate analysis in the entire database using the median expression as a cutoff, each of them delivered high significance [CTGF: p = 2.7E-07, HR = 1.8 (1.4–2.3); GADD45B: p = 6.7E-05, HR = 1.6 (1.3–2.0) and FAP: p = 4.6E-06, HR = 1.7 (1.3–2.1)].

### Concordance in classification output

In the next step, we assigned each of the patients to one of the subtypes in each classifier. Supplementary Table 3 contains the designation for each sample.

**Figure 2:**
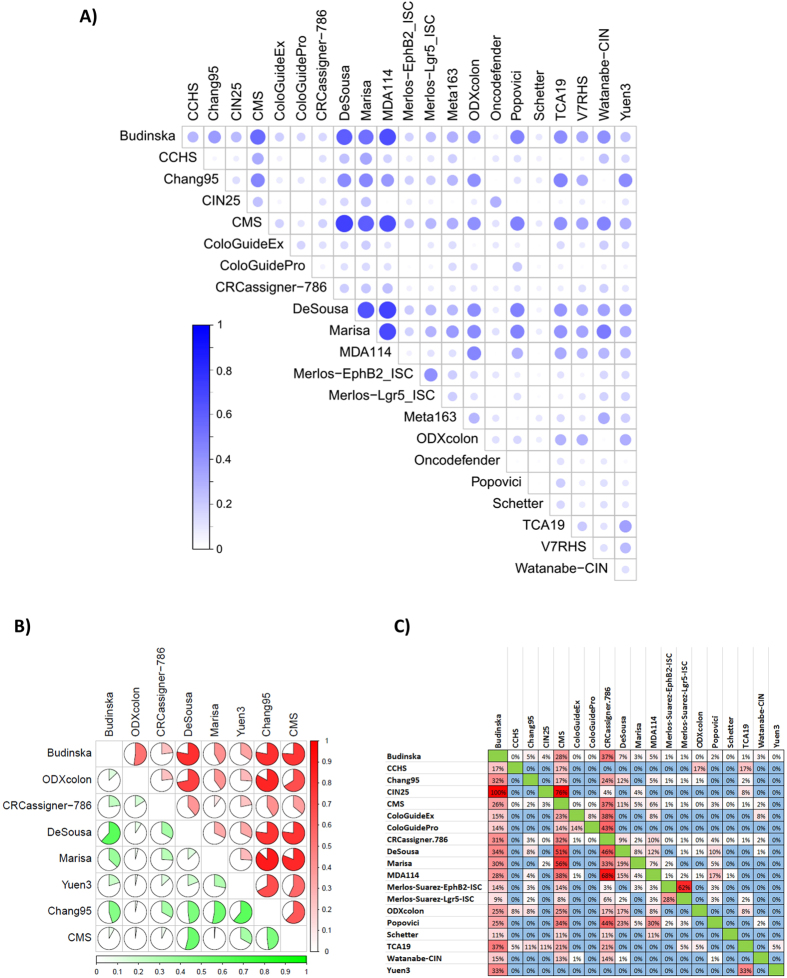
Concordance of classifier output and gene composition. Concordance across all classifiers in all samples (**A**) and for those identifying a sample as having a bad (*red*) or good (*green*) prognosis for the top eight classifiers in stage II/III patients (**B**). In each case, a Cramer’s V of 1 represents perfect concordance and 0 equals complete discordance. Percentile overlap of the list of genes included in the classifiers (**C**). *An example for interpretation: Budinska contains 100% of the genes in the CIN25 signature*, *but these account for only 4% of all Budinska genes. V4HRS*, *Meta163 and Oncodefender are not included as these had less than 1% overlap against any other classifier*.

Because a general basis for the classification is the division of the samples into good-prognosis and bad-prognosis groups, it is possible to evaluate the concordance of the classification output across multiple classifiers. In this analysis, we observed the highest overlap between MDA114 and DeSousa (Cramer’s V = 0.711). Similarly high concordance was observed between Marisa and MDA114 (0.681) outputs, and between the Budinska and both the DeSousa (0.601) and MDA114 (0.665) outputs. Concordance of classification output for all tests is depicted in Fig. 2A. Cramer’s V values for each pair are listed in Supplementary Table 4.

A more exhaustive evaluation is possible if the assignment of a sample into a good or a bad prognosis cohort is assessed separately. This approach is particularly important because one generally does not have the opportunity to perform each of the classifiers separately, and thus high concordance for results among particular assays will enable more reliable interpretation. Because such a correlation is most relevant for tests with high actual predictive potential, we performed a detailed analysis for the eight best-performing tests. Concordant assignment to a cohort with bad prognosis was most probable when applying the Marisa and Chang95, ODXcolon and Chang95 and DeSousa and Marisa tests. Assignment to a cohort with good prognosis was most likely similar for DeSousa and Chang95 and for DeSousa and ColoGuideEx results. Detailed results for identifying a sample as having a good or bad prognosis are displayed in Fig. 2B.

In addition to classification concordance, the proportional overlap among the genes comprising the tests was also compared. The highest overlap was observed between the CIN25 and Budinska (100% - *all genes of the CIN25 are included in Budinska*), CIN25 and CMS (76%) and MDA114 and CRCassigner.786 (68%) tests. The results for all tests are listed in Fig. 2C.

### Power to predict survival is different for each molecular classifier

When investigating the correlation between clinical and pathological parameters and survival, the most robust significance was reached when comparing stage I and stage III patients (n = 619, HR = 9.09, p = 3.4E-12). (Significance was higher when comparing stage I and stage IV, but with a low sample number.) MSI was also significant (n = 618, HR = 2.78, p = 1.3E-04). Grade (p = 0.62), gender (p = 0.12) and location (p = 0.52) were not significantly correlated with relapse-free survival. When assessing the effect of T, N and M, the highest difference was found between samples with metastasis (M = “1”) compared to non-metastatic patients (n = 169, p < 1.9E-18, HR > 10). Survival plots for stage and TNM are displayed in Fig. 1C.

**Figure 3:**
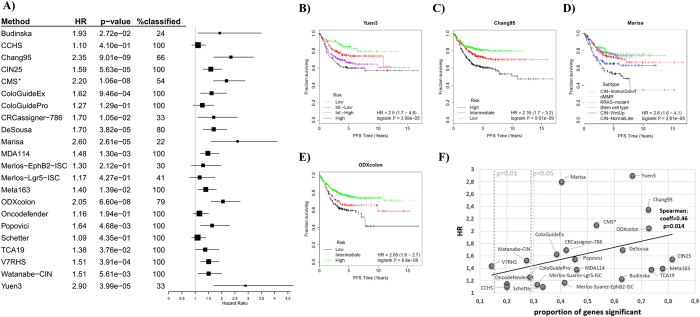
Relative classifier performance in stage II/III patients. A forest plot showing the performance of all classifiers in stage II and III patients (**A**). Samples included in the original training sets were excluded from the validation analysis (*with the exception of CMS). For classifiers with more than two outputs, the best- and worst-performing cohorts were compared when computing the hazard rate. Kaplan-Meier plot for the best-performing classifiers including Yuen3 (**B**), Chang95 (**C**), Marisa (**D**) and ODXcolon (**E**). A higher percentage of genes included in the classifier significant in univariate analysis results in a higher hazard rate achieved by the classifier (*all patients for CMS) (**F**). The dotted lines represent the proportion of genes significant at p = 0.05 and p = 0.01 among all genes on the gene chips in univariate analysis.

Survival analysis was first performed by using stage II and III patients only because prediction in this cohort has the highest clinical relevance and because over 74% of all patients in the validation database fall into this category. As computation of the hazard rate can only include two cohorts, we selected the best- and worst-performing subtypes for each classifier. Thus, intermediate cohorts and samples not classified were excluded. A forest plot listing all the classifiers is presented in Fig. 3A, and all the survival plots for each of the 22 classifiers are displayed in Supplementary Figure 1. The best-performing classifiers were the Yuen3 (p = 3.9E-05, HR = 2.9), Marisa (p = 1.74E-05, HR = 2.8), Chang95 (p = 9E-09, HR = 2.35), ODXcolon (p = 6.6E-02, HR = 2.05) and DeSousa (p = 3.8E-05, HR = 1.7) (see Fig. 3B–E). Similar results were obtained when using all patients (forest plot in Supplementary Figure 2 for all patients, and in Supplementary Figures 3 and 4 for stage II and stage III patients separately. The individual KM-plots are presented in Supplementary Figure 5).

When comparing the prediction efficacy of the classifiers to MSI status, gender, MKI67 expression and CDX2 expression, only the CRCassigner.786, the Meta163, Watanabe-CIN, Marisa, Chang95, Yuen3, and CMS remained significant (detailed results in Supplementary Table 5).

Finally, in an additional analysis we split stage 2 and stage 3 patients and separately evaluated each of the classifiers in the two cohorts. Three of the classifiers had over 50% improved predictive accuracy in stage 2 patients including Chang95 (p = 3.8E-06, HR = 3.11), Marisa (p = 2.4E-03, HR = 3.3) and ODXcolon (p = 5.6E-06, HR = 2.61), while Yuen3 was the only classifier delivering better results in stage 3 patients (p = 3.1E-4, HR = 3.1).

### Effect of signature member genes on overall prognostic power

To evaluate the power of the genes included in the classifiers, we first performed a univariate survival analysis for all genes included. In this approach, the median expression of the genes was used to define two groups. All together 29% of the genes reached significance at p < 0.05 and 16% did so at p < 0.01. We then evaluated the percentage of the genes that were significant within each of the classifiers. The highest proportion of significant genes was reached by the CIN25 signature (82%), followed by the Meta163 (78%), TCA19 (74%) and ODXcolon (73%) gene sets. The lowest proportions were achieved by the V7RHS (14%), Oncodefender (20%) and CCHS (20%) signatures.

We compared the proportion of significant genes to the HR values achieved by the classifiers using Spearman Rank correlation. More significant genes increased the classification power (correlation coefficient = 0.47, p = 0.014, see Fig. 3F).

### Assignment of preclinical cell line models

We identified 151 gene arrays from 61 unique cell lines meeting the eligibility criteria. Of these, 55 lines were published in the Cancer Cell Line Encyclopedia (GSE36133), 15 cell lines in GSE8332, 60 samples in the Cancer Cell Line Project (E-MTAB-37) and 21 in the GSE32474 dataset. There was no gene chip repeatedly published among the cell line arrays.

A classification for the most frequently utilized cell lines for each molecular classifier with mutation states for the most important genes is presented in Table 2. For this classification, “frequent utilization” was established as having more than 50 hits in PubMed as of 29.09.2015. A detailed classification for each cell line versus each classificator is listed in Supplementary Table 6.

**Figure 4:**
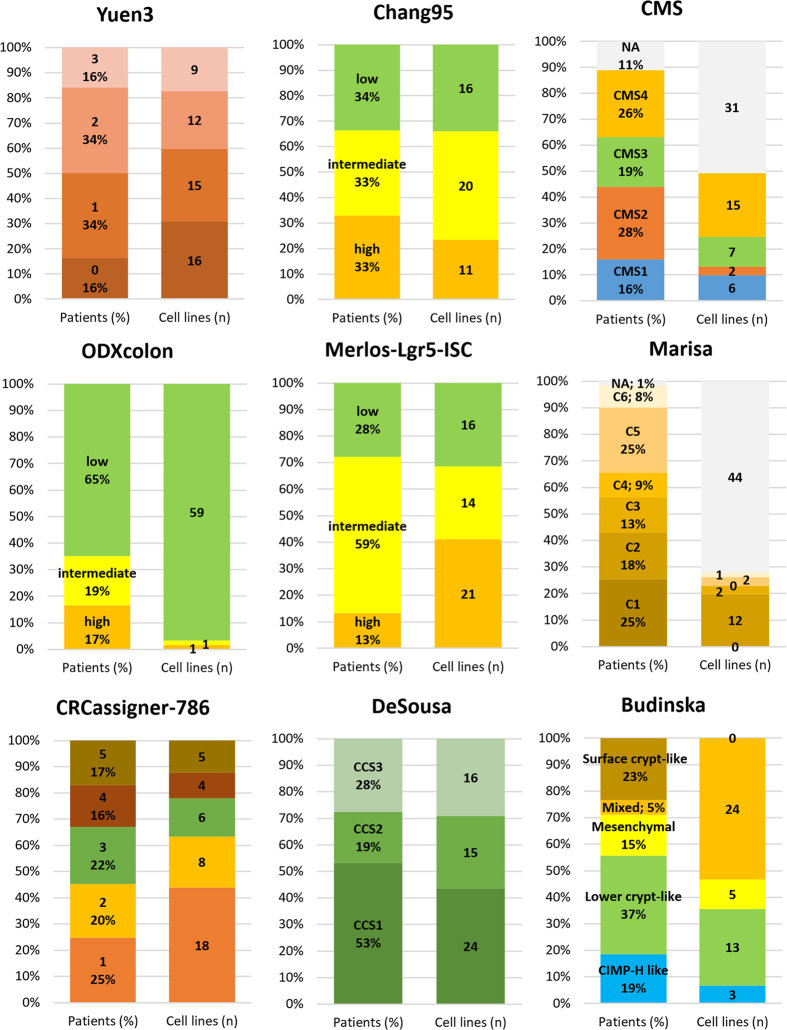
Linking prevalence and preclinical models. Subtype designation proportion for each classifier in all patients (left column, in percentage), and number of cell lines available for the given subtype (right column, n) for the best-performing classifiers. Grey corresponds to NA in each classifier. There are no representative cell line models for some of the subtypes.

To enable preclinical models to be linked to subtypes, we examined the proportion of subtype designation for each classifier in all patients and the number of cell lines available for the given subtype. Many classifiers have multiple suitable cell line models (e.g., Yuen3, Chang95, DeSousa, CRCassigner-786), some are unbalanced among the cell lines (e.g., for ODXcolon nearly all cell lines are classified as “low risk”), and repeated measurements in cell lines delivered contradictory outputs for a few classifiers (e.g., Marisa and CMS). The results for the best-performing classifiers are displayed in Fig. 4.

## Discussion

Truly personalized therapy is barely possible considering the high heterogeneity of colon cancer. However, one can assign a patient into a clinically and therapeutically relevant molecular subtype. Here, we compared 22 molecular subtypes in a large cohort of patients. In addition to ranking these, we also computed their concordance and identified preclinical models for their targeted evaluation. Although the total number of colon cancer patients with gene chip measurements was much higher (when combining all available datasets), we reduced the analysis to three overlapping array platforms. The use nearly identical gene chips enables a reliable cross-validation as different transcriptomic platforms measure the expression of the same gene with varying precision, on different relative scales, and with different dynamic ranges.

Currently, the NCCN guideline (www.nccn.org) lists three tests in its colon cancer guideline including OncotypeDX, ColoPrint and ColoGuideEx. These tests are suggested as additional data to inform the risk of recurrence over other risk factors, but to date no evidence supports their predictive value. We were not able to re-programme ColoPrint, a custom chip based assay utilizing FFPE samples^8^, because of insufficient description of the method. Interestingly, when comparing the classification delivered by OncotypeDX and ColoGuideEx, both reached statistical significance. However, at the same time only minimal concordance in sample designation was observed. This observation suggests the investigation of distinct pathways by these classifiers.

One of the major driving forces behind the development of subtype classification is the lack of clear guidelines for the administration of adjuvant chemotherapy for stage II and stage III colon cancer patients. For stage II patients, chemotherapy is not routinely offered unless they have a high-risk disease. For stage III disease, adjuvant therapy is generally recommended unless the patient is over 75 years of age. However, despite improvements in surgery, approximately 20% of colon cancer cases relapse, so it will be imperative to identify patients at high risk.

In line with this need, for the majority of the algorithms the goal of the subdivision is to identify patient sets with different prognostic features. However, independent validation has been performed for few of the studies, including ColoPrint^9^ and ColoGuidePro^10^. However, the number of samples utilized was very low in each case (n = 62 and n = 107, respectively). One previous study attempted to compare the prognostic performance of five colon cancer classifiers in a set 229 and 168 patients^11^. In that study, only two classifiers, OncotypeDX and MDA114, delivered a robust result. Moreover, despite the minimal overlap of utilized markers, the two classifiers had significant concordance when assigning the patients into risk categories.

On the other hand, classifications are not necessarily informative in terms of treatment efficacy. An example for this is a recently published study showing that patients with CMS4 had poor prognosis but didn’t benefit of intensive adjuvant therapy^12^.

In contrast to intuitive expectations, the overlap among individual markers involved in the molecular classifiers is rather limited. Only five genes (REG4, ASCL2, VAV3, C10orf99 and CYPB1) were included in at least six classifications. Previously, these genes were already reported to have distinct prognostic power. REG4 positivity by IHC was proven to have favourable impact on overall survival in non-mucinous CRC^13^. Patients with higher expression of the VAV3 oncogene had a poorer disease-free survival rate^14^. The ASCL2 transcription factor has an active role in specifying intestinal stem-cell identity and promotes cell proliferation. Higher levels of ASCL2 have been linked to worse prognosis^3^. C10orf99 (also known as CSBF) is down-regulated in colon cancer^15^.

The three highest scoring genes include CTGF (Connective Tissue Growth Factor), GADD45B (Growth Arrest and DNA-Damage-inducible, beta), and FAP (Fibroblast Activation Protein). Similarly to our results, the overexpression of CTGF was associated with shorter overall survival in multiple colon cancer subgroups^16^. Likewise, high expression of FAP has also been described to lead to poor patient prognosis^17^. Oncogenic K-RAS was associated with transcription of the antiapoptotic GADD45B^18^. However, no direct correlation between survival and GADD45B expression in colon cancer has been described earlier.

Increasing the number of markers in a classifier did not provide better classification accuracy in a previous study focusing on breast cancer^19^. Here, we observed a similar trend as the best performing classifier (Yuen3) is based on only three genes. In our analysis, markers containing more significant genes *per se* in univariate analysis had higher prognostic power.

An alternative approach for patient stratification would be to avoid the utilization of a pre-selected set of genes to assign patients into subdivisions. Rather, a whole transcriptome gene expression signature could be applied to pinpoint patients similar to the investigated one, with prognostic expectations then being determined by further evaluating the clinical outcome of these similar patients as has been demonstrated recently^20^. However, to date no similar methodology has been proposed for colon cancer, thus we have not included such a model in our meta-analysis.

In summary, we present here a large-scale cross-validation of multigene colon cancer prognostic tools using a combination of multiple independent datasets. The results are useful for future clinical trials to select the most appropriate molecular subtype stratification. Our study will also be useful for selecting preclinical models corresponding to each subtype for biomarker selection, drug discovery and treatment stratification.

## Methods

### Database construction

We assembled a large database of colon cancer samples measured by Affymetrix HGU133A (GPL96), HGU133Aplus2 (GPL570) and HGU133Av2 (GPL571) microarrays by using the keywords “colon”, “cancer”, “GPL96”, “GPL571” and “GPL570” in GEO (http://www.ncbi.nlm.nih.gov/geo/). Only publications with raw data, clinical data preferably including survival length, and at least 30 patients were included. The only microarrays included were GPL96, GPL571 and GPL570 because these platforms are frequently used and they share 22,277 common probe sets.

After an initial quality control step, redundant samples (n = 777) were identified and removed from the combined database. The raw.CEL files were MAS5/RMA normalized in the R environment (www.r-project.org) using Bioconductor libraries (www.bioconductor.org). For studies not designating a probe set id preference, we employed JetSet to select the most reliable probe set for each gene^21^.

### Colon cancer subtypes

Studies publishing colon cancer classifiers were identified using PubMed (www.pubmed.com) using the search terms “*gene expression profiling*”, “*transcriptome*”, “*colorectal neoplasms*”, “*prognosis*”, and “*survival analysis*”. Eligibility criteria for the studies were utilization of clinical samples for the discovery of subtypes, English language and publication of the statistical method developed. The search was performed according to the PRISMA guidelines^22^. Table 1 contains the most important characteristics for each of the subtype determination studies.

Statistical computations were performed in the R statistical environment. We set up a statistical computation pipeline for the classifiers that were not accompanied by an R function. The R scripts of these are available at Github (https://github.com/CRCInvestigation). For each classifier, samples included in the original training sets were excluded from the performed validation analysis (with the exception of the CMS which already included all available sample in their validation). After classifying each sample in the complete validation dataset (n = 2,166), the overlap between the different subtype classifications was calculated using Cramer’s V.

### Budinska – unsupervised clustering of five subtypes

Budinska *et al.*^23^ characterized five CRC subtypes based on unsupervised clustering of genome-wide expression patterns. A multiclass linear discriminant was trained on core samples with 54 meta-genes (658 genes) as variables to assign new samples to one of the following subtypes: surface crypt-like, lower crypt-like, CIMP-H-like, mesenchymal and mixed.

### CCHS – hypoxia score with prognostic relevance

Genes expressed differentially under chronic hypoxia versus normoxic conditions were compared for their prognostic value in public gene expression data leading to the Colon Cancer Hypoxia Score (CCHS) model^24^. The CCHS score is determined as a multiple of the expression values of six genes: score = 1.301 + 0.543*[BCCIP] − 0.416*[BNIP3L] + 0.596*[GADD45B] + 0.538*[INSIG2] − 0.177*[TP53]. The CCHS model was evaluated in formalin-fixed paraffin-embedded (FFPE) samples, and patients with a CCHS score higher than 4.526 had significantly worse disease-free survival (DFS).

### Chang95 – high, low and intermediate risk of relapse

Chang *et al.* constructed a 95-gene gene chip based signature and a 4-gene IHC signature after a network analysis of previously published gene sets^25^. Here we examine the 95-gene signature, in which patients are divided into three groups based on the mean expression value of the signature.

### CIN25 – high or low chromosomal instability

In this meta-analysis of 12 cancer microarray data sets representing six cancer types, Carter *et al.*^26^ identified a 25-gene gene expression signature of chromosomal instability (CIN). When the average expression of these genes was higher than the median, the sample was expected to have a high CIN status and bad prognosis. For classification with the same set of genes, we used MAS5-normalized and median-centred gene expression values.

### CMS – four consensus subtypes

Four consensus molecular subtypes (CMSs) were derived in a cross-platform analysis involving six previously published independent classifiers^27^. The four CMSs include a microsatellite unstable (CMS1), a canonical (CMS2), a metabolic (CMS3) and a mesenchymal (CMS4) subtype, each with distinctive biological features. CMS1 had the worst survival, CMS3 and CMS4 intermediate and CMS2 the best. Approximately 13% of the samples investigated in the original study were not classified - these did not represent a fifth independent subtype but rather displayed mixed features of the previous ones.

The consensus molecular subtypes were identified using the same set of patients evaluated in our present cross-validation analysis – for this reason, it was not possible to compute the predictive power after exclusion of the training set for this classifier. In addition to the CMS, we also evaluated five of the six original classifiers (Budinska, Schlicker, CRCassigner.786, DeSousa and Marisa). It was not possible to re-programme the sixth subtype^28^ because of insufficient data published in the original study.

### ColoGuideEx – high and low risk of relapse

Agesen *at al.*^29^ developed a 13-gene gene classifier to improve risk stratification for stage II CRC patients. ColoGuideEx classifies samples into cohorts having high or low risk of relapse on a gene-by-gene basis. A cutoff gene expression value above the 80^th^ or below the 20^th^ percentile (depending on whether high or low expression is associated with high risk of relapse, respectively) is used for the classification. Then, the 13 genes are tallied – samples having at least five genes with a positive prognostic score are classified as having high risk of relapse. Due to the utilization of a larger dataset, the cutoff values at the 80^th^ and 20^th^ percentiles were different in our study.

### ColoGuidePro – poor and good prognosis

Sveen *et al.*^10^ aimed to improve prognostic stratification for patients with stage II and III colorectal cancer using a seven-gene signature. In this signature, higher expression of four genes (DMBT1, NT5E, SEMA3A, and WNT11) and lower expression of three genes (CXCL9, OLFM4, UGT2B17) were associated with poor prognosis. Patients are assigned to the poor prognostic group when expressing three or more genes in the 7-gene signature at levels associated with poor prognosis.

### CRCassigner – unsupervised analysis deriving five subtypes

Unsupervised clustering is useful for detecting new subtypes independent of known clinical and molecular parameters. Using consensus-based non-negative matrix factorization to cluster gene expression data, Sadanandam *et al.* have derived a signature capable of discriminating five subtypes including goblet-like, enterocyte, stem-like, inflammatory, and transit-amplifying subtypes^30^. With 786 genes, this classifier utilizes the most extensive list of probes. The classification itself is performed with a prediction analysis for microarrays algorithm.

### DeSousa – CIN and MSI in one classifier

De Sousa *et al.* recognized three main molecularly distinct subtypes using an unsupervised classification algorithm^31^. The subtypes were characterized as chromosomal-unstable, microsatellite-unstable and microsatellite stable carcinomas with a CpG island methylator phenotype. The classification was achieved with the prediction analysis of microarray package. Using the DeSousa2013 R package, we re-trained a PAM classifier with probes present on the HGU133A arrays.

### Marisa – six subtypes with dominant biological characteristics

The objective of this study was to establish a comprehensive molecular classification of colon cancer based on mRNA expression profile analysis with unsupervised consensus hierarchical clustering^32^. Samples are divided with the citccmst R package according to their main respective biological characteristics into six subtypes: CIN-ImmuneDown (C1), dMMR - deficient mismatch repair (C2), *KRAS*-mutant (C3), cancer stem cell (C4), CIN-WntUp (C5), and CIN-normal (C6). The classification itself was conducted with a standard distance-to-centroid approach.

### MDA114 – long and short survival groups

A random-variance t-test between two colorectal cancer groups revealed by unsupervised hierarchical clustering show distinct biological relation to prognosis^33^. For HGU133Plus2.0 arrays we employed all 114 genes of the signature, for HGU133A arrays – in line with the original article – we utilized the 80 probes shared between the U133A and U133Plus2.0 gene chips. The classificator was trained on the GSE17536 dataset as described in the original article.

### Merlos-Suarez – intestinal stem cell signature for three subtypes

Merlos-Suarez *et al.* reported a gene signature specific to adult intestinal stem cells (ISCs) predicting disease relapse in CRC^34^. The authors identified a global expression profile of EphB2 receptor overexpressing ISCs (29 genes) and Lgr5 overexpressing ISCs (64 genes). Based on the average expression of the signature, samples are enrolled into three groups in which the EphB-ISC and the Lgr5-ISC signatures show prognostic value.

### Meta163 –Dukes B and C classification

Dukes stage B and C cancers cluster into two groups resembling early-stage and metastatic tumours^35^. The PAM algorithm identified 128 genes (163 probe sets) capable of classifying individual intermediate-stage cancers into stage A-like/good prognosis or stage D-like/poor prognosis types. The training set comprised 188 Dukes A and D samples. We used RMA-normalized data, and only class predictions over 90% were scored.

### ODXcolon – low, intermediate and high risk of recurrence

The Oncotype DX assay^36^ is another RT-PCR based diagnostic test optimized for estimating risk of recurrence in stage II or III colon cancer from FFPE samples. This 7 + 5 (cancer-related and reference) gene test has been clinically validated using data from three prospectively designed studies. We previously demonstrated the reproducibility of the Oncotype DX recurrence score (RS) for breast cancer using Affymetrix gene chips^37^. Here, we have executed an analogous approach: the expression (ΔCt) values are calculated as ΔCt (geneX) = −15-(log2 (mean (housekeeping genes))-log2 (geneX)). Then, the computation is performed as in the original equation. In case the RS is less than 30, the patient is considered having “low” risk of recurrence, between 30 and 40 “intermediate”, and all above are at “high” risk.

### Oncodefender – prediction of recurrence

Oncodefender is a 5 + 5 gene RT-PCR based test optimized for FFPE samples to predict recurrence of lymph node-negative invasive colorectal carcinoma^37^. The prognostic value of the test has been validated in stage I CRC and stage II colon cancer samples. After RMA normalization, the expression values of five housekeeping genes (B2M, GUSB, POLR2L, PSMB6, and UBC) were extracted from the expression of five genes comprising the signature. The Oncodefender signature score = abs (BMI1 × VEGFA ÷ H3F3B) − abs (ETV6 × H3F3B ÷ RPS10) as described in the original article. Instead of using the originally published cutoff values, we used the median for risk prediction because this modification delivered higher discriminatory power.

### Popovici – surrogate measurement of BRAF status

Mutational status is already a key element in treatment decision making for colorectal cancer. Popovici *et al.*^38^ aimed to identify a surrogate signature for BRAF mutation status. BRAF-mutated colorectal cells harbour a KRAS-independent pathway-responsive gene signature in colorectal cancer^39^. The classification model of Popovici *et al.* consists of two groups of genes (G1 and G2), and the prediction is made by comparing the averages of these groups. Samples in which the average of G1 is smaller than the average of G2 are predicted to be BRAF-mutant-like, otherwise they are wild-type-like.

### Schetter – high and low inflammatory risk score

In their study, Schetter *et al.*^40^ identified an RT-PCR based inflammatory gene expression signature with prognostic power in colon cancer. After normalization of the gene expression to 18 S rRNA, two models, the “noncancerous risk model” and the “tumour risk model” are combined in their setup. Individuals who have higher than median values for both models are classified as having a high inflammatory risk score (IRS). All others are considered low.

### TCA19 – a risk score for stage III patients

Using RNAseq data from normal colon, primary CRC, and liver metastases, Kim *et al.* developed a 19-gene risk score^41^. The risk score for each patient is calculated as the sum of each gene’s score, which is derived by multiplying the expression level of a gene by its corresponding Cox regression coefficient from the signature from the CIT cohort (GSE39582). Patients are divided into two groups using the median cut-off of the risk score. Patients designated as high risk with stage III disease showed significantly inferior DFS than low-risk patients, whereas there was no significant difference in DFS between high- and low-risk patient groups in stage II disease.

### Yuen3 – four cohorts with three genes

Yuen *et al.* combined the expression of three genes (TAZ, AXL, and CTGF) into a prognostic classification. Expression levels are divided into high and low groups using the median expression as the cut-off, and patients are enrolled into one of 4 groups based on the number of overexpressed genes (0/1/2/3)^42^. Patients who had higher than median expression for all 3 genes had the worst prognosis.

### V7RHS – high or low relapse hazard score

The RT-PCR based V7RHS test was designed by Jiang *et al.*^43^ to use FFPE samples to predict recurrence in stage II colon cancer. Expression (ΔCt) values of seven genes are utilized to calculate a relapse hazard score (RHS) for each patient. Here, we generated ΔCt values by extracting the RMA-normalized, median-centred, log2-transformed gene expression levels of the housekeeping genes used in the original study (*ACTB*, *HMBS*, and *RPL13A*) from the expression of the seven genes in the signature. Patients with higher than 0 RHS were classified as having high risk of recurrence.

### Watanabe-MSI and Watanabe-CIN – predicting MSI and CIN status

Instead of establishing new classifiers in an unsupervised analysis, Watanabe *et al.* aimed to utilize gene expression signatures to classify patients according to two established clinically useful parameters, microsatellite instability (MSI) and chromosomal instability (CIN). By comparing MSI and microsatellite stable (MSS) colorectal cancers using DNA microarray analysis, they identified 177 discriminating probe sets predicting MSI status with high accuracy^44^. MSI cancers have shown better prognosis compared to MSS cancers. Here, we performed the identical preprocessing steps described in the original article for both the training and test sets. We also trained the KNN classificator using the same dataset (GSE5445). The second gene signature classifies samples into CIN-high and CIN-low classes using microarray data^44^. The CIN classes correlate with survival in stage II and III colorectal cancer.

### Ranking of genes included in the classifiers

For each gene, we computed a score to represent the relative significance of the gene across all datasets. First, the relative proportion of each marker was computed in each gene signature by dividing 1 by the total number of genes included in the signature. Then, the gene score was computed as: “*gene score*” = [number of classifiers containing the gene]*[sum of gene proportion in each dataset].

### Comparison of classification performance

Cox proportional hazards regression and Kaplan-Meier plots were utilized for survival analysis using samples with published survival data (n = 1,405). In this analysis, the subtype designation was used to assign the samples to different cohorts. In case there were more than two subtypes, only the subtypes with the best and worst outcome were used to make comparison between all classifiers possible. Multivariate analysis was performed for each classifier with available prognostic parameters including MSI status, gender, MKI67 expression as a surrogate marker for proliferation and CDX2 expression^45^.

### Identification of representative cell line models

Gene expression signatures of colon cancer cell lines were screened in GEO and Array Express. Eligibility criteria included human origin, the availability of raw gene chip data derived using Affymetrix HGU 133 plus 2.0 series of arrays and strict identification of the cell line preferably with ATCC identifiers.

Some of the cell lines were published in multiple studies – in these cases, we computed a classification for each sample and used a tally for the final result: in case at least 60% of the arrays for the same cell line delivered the identical result, then this was considered robust. In all other cases, the given classifier was designated as “NA” for the cell line. Mutational status of KRAS, BRAF, PIK3CA, PTEN, TP53 and APC in each cell line was retrieved from data published in COSMIC (http://cancer.sanger.ac.uk/cosmic) or the Cancer Cell Line Encyclopedia (http://www.broadinstitute.org/ccle).

## Additional Information

**How to cite this article**: Sztupinszki, Z. and Győrffy, B. Colon cancer subtypes: concordance, effect on survival and selection of the most representative preclinical models. *Sci. Rep.* **6**, 37169; doi: 10.1038/srep37169 (2016).

**Publisher’s note**: Springer Nature remains neutral with regard to jurisdictional claims in published maps and institutional affiliations.

## Supplementary Material

Supplementary Information

Supplementary Table 1

Supplementary Table 2

Supplementary Table 3

Supplementary Table 4

Supplementary Table 5

Supplementary Table 6

## Figures and Tables

**Table 1:** Summary of the implemented classifiers.

Test		Eligibility		Classification technique				Original training set		Original validation set	
name	reference	FF/FP	stage	cohorts	methods for classification	gene n	platform	GSE datasets	size (n)	validation GSE datasets	size (n)
Budinska	23	FF, FP	II–III	5	linear discriminant analysis	658	ALMAC, U133 + 2, U133A	14333, 2109, 17537, PETACC3	425	4107, 4183, 10714, 15960, 13294, 18088, 26682, 26906, TCGA	720
CCHS	24	FF,FP	II–III	2	sum score, pre-computed cutoff	6	U133 + 2	13294, 5206, 17537, 17536	229	FFPE samples	126
Chang95	25	FF	I–III	3	median of mean expression	95	ALMAC, U133 + 2, U133A, HuEx-1_0-st	28702, 5206	188	17536, 17537, 14333, 37892, 12945, 41258, 24551, E-MTAB-863, E-MTAB-864	682
CIN25	26	FF	—	2	median of mean expression	25	U133 + 2, U133A, Rosetta	multiple (n = 18)	1,944	NA	NA
CMS	27	FF, FP	—	4	centroid-based predictor	693	Agilent, RNA–seq, Affymetrix	multiple (n = 18)	1,721	as of training (one dataset was split into equal training and validation)	1,721
ColoGuideEx	29	FF	II	2	number of genes exceeding the 80th and 20th percentile	13	HuEx-1_0-st	24550, 29638, 30378	112	24550, 29638, 30378, 14333, 17538	203
ColoGuidePro	10	FF	II–III	2	7	HuEx-1_0-st	30378	95	14333, 17538, 24550	290	
CRCassigner-786	30	FF	—	5	PAM	786	U133 + 2	13294, 14333	445	13294, 14333, 12945, 16125, 20916, 20842, 21510, TCGA, 28722	744
DeSousa	31	FF	—	3	PAM	146	U133 + 2	33114	90	14333, 17538, 13294, 13067, 5851, 28702, 35144, E-MTAB-991, TCGA	1,074
Marisa	32	FF	—	6	centroid-based predictor	57	U133 + 2	39582	443	13067, 13294, 14333, 17536/17537, 18088, 26682, 33113, TCGA	1,181
MDA114	33	FF	II–III	2	compound covariate predictor	114	U133 + 2	17536	179	17537, 12945, 14333	213
Merlos-EphB2-ISC	34	FF	—	3	average signature expression	29	Affymetrix mouse4302	Mouse dataset 6894	18	17538, 14333	340
Merlos-Lgr5-ISC	34	FF	—	3	average signature expression	64	Affymetrix mouse4302	Mouse dataset 6894	18	17538, 14333	340
Meta163	35	FF	II–III	2	PAM	128	U133 + 2	5206, 14333	188	14333	99
ODXcolon	36	FP	II–III	3	weighted score	12	RT-PCR based	—	1851	—	711
Oncodefender	46	FP	I–II	2	multiplication of signature genes	5	RT-PCR based	—	74	—	264
Popovici	38	FP, FF	II–III	2	closest mean expression	64	ALMAC-array	PETACC-3	688	2138, 17538, ALMAC	114
Schetter	40	FF	—	2	weighted sum	9	RT-PCR based	—	57	—	139
TCA19	41	FF	III	2	median	19	U133 + 2	39582	566	14333, 33113, 37892	449
Yuen3	42	FF	II–III	4	median per gene	3	U133 + 2	14333, 17538	458	—	—
V7RHS	43	FP	II	2	weighted sum	7	RT-PCR based	—	233	—	—
Watanabe-CIN	44	FF	II–III	2	SVM	112	U133 + 2	30540	845	14333	290

Abbreviations: FP: FFPE; FF: fresh frozen; U133: Affymetrix HG-U133A; U133 + 2: Affymetrix HG-U133Plus2.0; ALMAC: Affymetrix Almac Xcel Array for FFPE; Rosetta: Rosetta custom 25 K array.

**Table 2:**
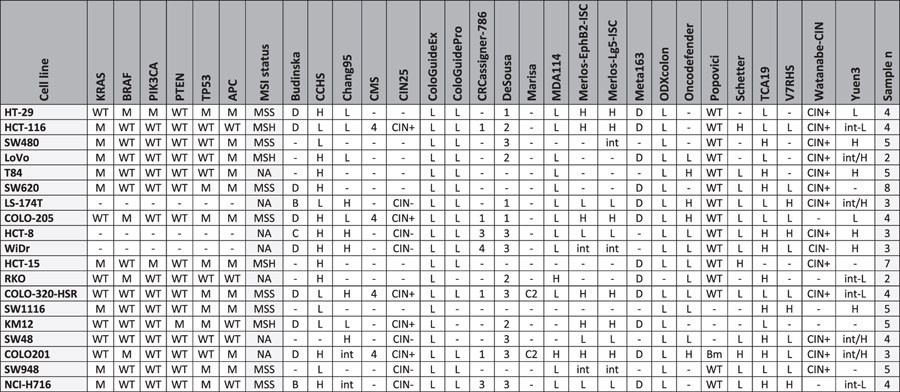
Assignment of preclinical models to the closest subtype.

Assignment of the most frequently utilized cell lines to each of the molecular classifiers based on a gene-array-based expression profile of the cell line. In case multiple arrays were utilized for a given cell line, and more than 40% of the arrays delivered different results, then the given cell line was not classified for that classificator. Mutation status is shown for the six most important genes, and MSI status is also shown for each cell line. *Abbreviations: M: mutant, WT: wild type, H: high, L: low, int: intermediate, Bm: BRAF mutant, MSH: MSI-High, MSS: MSI Stable, Sample n: number of gene chip samples providing expression data for the classification*.
