# Supplementary material for: Colon cancer subtypes: concordance, effect on survival and selection of the most representative preclinical models
Source: Sci Rep. 2016 Nov 16;6:37169. doi: 10.1038/srep37169 (PMC5111107; doi:10.1038/srep37169)
Supplement: Supplementary Information [file srep37169-s1.pdf]

Colon cancer subtypes: concordance, effect on survival and selection of most representative preclinical models

Zsófia Sztupinszki, Balázs Győrffy

SUPPLEMENTARY MATERIAL

Supplementary Figure 1. Individual Kaplan-Meier plots for each classifier in stage 2/3 patients.
Supplementary Figure 2. Forest plot for all classifiers in all samples.
Supplementary Figure 3. Forest plot for all classifiers in stage II patients.
Supplementary Figure 4. Forest plot for all classifiers in stage III patients.
Supplementary Figure 5. Individual Kaplan-Meier plots for each classifier in all samples.

Supplementary Table 1. Summary of the clinical parameters for each of the datasets included in the analysis.
Supplementary Table 2. Inclusion of each gene in each of the classifiers.
Supplementary Table 3. Assignment of each of the samples to each of the subtypes.
Supplementary Table 4. Cramer's correlation across all samples among all classifiers.
Supplementary Table 5. Results of the multivariate analysis.
Supplementary Table 6. Designation for each cell line for each suitable classifier.

TABLE OF CONTENTS

Supplementary Figure 1 .... **2**

Supplementary Figure 2 .... **24**

Supplementary Figure 3 .... **25**

Supplementary Figure 4 .... **26**

Supplementary Figure 5 .... **27**

Supplementary Figure 1

Individual Kaplan-Meier plots for each classifier in stage 2/3 patients.

21 Kaplan-Meier plots

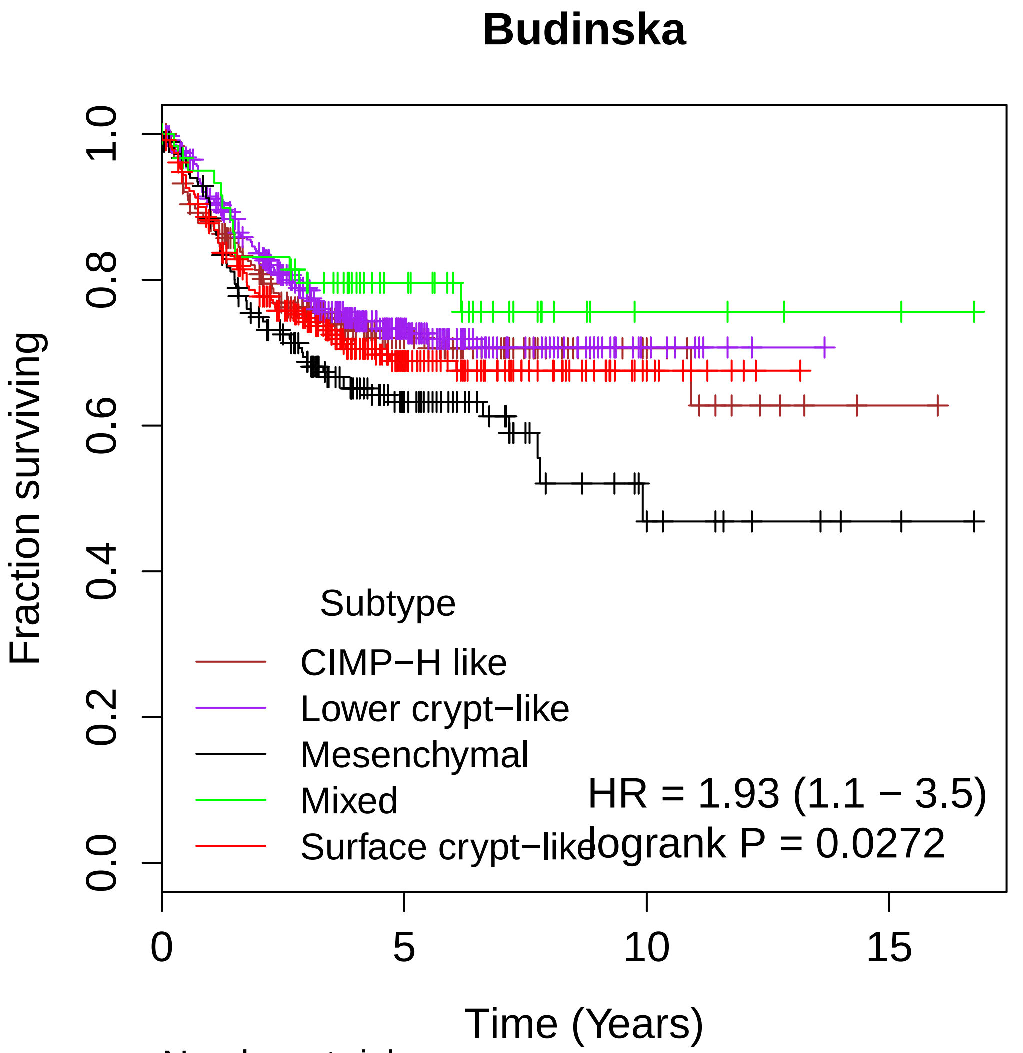

Number at risk

	0	5	10	15
CIMP−H like	184	58	12	1
Lower crypt−like	350	114	12	0
Mesenchymal	186	58	9	2
Mixed	61	26	4	2
Surface crypt−like	232	69	10	0

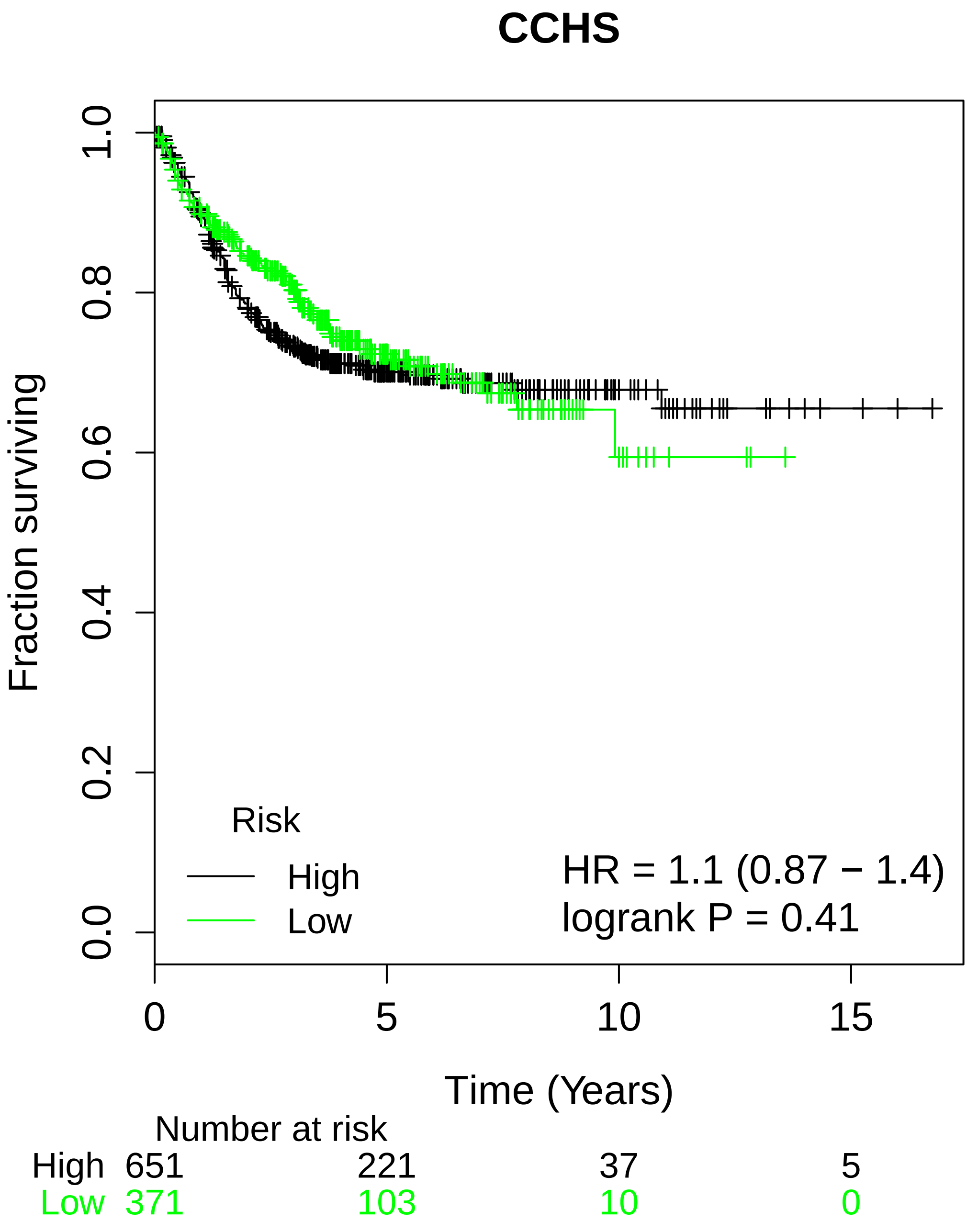
CCHS
Fraction surviving
1.0
0.8
0.6
0.4
0.2
0.0
Risk
High
Low
HR = 1.1 (0.87 − 1.4)
logrank P = 0.41
0
5
10
15
Time (Years)
Number at risk
High 651 221 37 5
Low 371 103 10 0

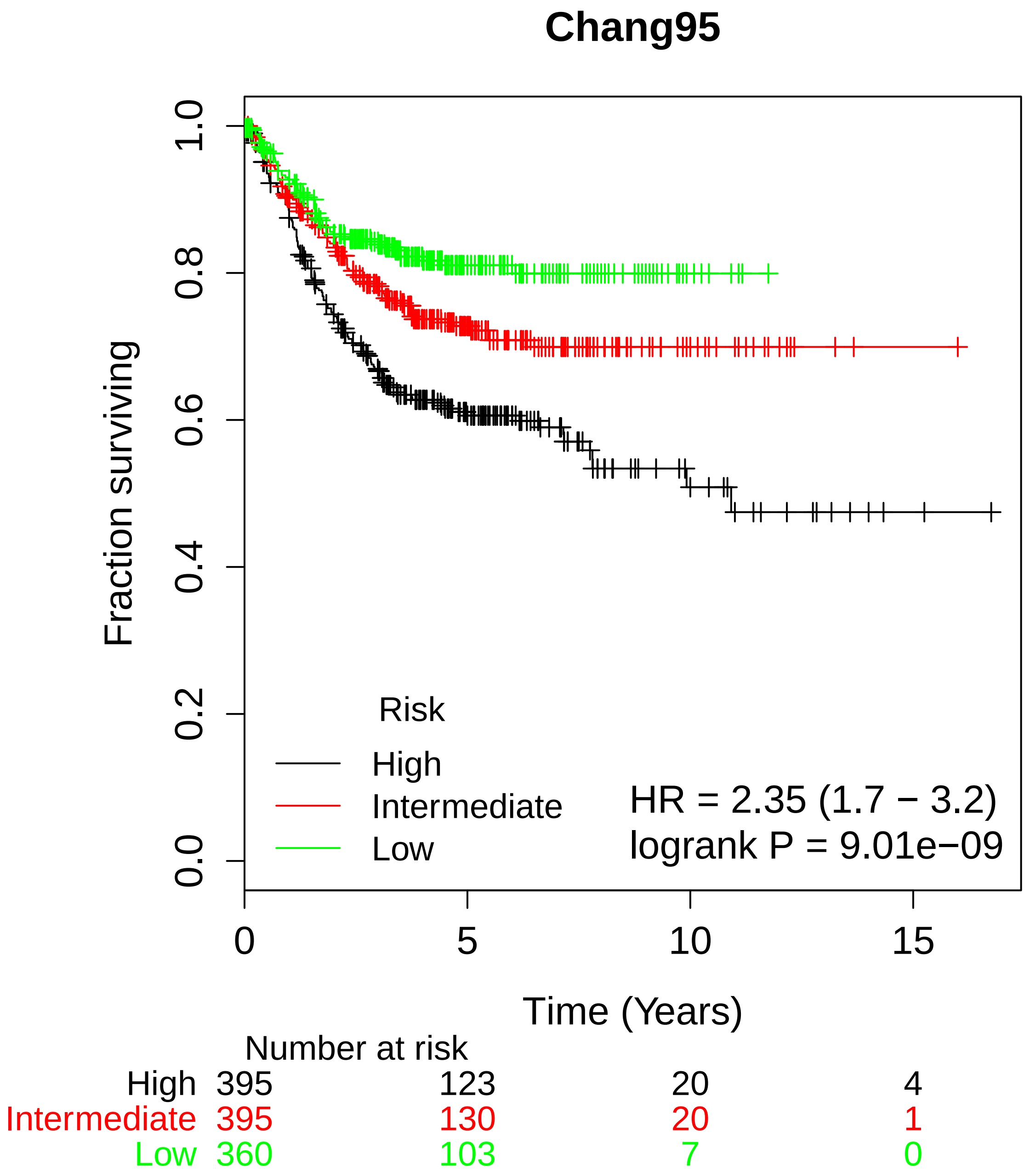
Chang95
Fraction surviving
0.0
0.2
0.4
0.6
0.8
1.0
0
5
10
15
Time (Years)
Risk
High
Intermediate
Low
HR = 2.35 (1.7 − 3.2)
logrank P = 9.01e−09
Number at risk
High 395 123 20 4
Intermediate 395 130 20 1
Low 360 103 7 0

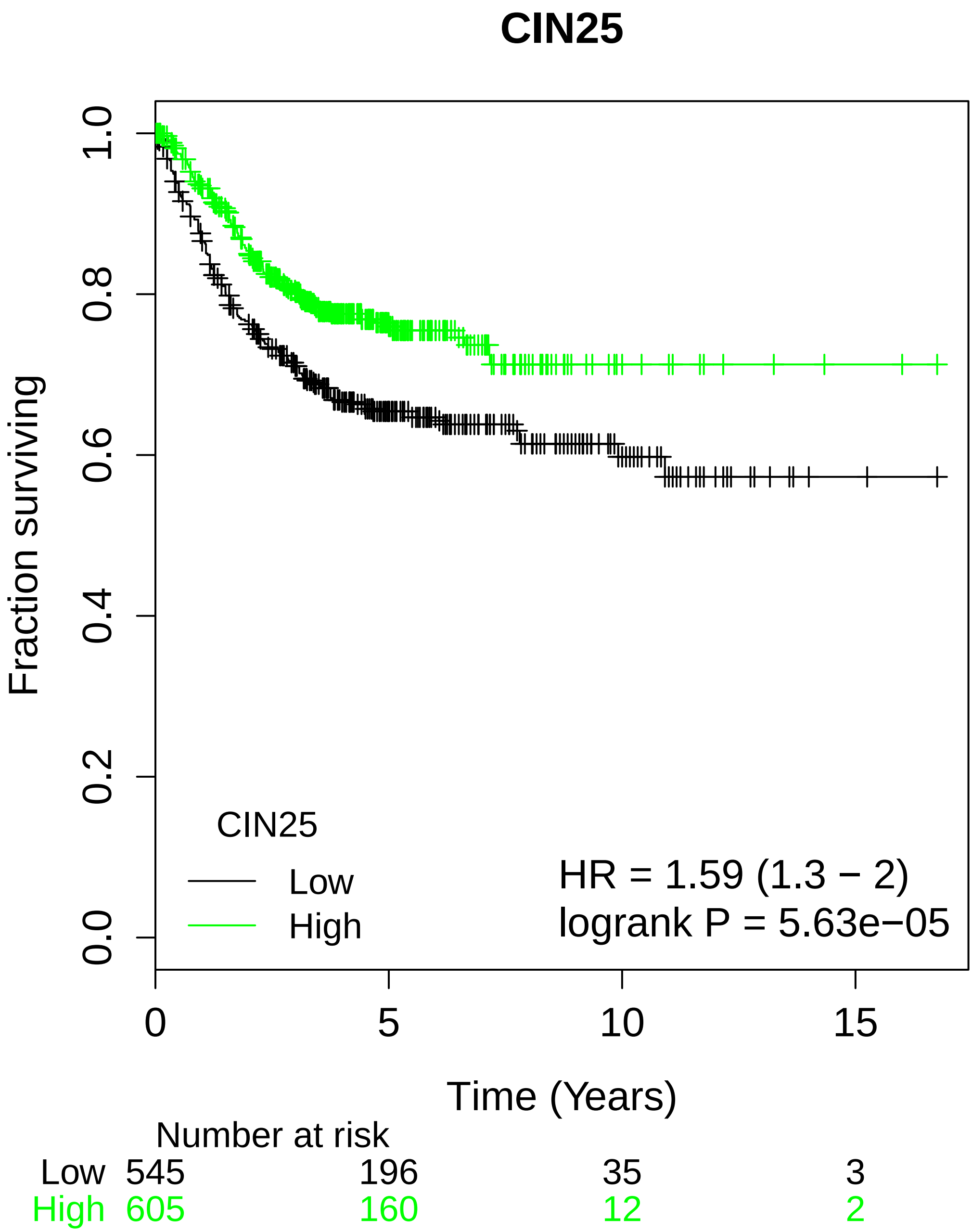

CIN25
Fraction surviving
1.0
0.8
0.6
0.4
0.2
0.0
CIN25
Low
High
HR = 1.59 (1.3 − 2)
logrank P = 5.63e−05
0
5
10
15
Time (Years)
Number at risk
Low 545 196 35 3
High 605 160 12 2

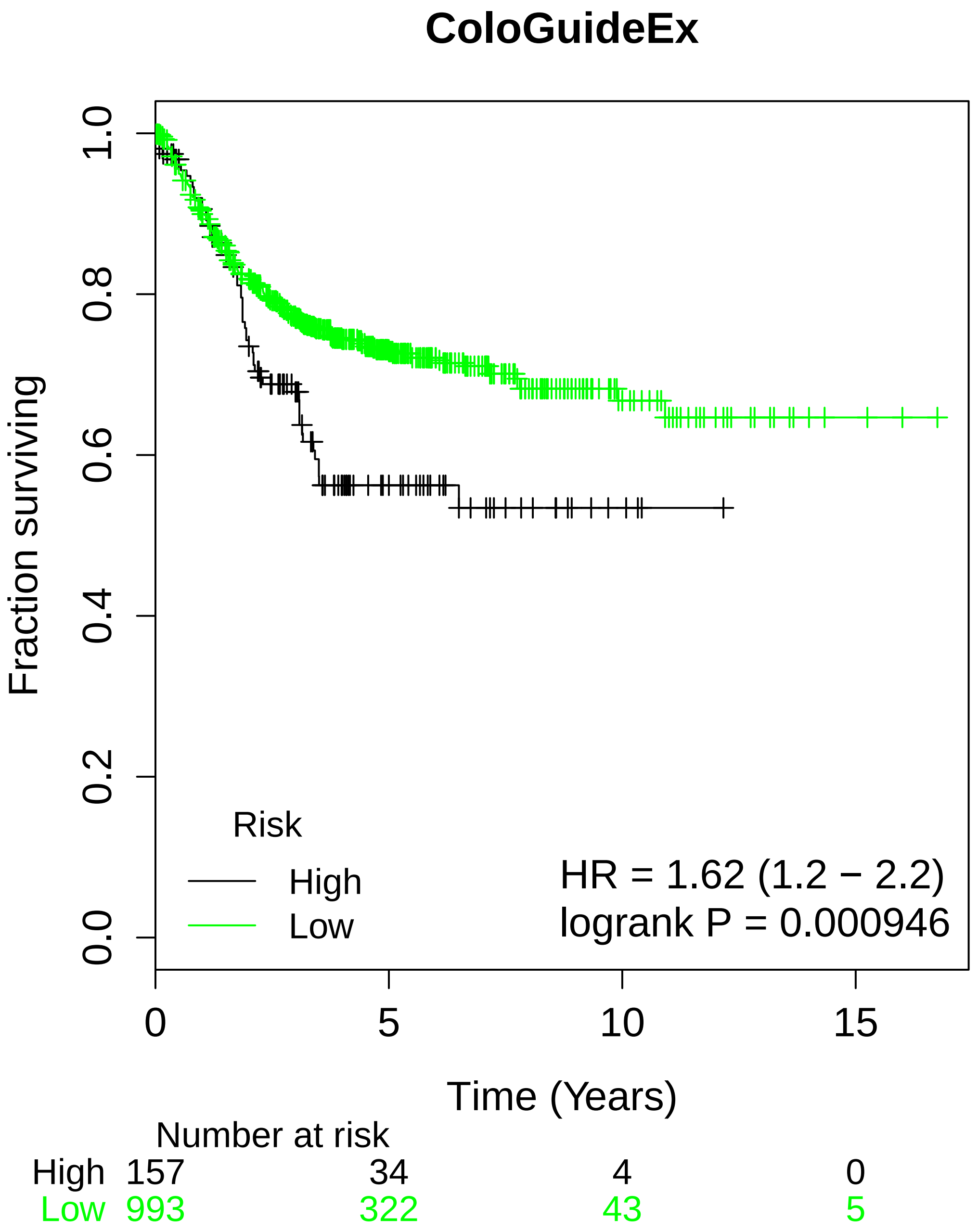
ColoGuideEx
Fraction surviving
1.0
0.8
0.6
0.4
0.2
0.0
Risk
High
Low
HR = 1.62 (1.2 − 2.2)
logrank P = 0.000946
0
5
10
15
Time (Years)
Number at risk
High 157 34 4 0
Low 993 322 43 5

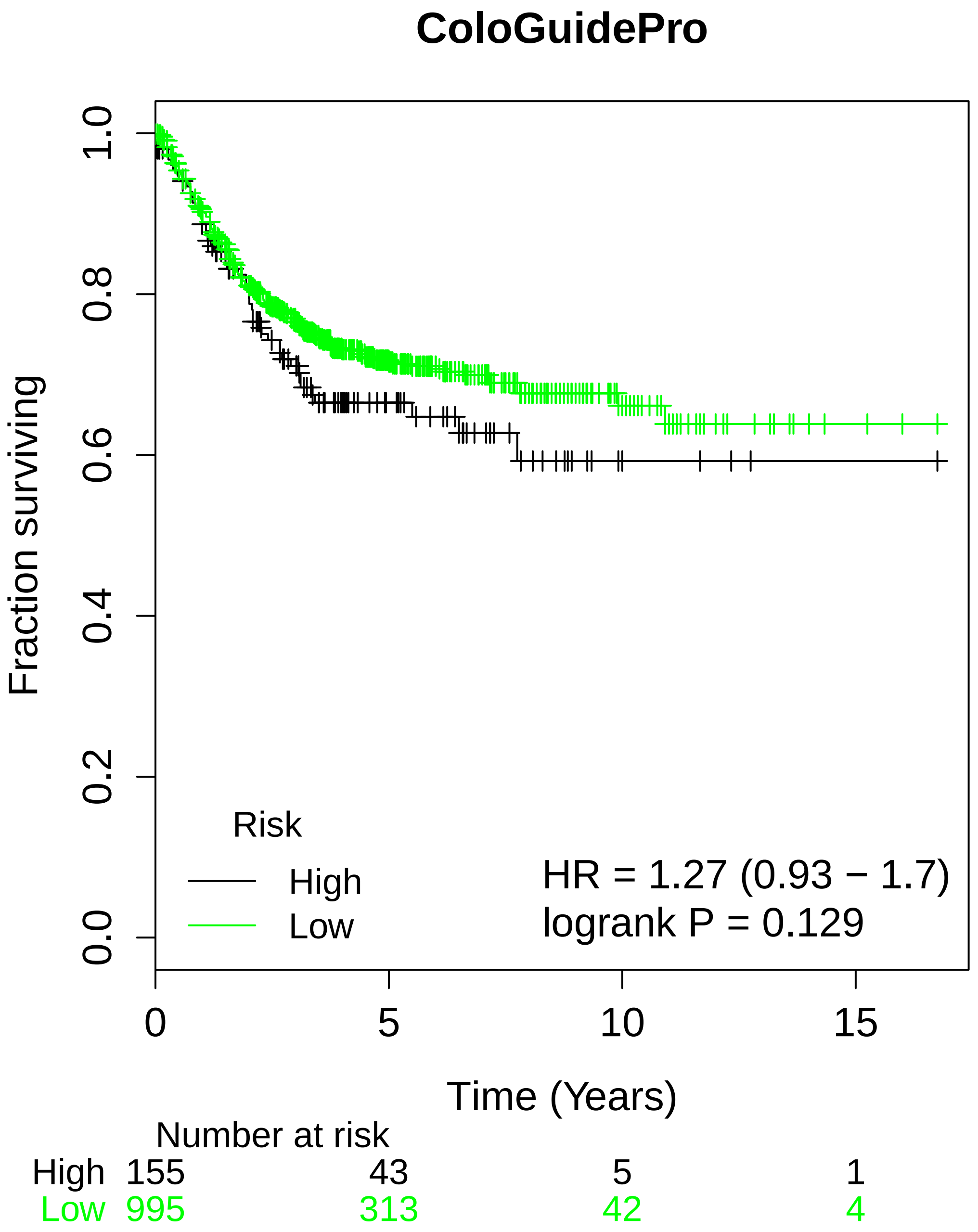
ColoGuidePro
Fraction surviving
0.0
0.2
0.4
0.6
0.8
1.0
0
5
10
15
Time (Years)
Risk
High
Low
HR = 1.27 (0.93 − 1.7)
logrank P = 0.129
Number at risk
High 155 43 5 1
Low 995 313 42 4

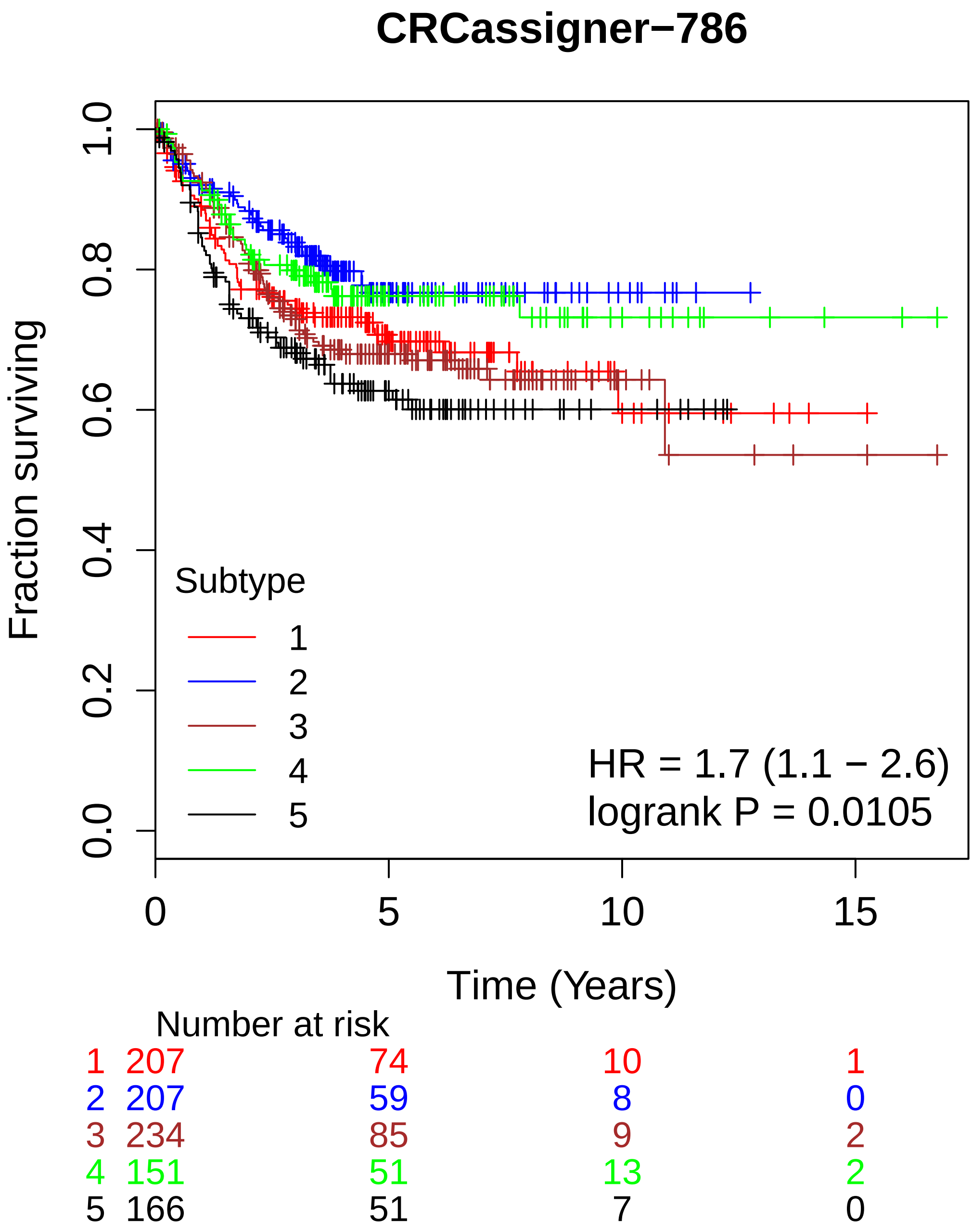
CRCassigner−786
Fraction surviving
1.0
0.8
0.6
0.4
0.2
0.0
Subtype
1
2
3
4
5
HR = 1.7 (1.1 − 2.6)
logrank P = 0.0105
0
5
10
15
Time (Years)
Number at risk
1 207 74 10 1
2 207 59 8 0
3 234 85 9 2
4 151 51 13 2
5 166 51 7 0

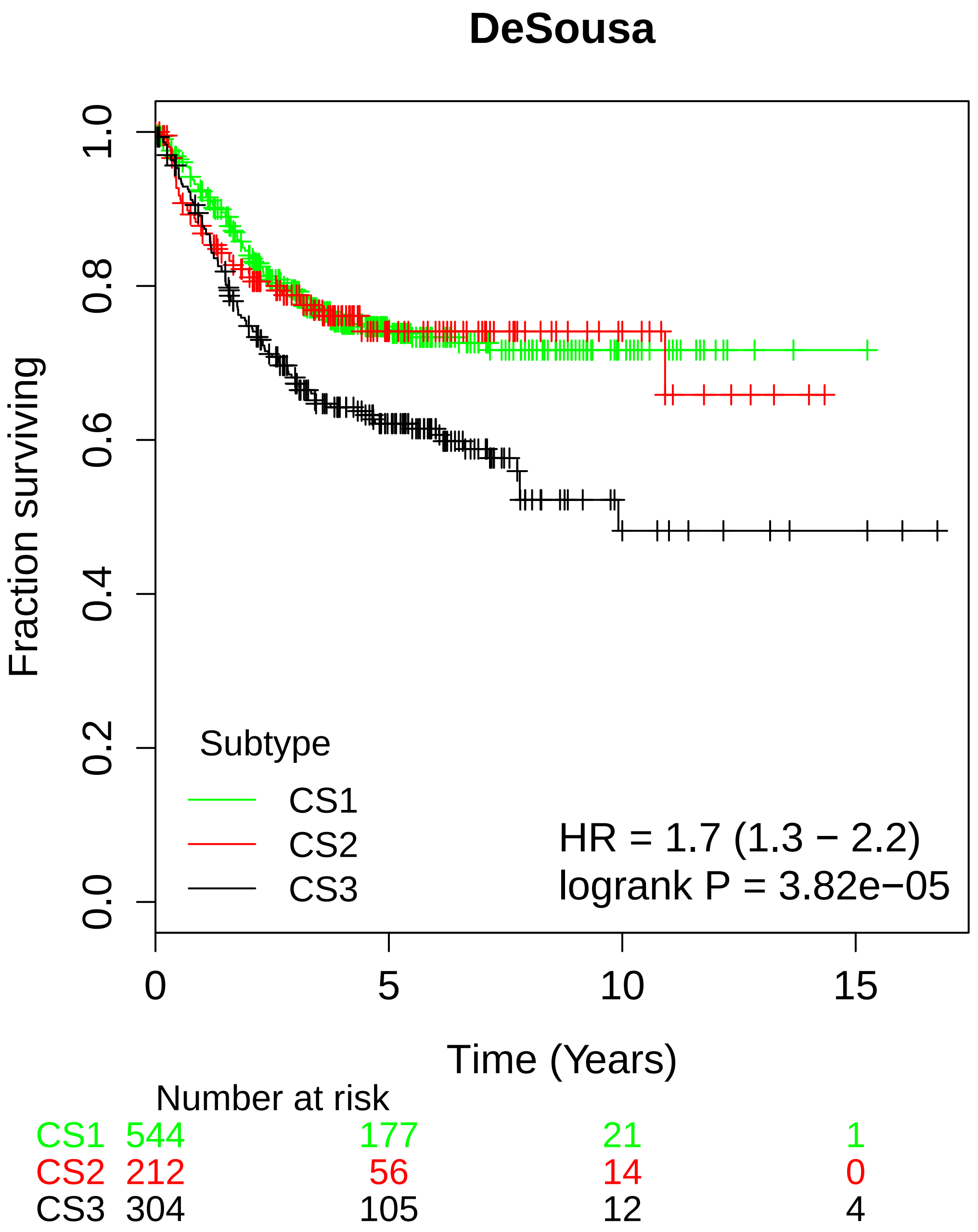
DeSousa
Fraction surviving
1.0
0.8
0.6
0.4
0.2
0.0
Subtype
CS1
CS2
CS3
HR = 1.7 (1.3 − 2.2)
logrank P = 3.82e−05
0
5
10
15
Time (Years)
Number at risk
CS1 544 177 21 1
CS2 212 56 14 0
CS3 304 105 12 4

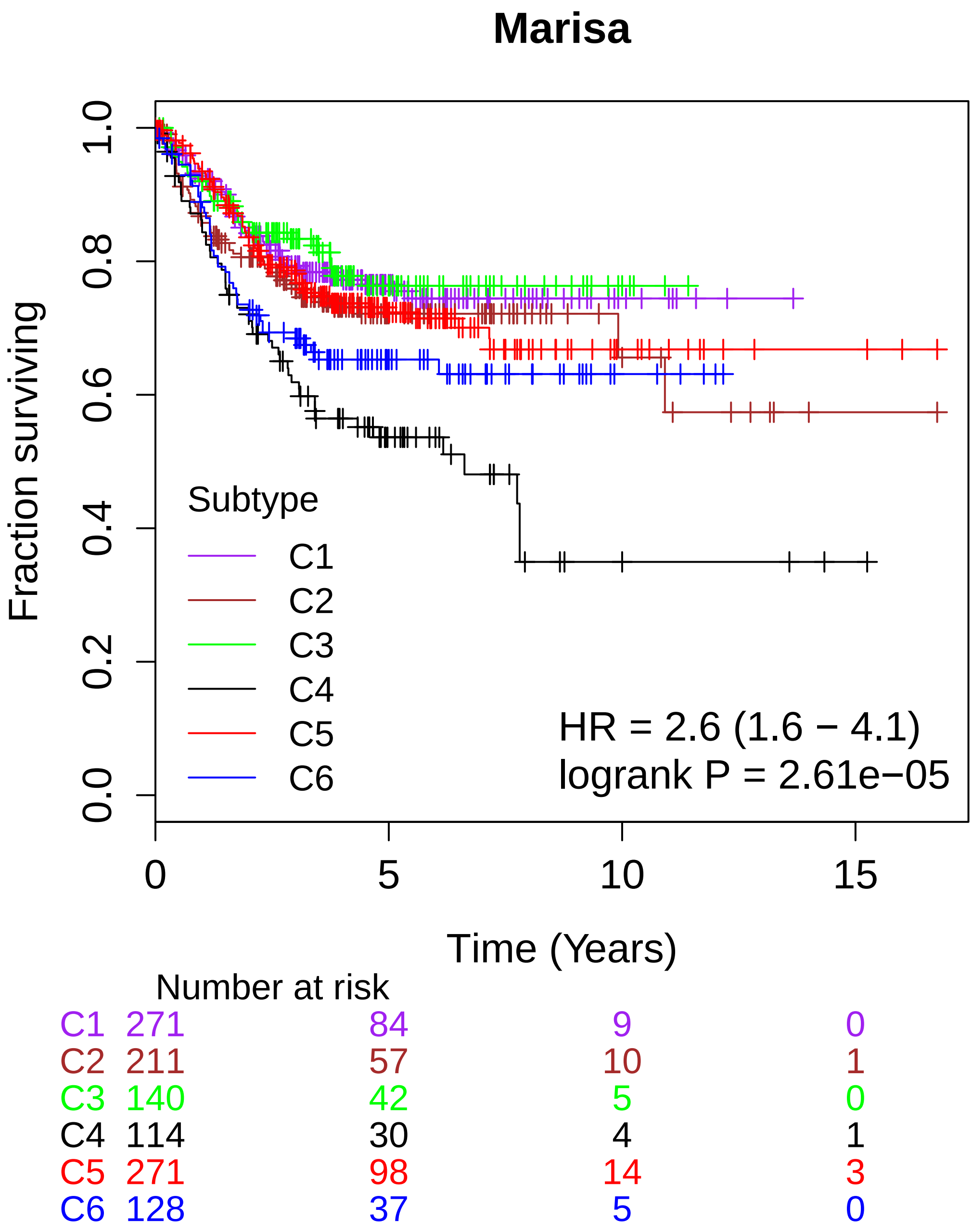
Marisa
Fraction surviving
0.0
0.2
0.4
0.6
0.8
1.0
0
5
10
15
Time (Years)
Subtype
C1
C2
C3
C4
C5
C6
HR = 2.6 (1.6 − 4.1)
logrank P = 2.61e−05
Number at risk
C1 271 84 9 0
C2 211 57 10 1
C3 140 42 5 0
C4 114 30 4 1
C5 271 98 14 3
C6 128 37 5 0

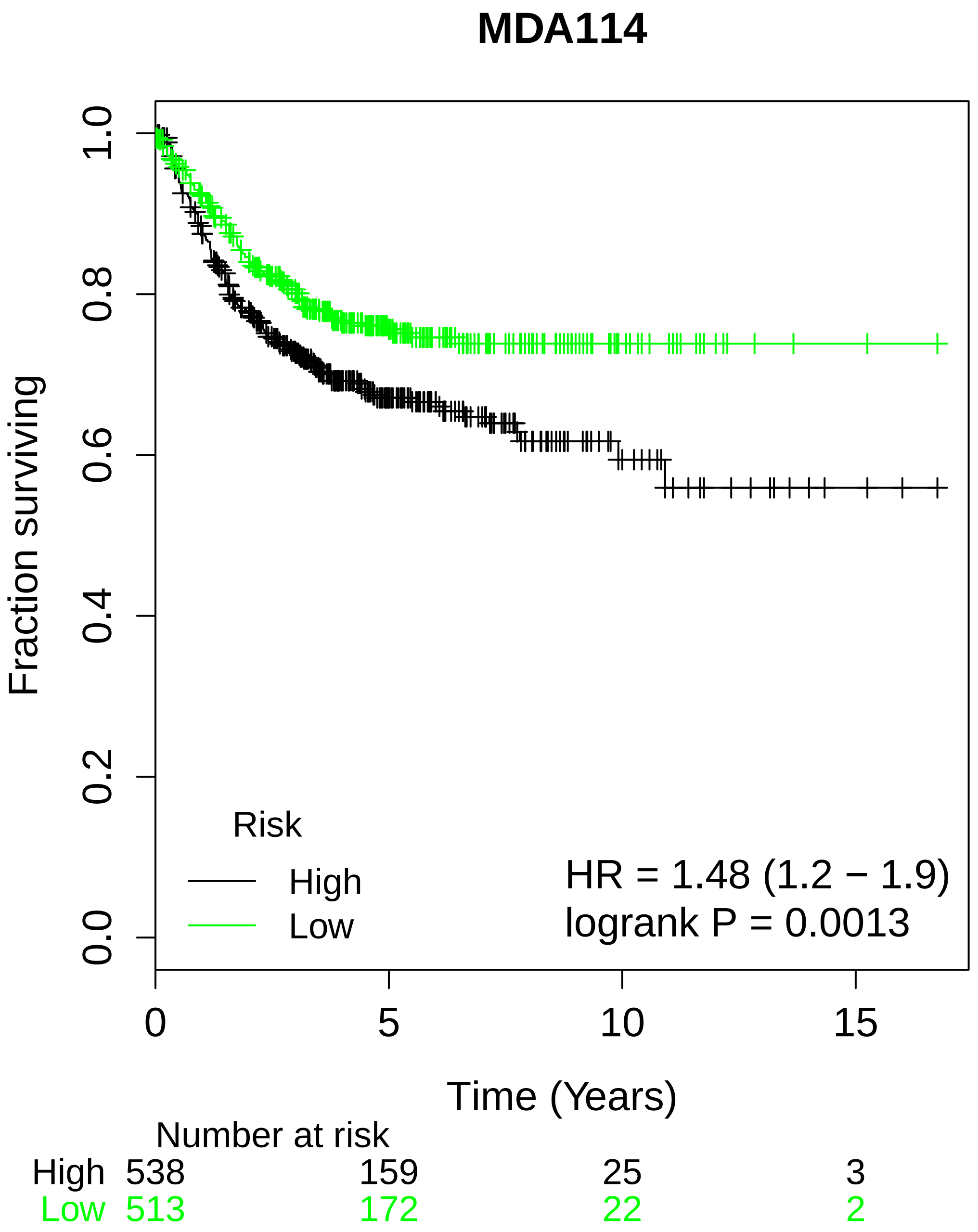
MDA114
Fraction surviving
1.0
0.8
0.6
0.4
0.2
0.0
0
5
10
15
Time (Years)
Risk
High
Low
HR = 1.48 (1.2 − 1.9)
logrank P = 0.0013
Number at risk
High 538 159 25 3
Low 513 172 22 2

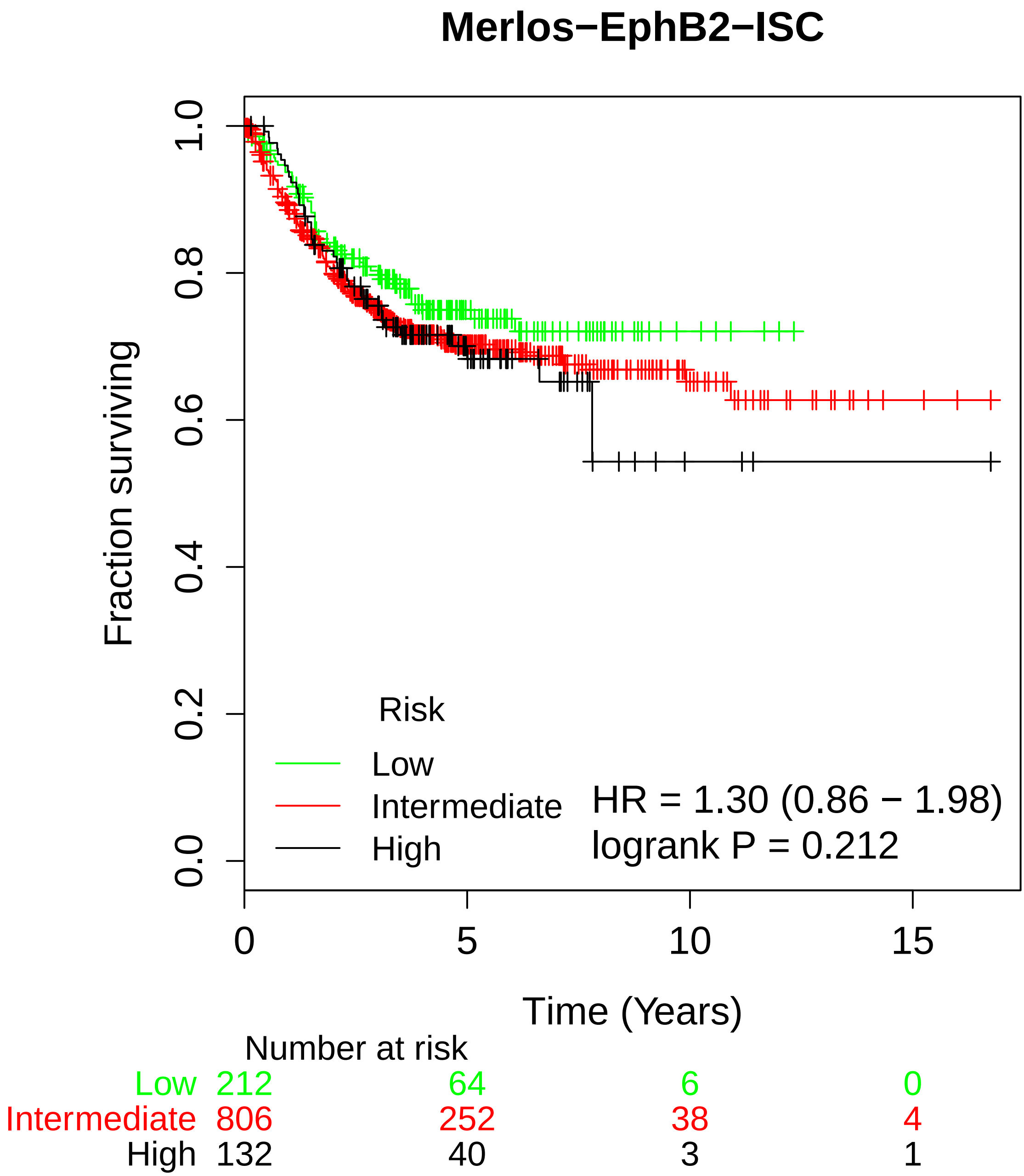

Merlos−EphB2−ISC
Fraction surviving
1.0
0.8
0.6
0.4
0.2
0.0
Risk
Low
Intermediate
High
HR = 1.30 (0.86 − 1.98)
logrank P = 0.212
0
5
10
15
Time (Years)
Number at risk
Low 212 64 6 0
Intermediate 806 252 38 4
High 132 40 3 1

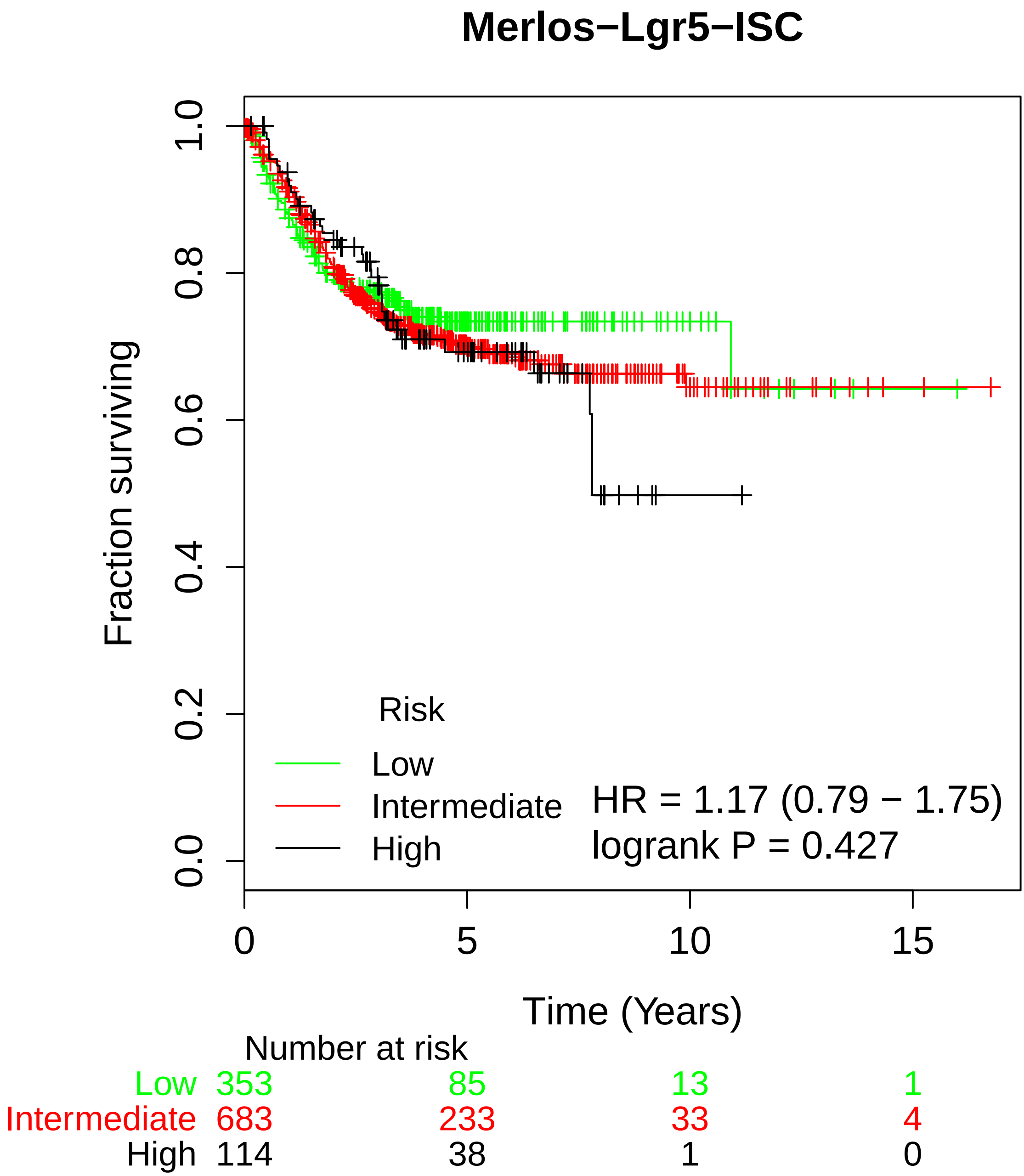

Merlos−Lgr5−ISC
Fraction surviving
1.0
0.8
0.6
0.4
0.2
0.0
Risk
Low
Intermediate
High
HR = 1.17 (0.79 − 1.75)
logrank P = 0.427
0
5
10
15
Time (Years)
Number at risk
Low 353 85 13 1
Intermediate 683 233 33 4
High 114 38 1 0

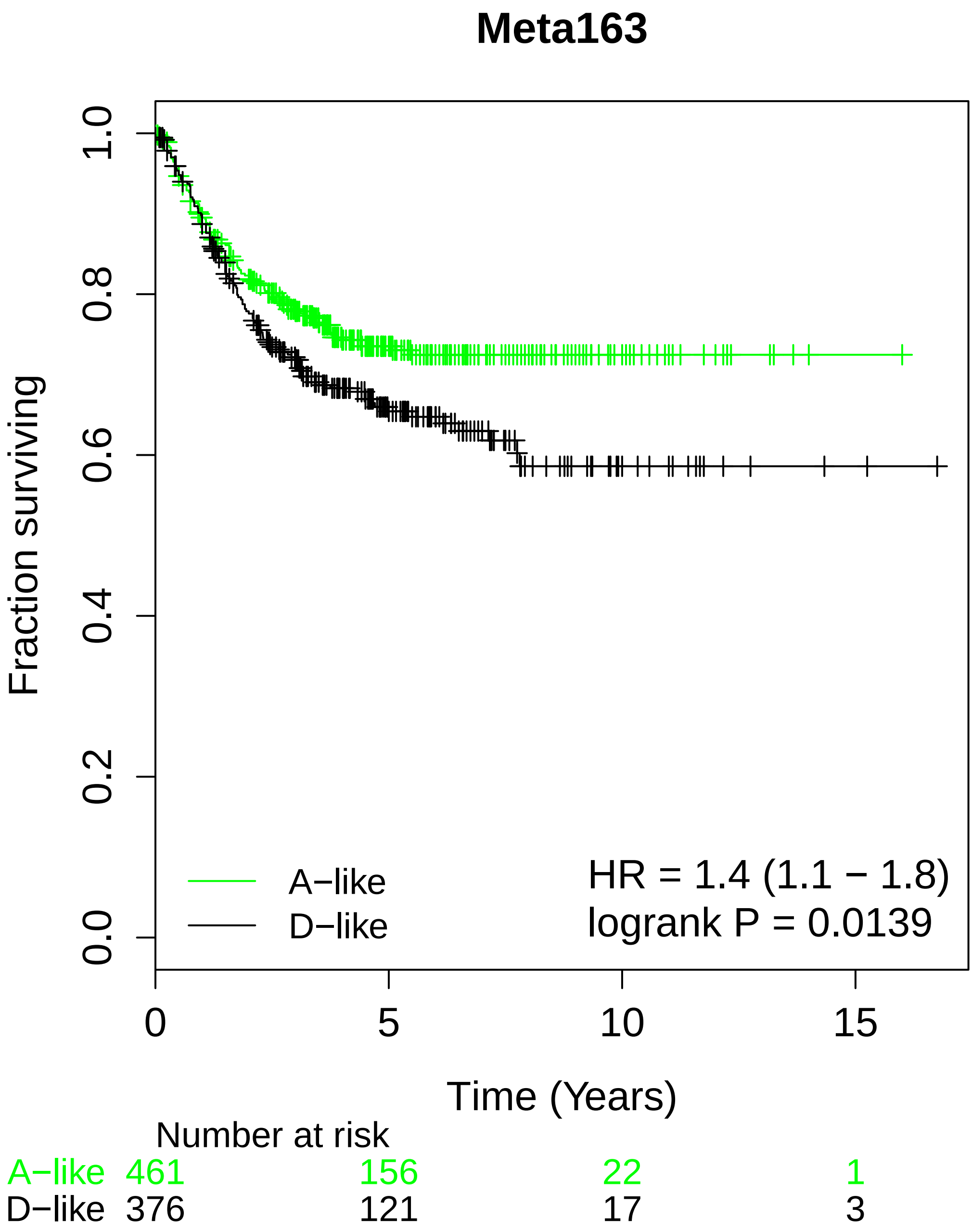
Meta163
Fraction surviving
1.0
0.8
0.6
0.4
0.2
0.0
0
5
10
15
Time (Years)
A−like
D−like
HR = 1.4 (1.1 − 1.8)
logrank P = 0.0139
Number at risk
A−like 461 156 22 1
D−like 376 121 17 3

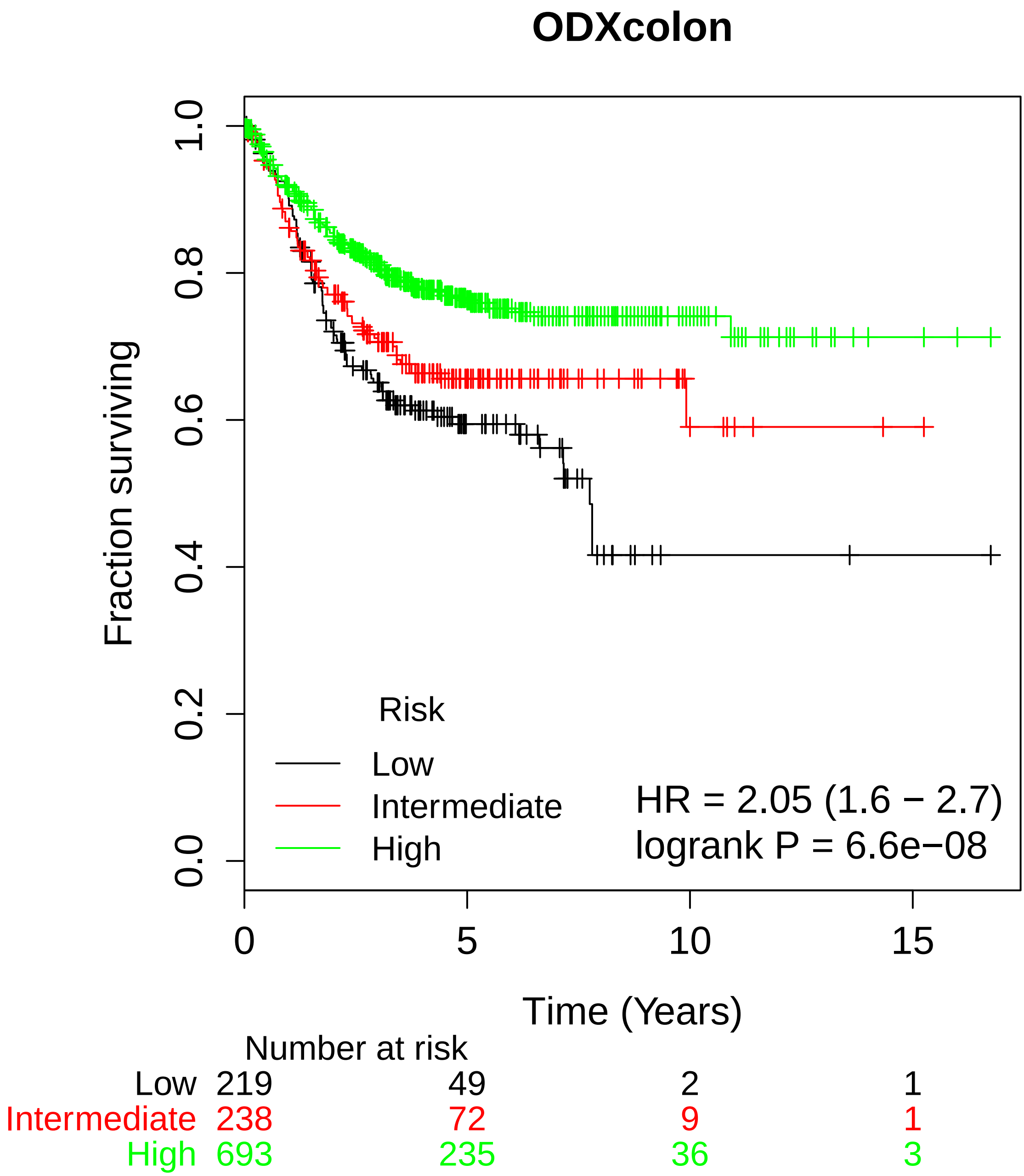
ODXcolon
Fraction surviving
1.0
0.8
0.6
0.4
0.2
0.0
Risk
Low
Intermediate
High
HR = 2.05 (1.6 − 2.7)
logrank P = 6.6e−08
0
5
10
15
Time (Years)
Number at risk
Low 219 49 2 1
Intermediate 238 72 9 1
High 693 235 36 3

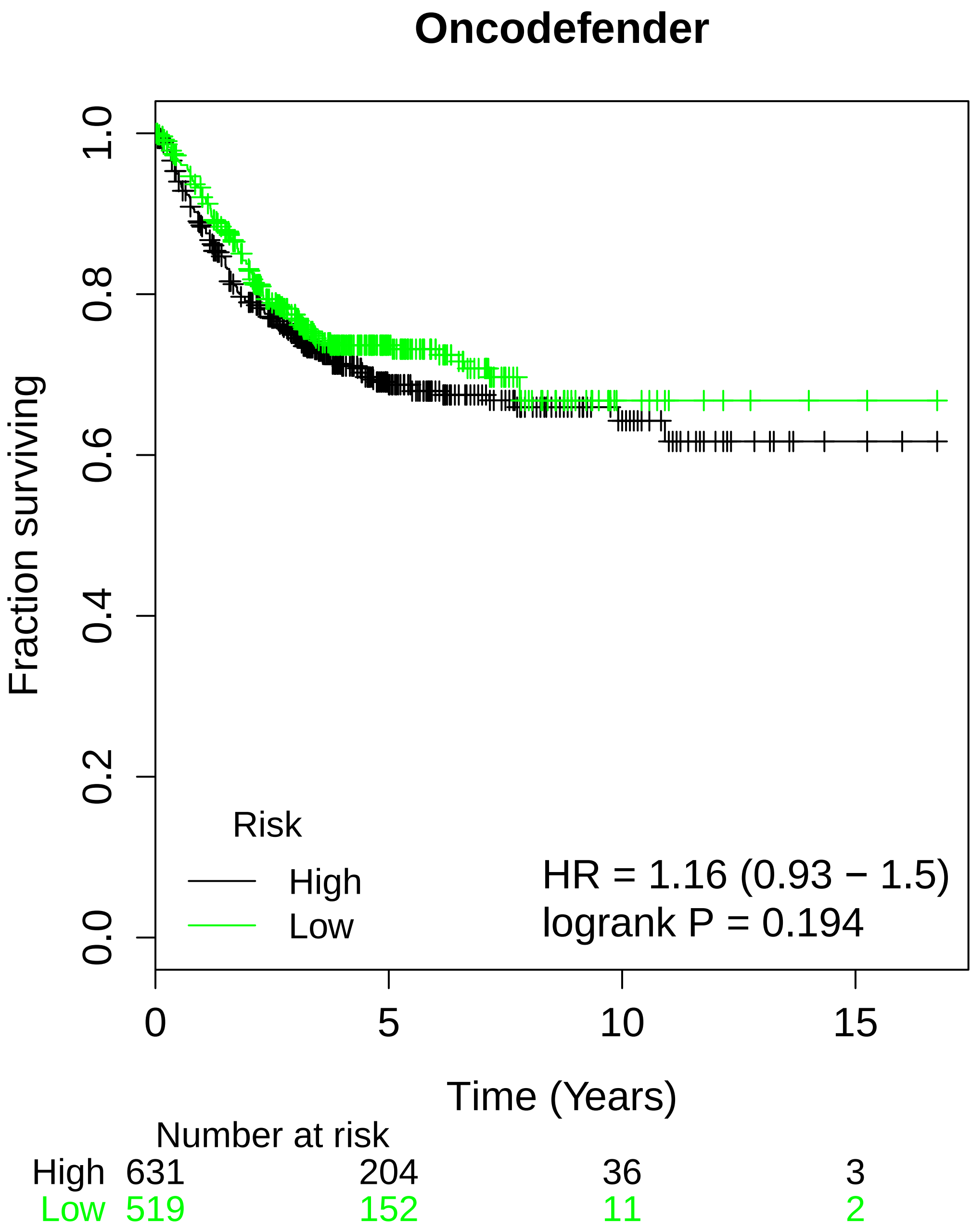
Oncodefender
Fraction surviving
1.0
0.8
0.6
0.4
0.2
0.0
Risk
High
Low
HR = 1.16 (0.93 − 1.5)
logrank P = 0.194
0
5
10
15
Time (Years)
Number at risk
High 631 204 36 3
Low 519 152 11 2

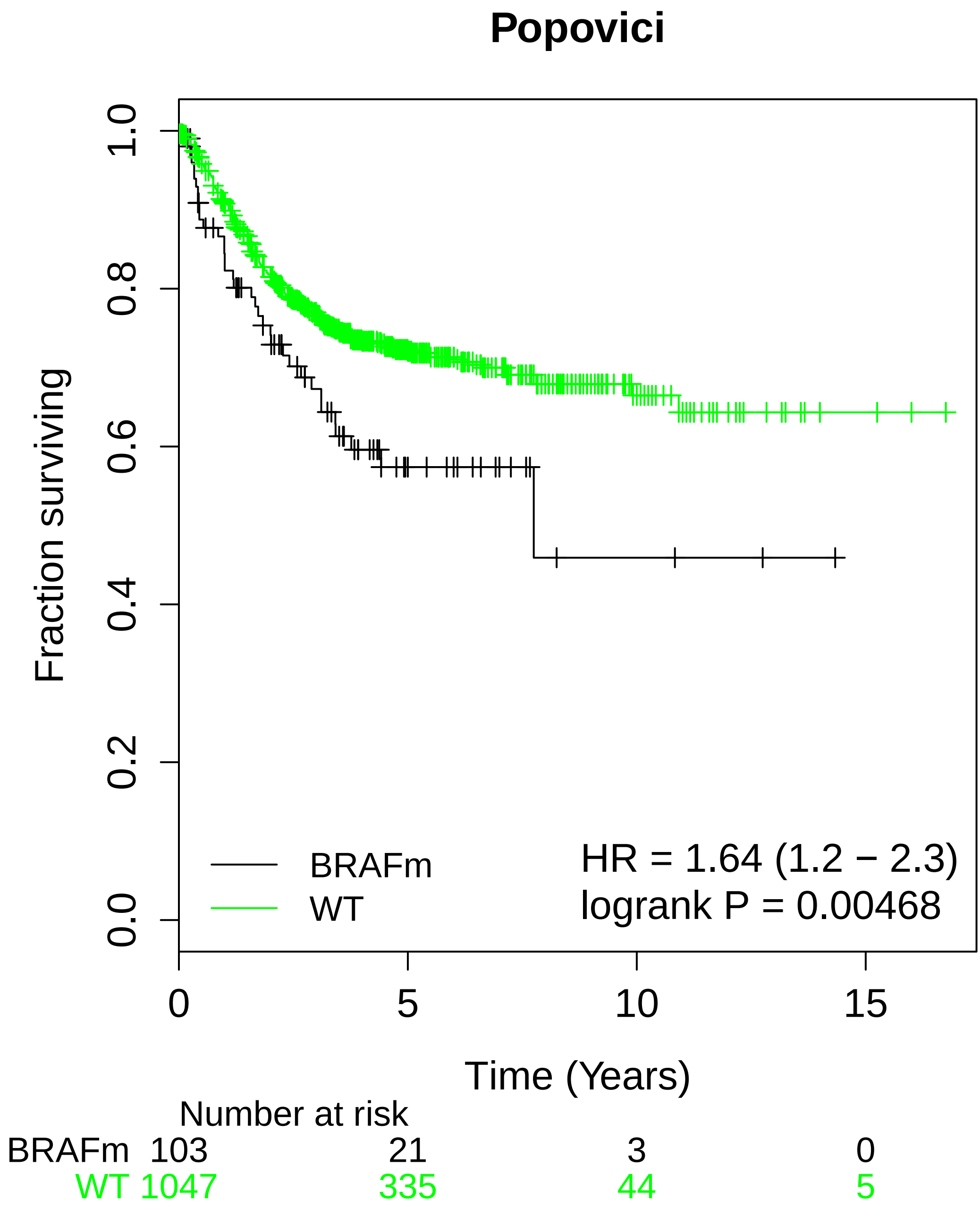
Popovici
Fraction surviving
0.0
0.2
0.4
0.6
0.8
1.0
0
5
10
15
Time (Years)
BRAFm
WT
HR = 1.64 (1.2 − 2.3)
logrank P = 0.00468
Number at risk
BRAFm 103 21 3 0
WT 1047 335 44 5

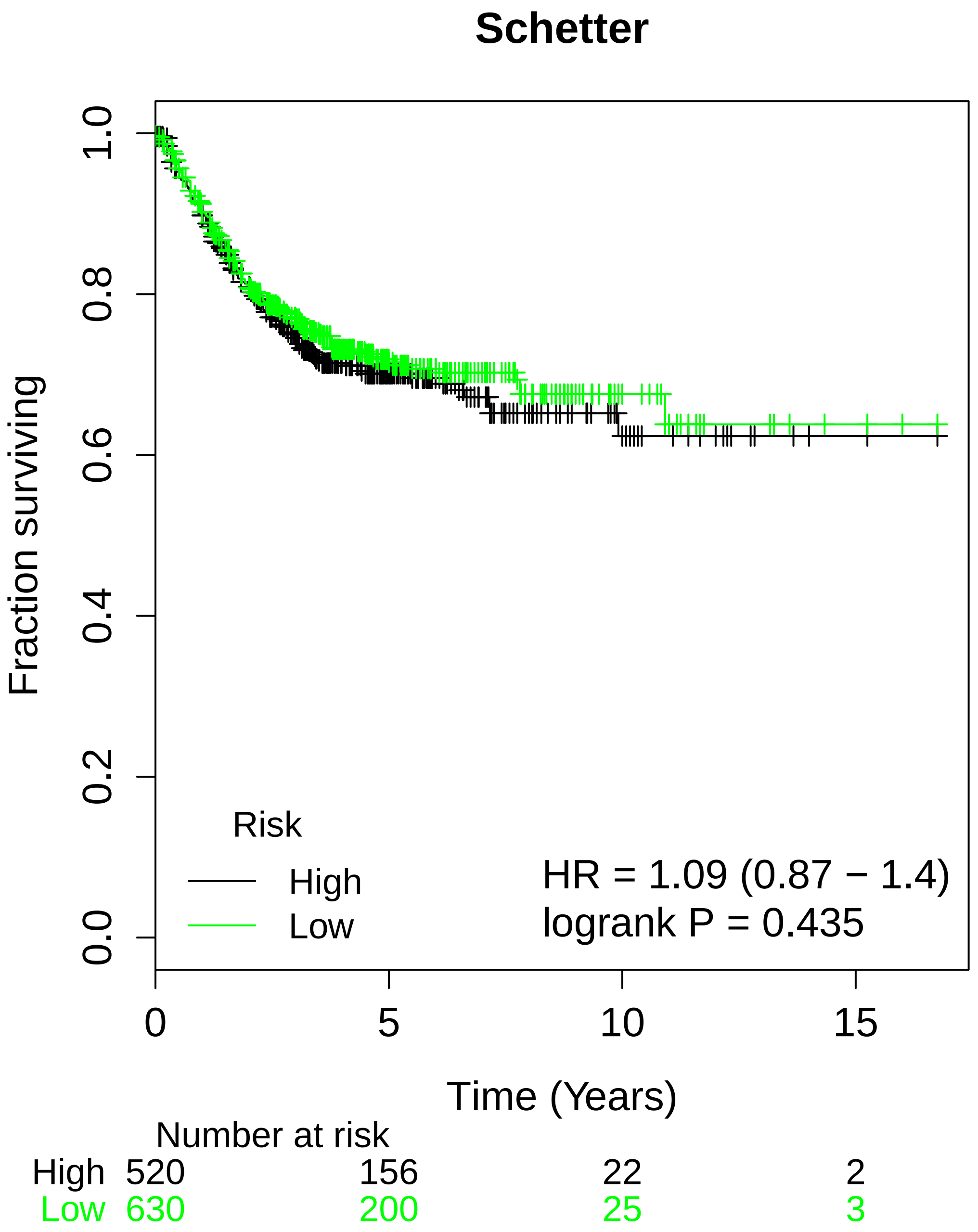
Schetter
Fraction surviving
1.0
0.8
0.6
0.4
0.2
0.0
Risk
High
Low
HR = 1.09 (0.87 − 1.4)
logrank P = 0.435
0
5
10
15
Time (Years)
Number at risk
High 520 156 22 2
Low 630 200 25 3

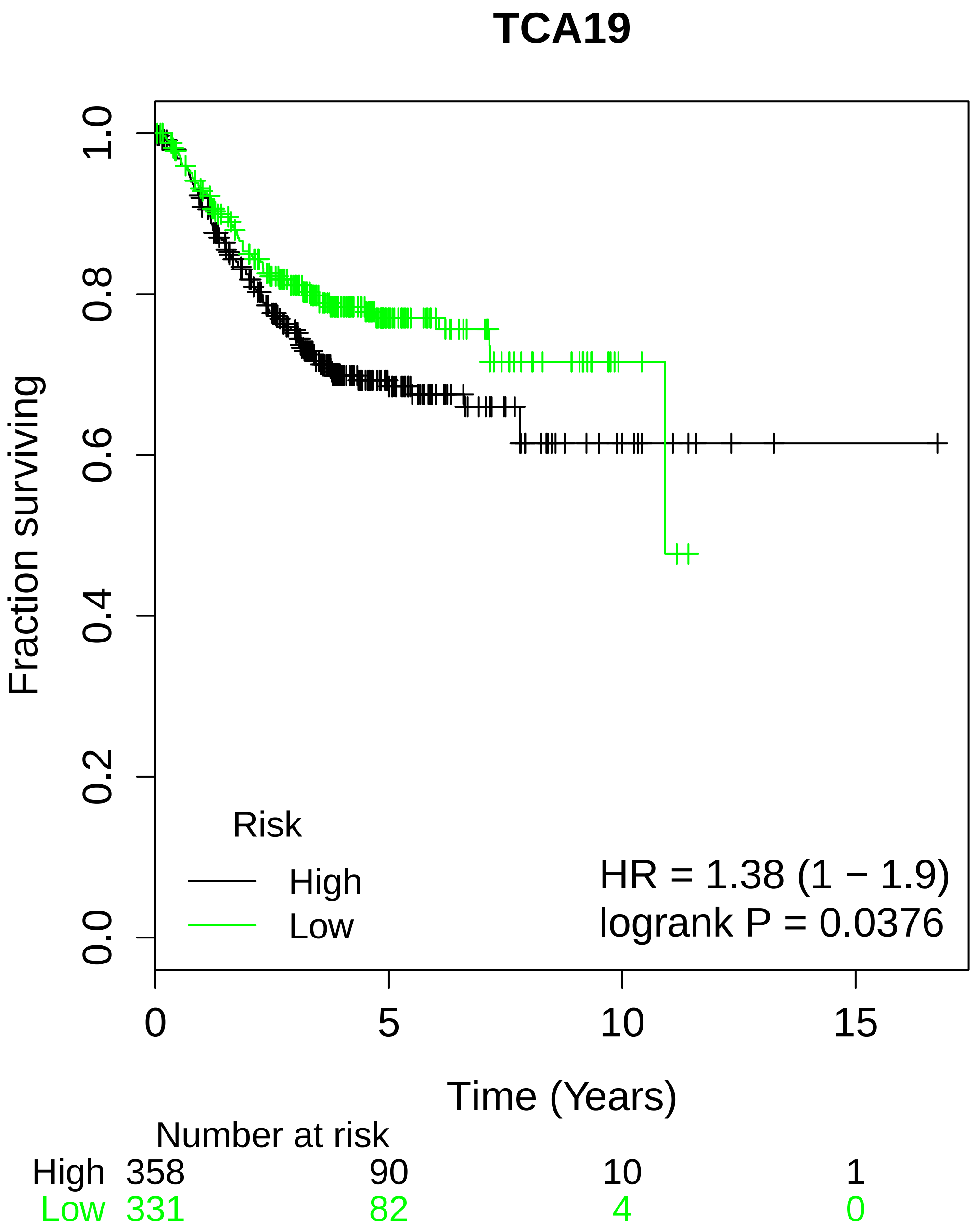

TCA19
Fraction surviving
1.0
0.8
0.6
0.4
0.2
0.0
Risk
High
Low
HR = 1.38 (1 − 1.9)
logrank P = 0.0376
0
5
10
15
Time (Years)
Number at risk
High 358 90 10 1
Low 331 82 4 0

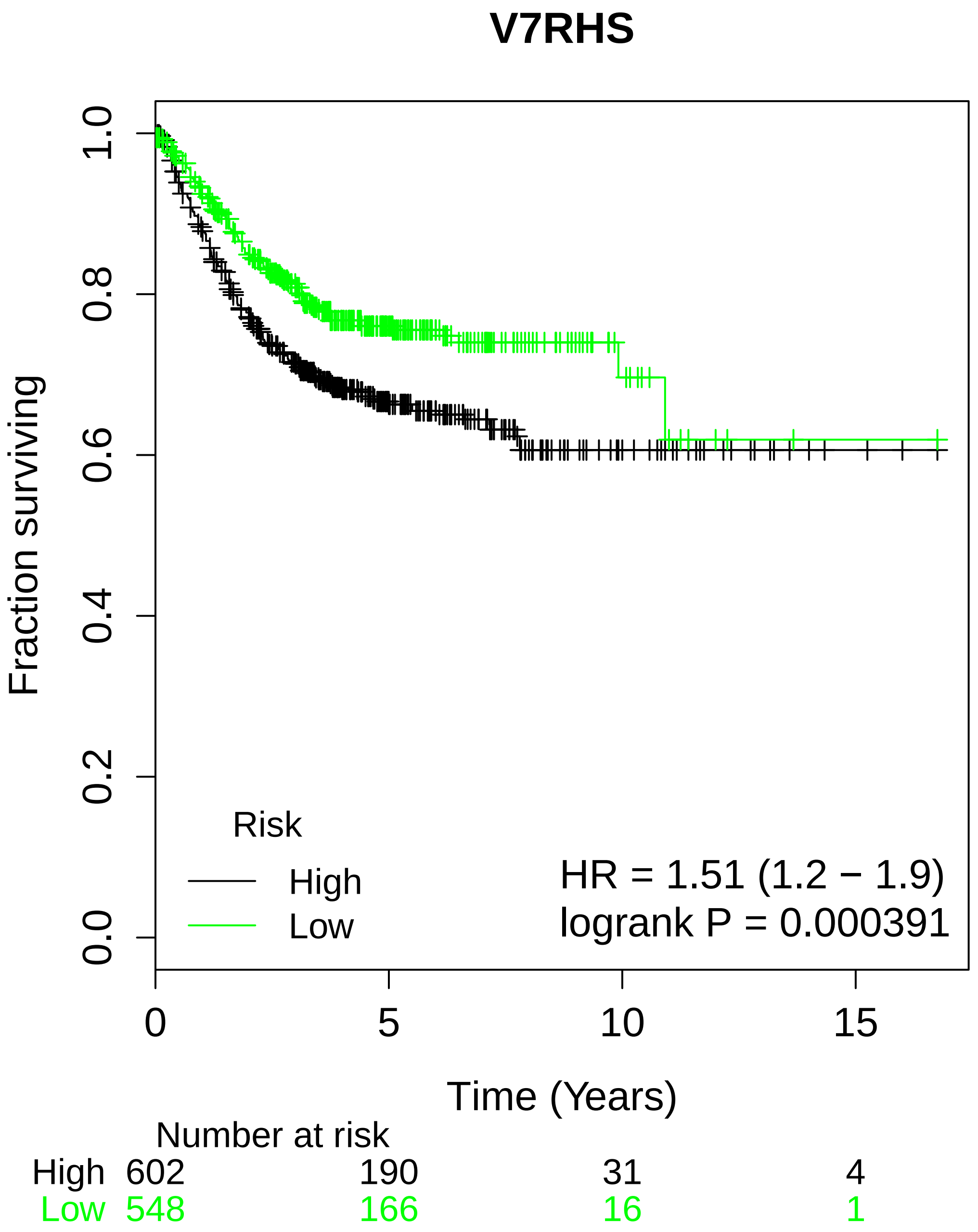

V7RHS
Fraction surviving
1.0
0.8
0.6
0.4
0.2
0.0
Risk
High
Low
HR = 1.51 (1.2 − 1.9)
logrank P = 0.000391
0
5
10
15
Time (Years)
Number at risk
High 602 190 31 4
Low 548 166 16 1

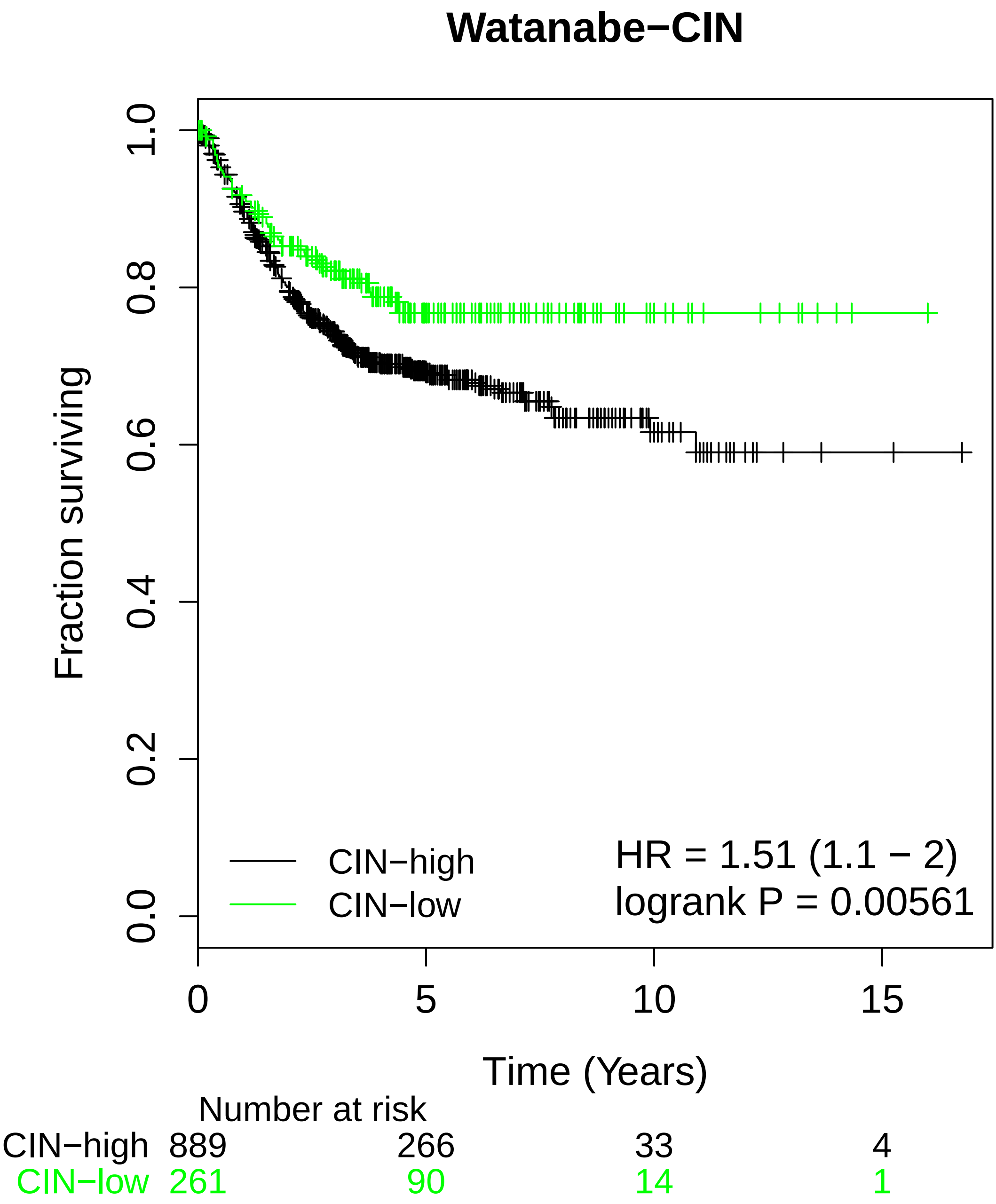
Watanabe−CIN
Fraction surviving
1.0
0.8
0.6
0.4
0.2
0.0
0
5
10
15
Time (Years)
CIN−high
CIN−low
HR = 1.51 (1.1 − 2)
logrank P = 0.00561
Number at risk
CIN−high 889 266 33 4
CIN−low 261 90 14 1

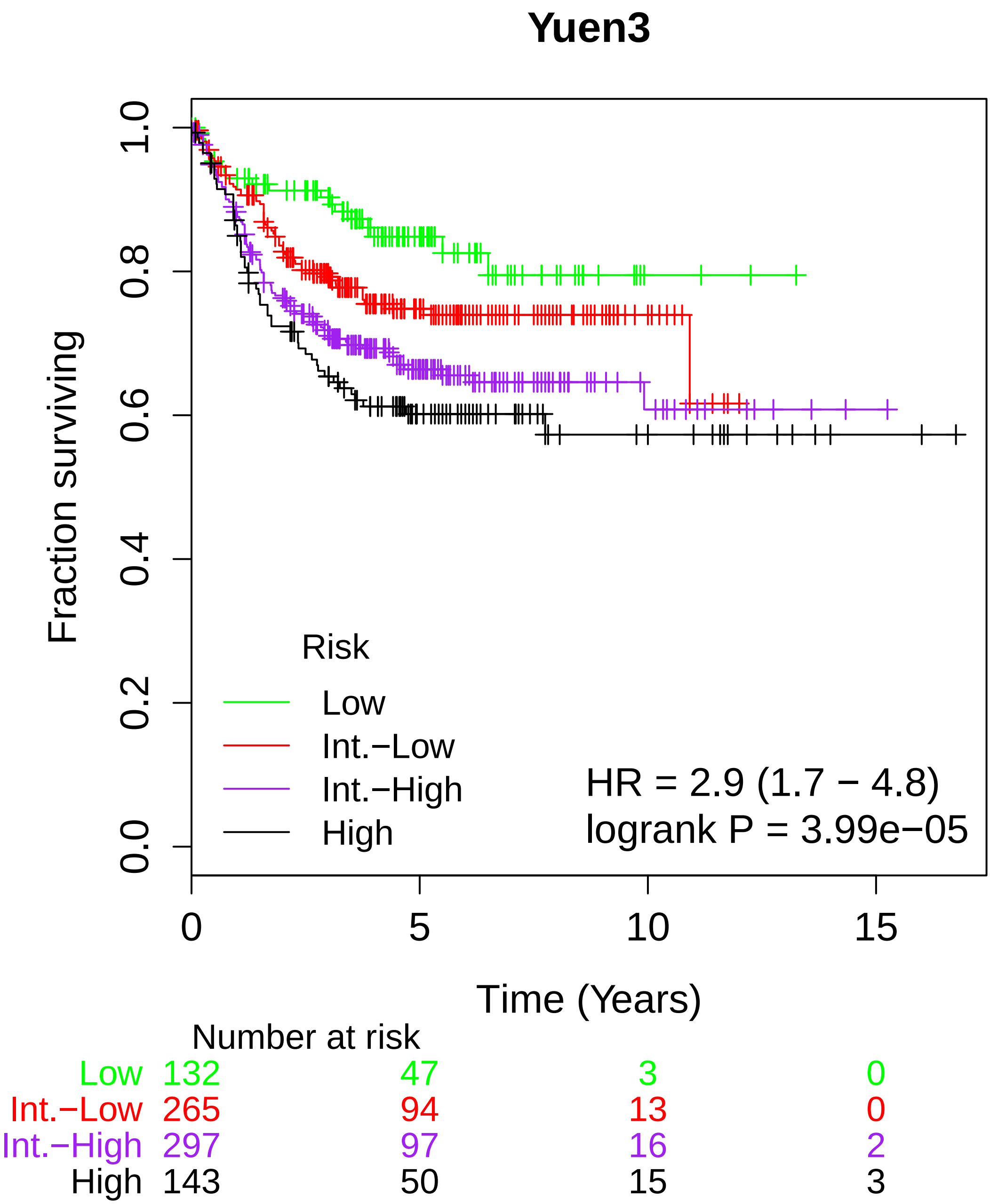

Yuen3
Fraction surviving
1.0
0.8
0.6
0.4
0.2
0.0
Risk
Low
Int.−Low
Int.−High
High
HR = 2.9 (1.7 − 4.8)
logrank P = 3.99e−05
0
5
10
15
Time (Years)
Number at risk
Low 132 47 3 0
Int.−Low 265 94 13 0
Int.−High 297 97 16 2
High 143 50 15 3

Supplementary Figure 2. Forest plot for all classifiers in all samples.

Method	HR	p−value	classified	available	%classified
Budinska	1.97	8.88e−03	313	1376	23
CCHS	1.22	6.84e−02	1376	1376	100
Chang95	2.45	1.00e−11	910	1376	66
CIN25	1.81	1.34e−08	1376	1376	100
CMS	2.20	1.06e−08	748	1376	54
ColoGuideEx	1.65	1.55e−04	1376	1376	100
ColoGuidePro	1.37	2.09e−02	1376	1376	100
CRCassigner−786	1.60	8.20e−03	440	1376	32
DeSousa	2.00	7.25e−11	1111	1376	81
Marisa	3.20	1.22e−09	329	1376	24
MDA114	1.62	5.21e−06	1376	1376	100
Merlos−EphB2−ISC	1.34	1.38e−01	412	1376	30
Merlos−Lgr5−ISC	1.40	4.11e−02	554	1376	40
Meta163	1.80	1.29e−07	1376	1376	100
ODXcolon	2.28	4.27e−12	1110	1376	81
Oncodefender	1.30	1.40e−02	1376	1376	100
Popovici	1.74	6.44e−04	1376	1376	100
Schetter	1.20	1.36e−01	1376	1376	100
TCA19	1.34	4.65e−03	1376	1376	100
V7RHS	1.41	1.07e−03	1376	1376	100
Watanabe−CIN	1.76	4.44e−05	1376	1376	100
Yuen3	2.70	5.48e−07	449	1376	33

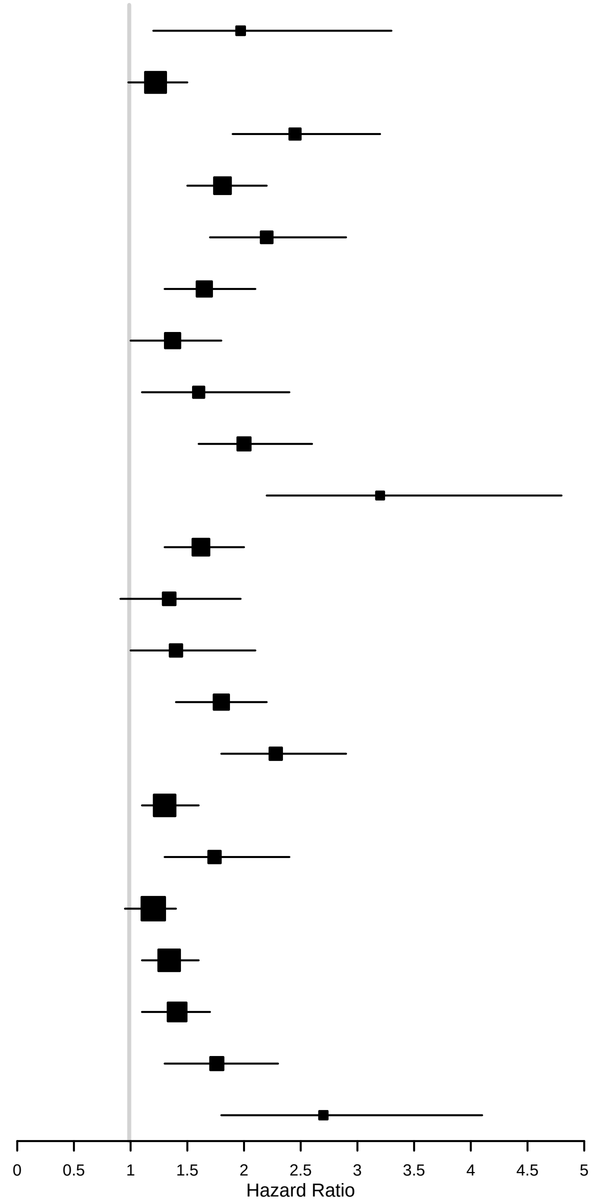

Supplementary Figure 3. Forest plot for all classifiers in stage II patients

Method	HR	p−value	classified	available	%classified
Budinska	1.90	1.86e−01	134	602	22
CCHS	1.02	9.33e−01	603	603	100
Chang95	3.11	3.80e−06	444	665	67
CIN25	1.80	1.45e−03	665	665	100
CMS*	2.20	1.46e−03	359	665	54
ColoGuideEx	1.77	1.43e−02	665	665	100
ColoGuidePro	1.35	3.64e−01	665	665	100
CRCassigner−786	1.71	1.07e−01	171	571	30
DeSousa	1.60	3.24e−02	447	575	78
Marisa	3.30	2.47e−03	141	665	21
MDA114	1.62	1.28e−02	614	614	100
Merlos−EphB2−ISC	1.46	2.65e−01	203	665	31
Merlos−Lgr5−ISC	1.05	8.73e−01	279	665	42
Meta163	1.19	4.05e−01	571	571	100
ODXcolon	2.61	5.62e−06	521	665	78
Oncodefender	1.44	5.67e−02	665	665	100
Popovici	2.06	4.79e−03	665	665	100
Schetter	1.21	3.06e−01	665	665	100
TCA19	1.58	8.20e−02	403	403	100
V7RHS	1.60	1.25e−02	665	665	100
Watanabe−CIN	1.58	5.44e−02	665	665	100
Yuen3	2.50	1.50e−02	163	509	32

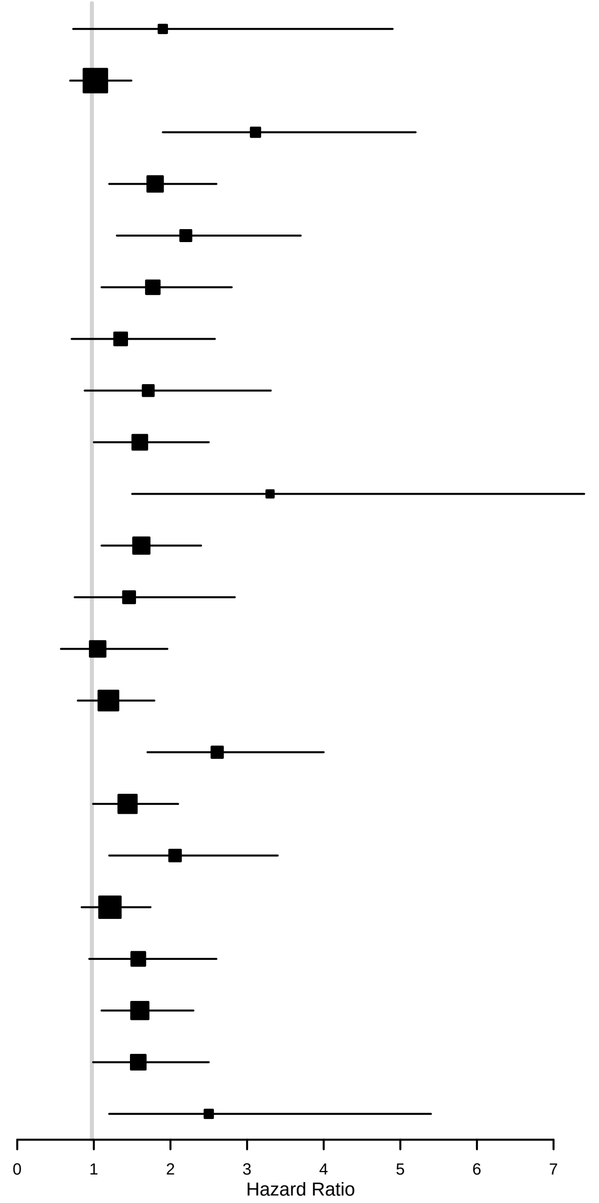

Supplementary Figure 4. Forest plot for all classifiers in stage III patients

Method	HR	p−value	classified	available	%classified
Budinska	2.22	0.03670	113	414	27
CCHS	1.08	0.66600	422	422	100
Chang95	1.75	0.00289	314	488	64
CIN25	1.33	0.04990	488	488	100
CMS*	1.70	0.00814	265	488	54
ColoGuideEx	1.45	0.04940	488	488	100
ColoGuidePro	1.37	0.08100	488	488	100
CRCassigner−786	1.80	0.03510	146	397	37
DeSousa	1.50	0.00848	403	488	83
Marisa	1.73	0.05160	115	488	24
MDA114	1.28	0.11900	440	440	100
Merlos−EphB2−ISC	1.12	0.69100	142	488	29
Merlos−Lgr5−ISC	1.25	0.39600	189	488	39
Meta163	1.38	0.05750	397	397	100
ODXcolon	1.52	0.01400	394	488	81
Oncodefender	1.05	0.73900	488	488	100
Popovici	1.41	0.15700	488	488	100
Schetter	1.29	0.07970	488	488	100
TCA19	1.28	0.20500	286	286	100
V7RHS	1.43	0.01510	488	488	100
Watanabe−CIN	1.40	0.10200	488	488	100
Yuen3	2.30	0.01960	113	331	34

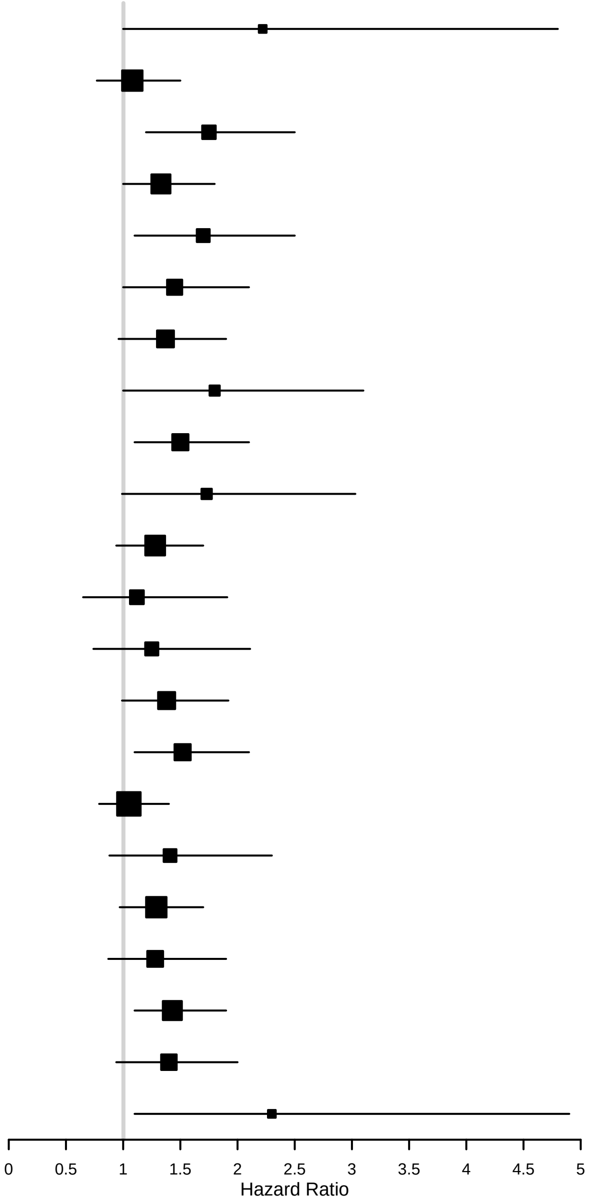

Supplementary Figure 5.
Individual Kaplan-Meier plots for each classifier in all samples.

22 Kaplan-Meier plots

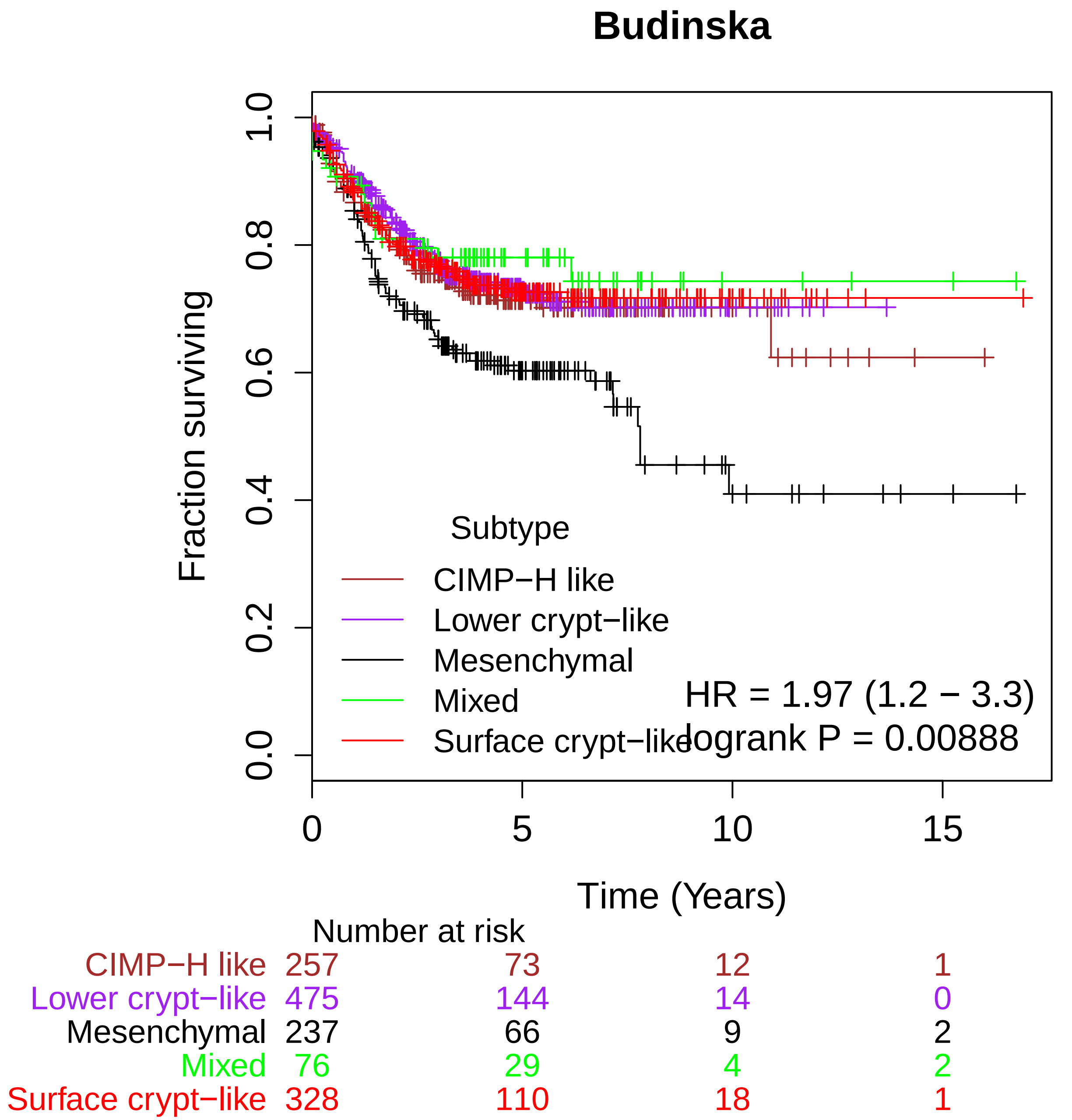
Budinska
Fraction surviving
0.0
0.2
0.4
0.6
0.8
1.0
0
5
10
15
Time (Years)
Subtype
CIMP−H like
Lower crypt−like
Mesenchymal
Mixed
Surface crypt−like
HR = 1.97 (1.2 − 3.3)
logrank P = 0.00888
Number at risk
CIMP−H like 257 73 12 1
Lower crypt−like 475 144 14 0
Mesenchymal 237 66 9 2
Mixed 76 29 4 2
Surface crypt−like 328 110 18 1

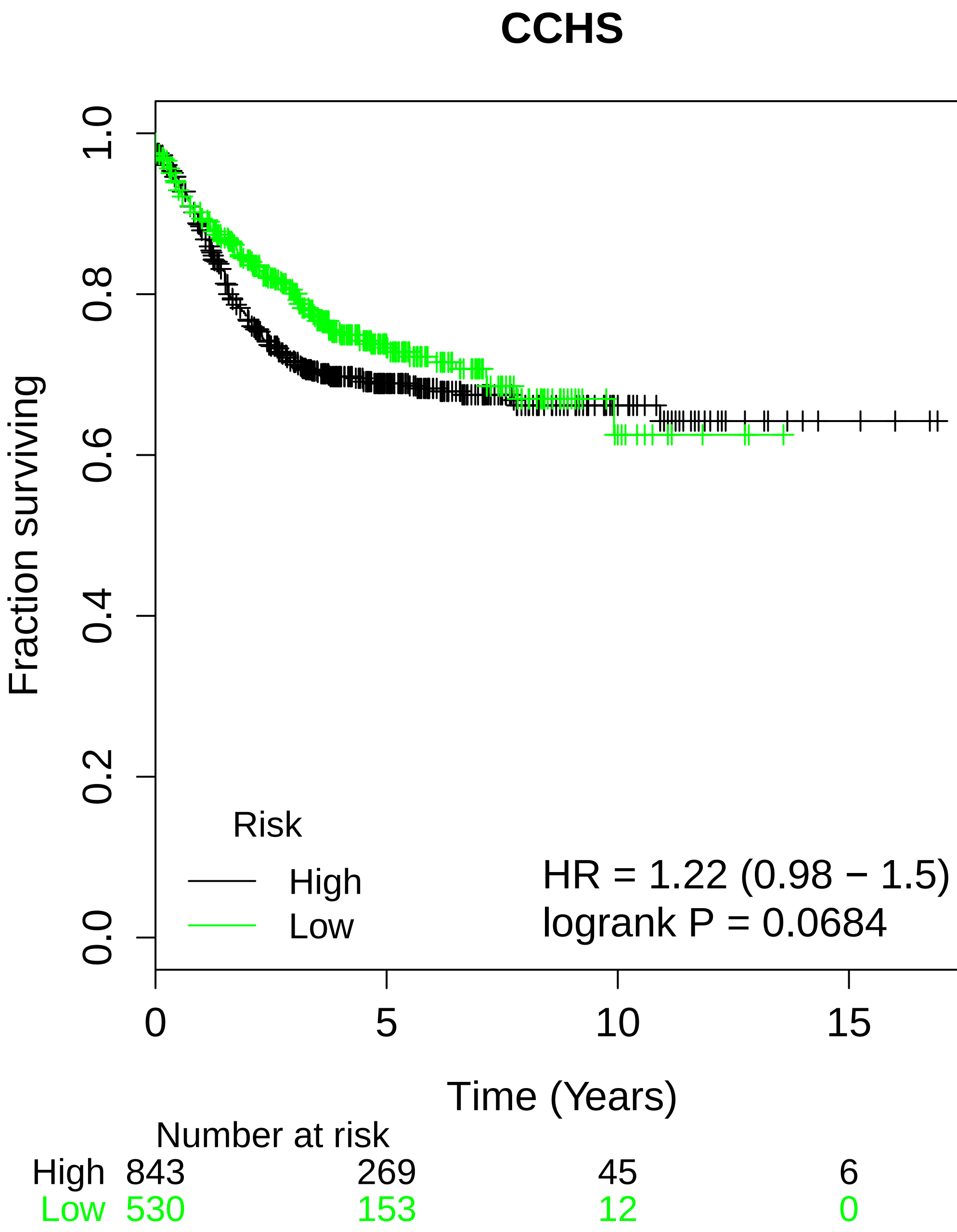

CCHS
Fraction surviving
1.0
0.8
0.6
0.4
0.2
0.0
Risk
High
Low
HR = 1.22 (0.98 − 1.5)
logrank P = 0.0684
0
5
10
15
Time (Years)
Number at risk
High 843 269 45 6
Low 530 153 12 0

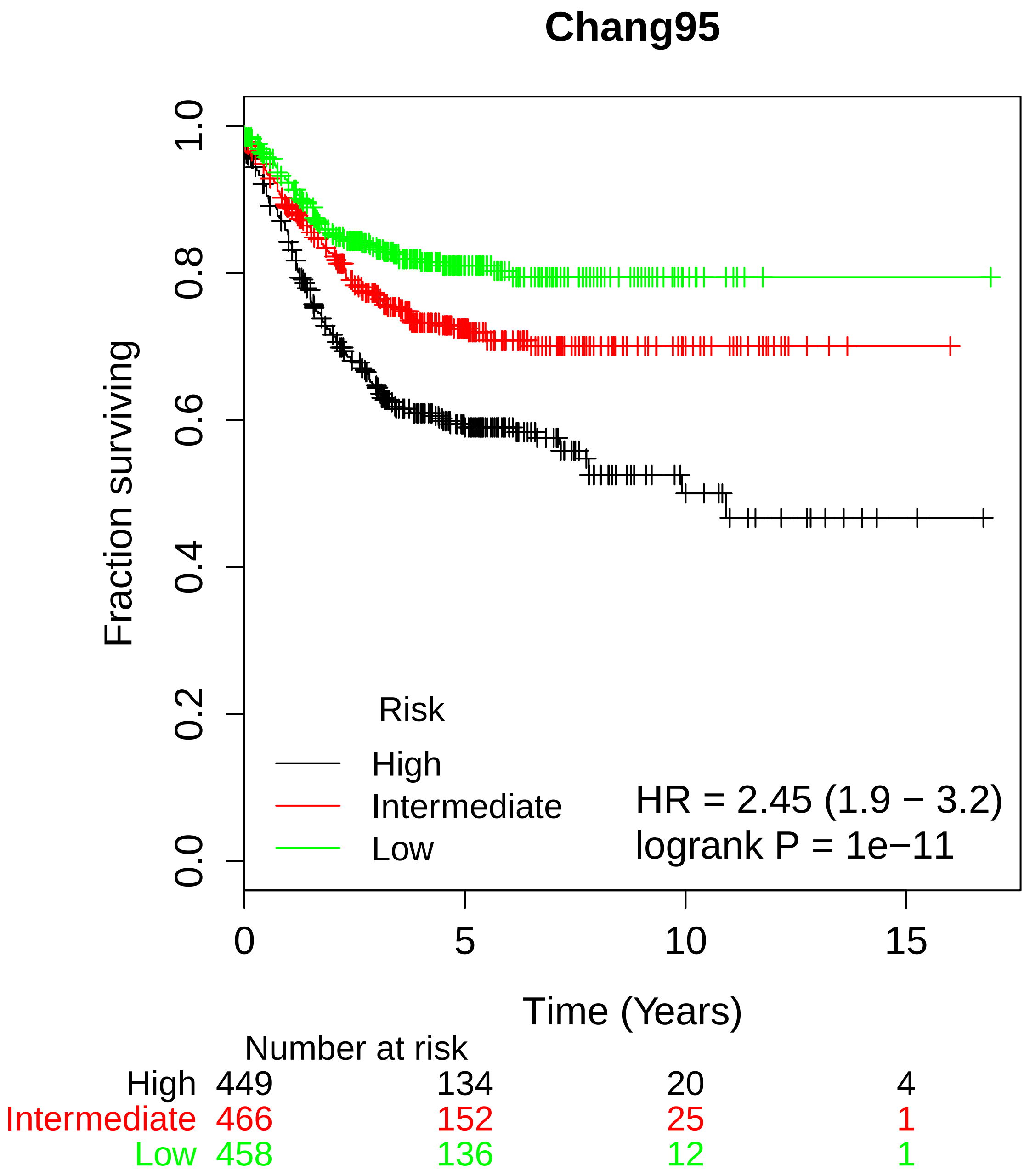

Chang95
Fraction surviving
1.0
0.8
0.6
0.4
0.2
0.0
0
5
10
15
Time (Years)
Risk
High
Intermediate
Low
HR = 2.45 (1.9 − 3.2)
logrank P = 1e−11
Number at risk
High 449 134 20 4
Intermediate 466 152 25 1
Low 458 136 12 1

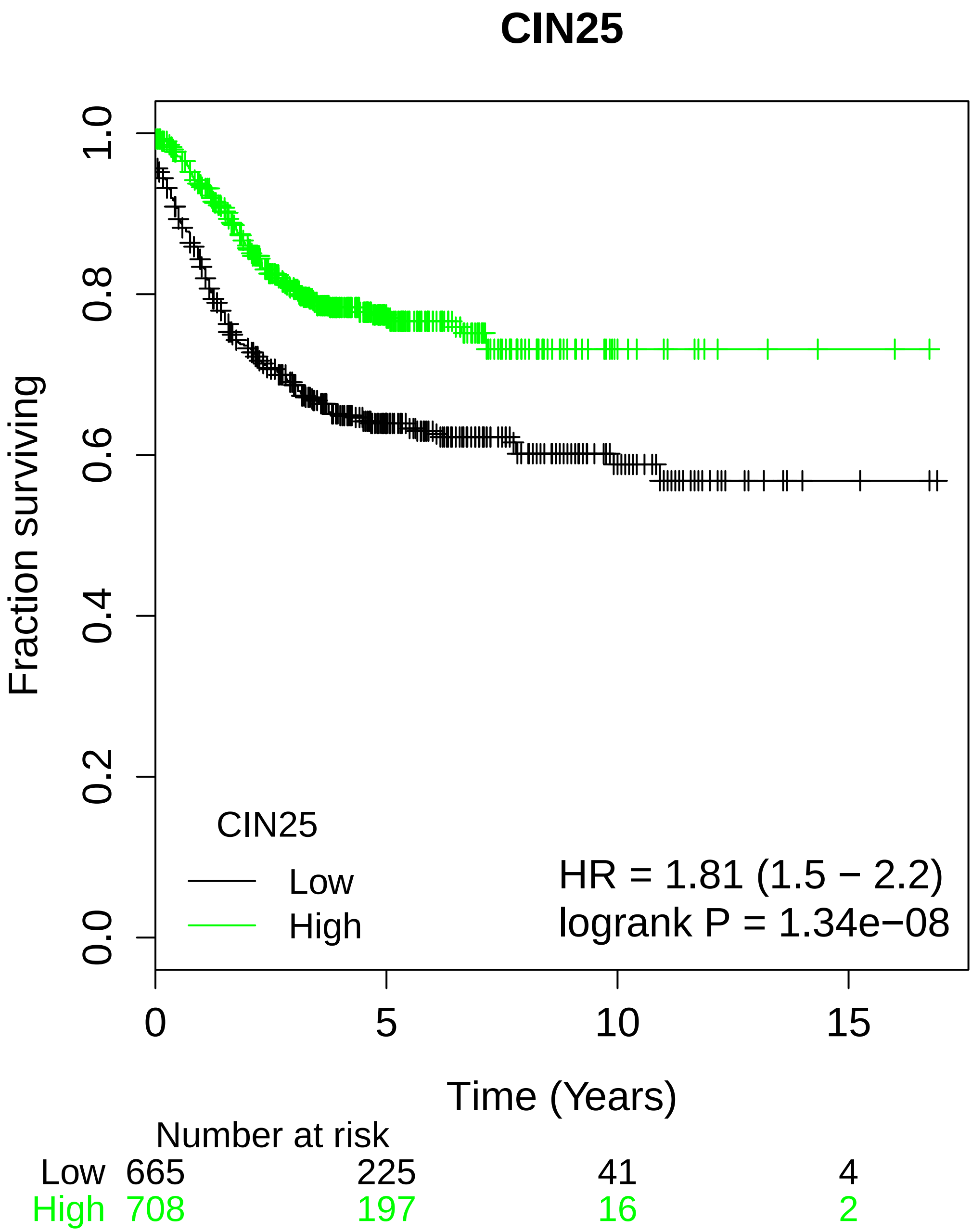
CIN25
1.0
0.8
0.6
0.4
0.2
0.0
Fraction surviving
CIN25
Low
High
HR = 1.81 (1.5 − 2.2)
logrank P = 1.34e−08
0
5
10
15
Time (Years)
Number at risk
Low 665 225 41 4
High 708 197 16 2

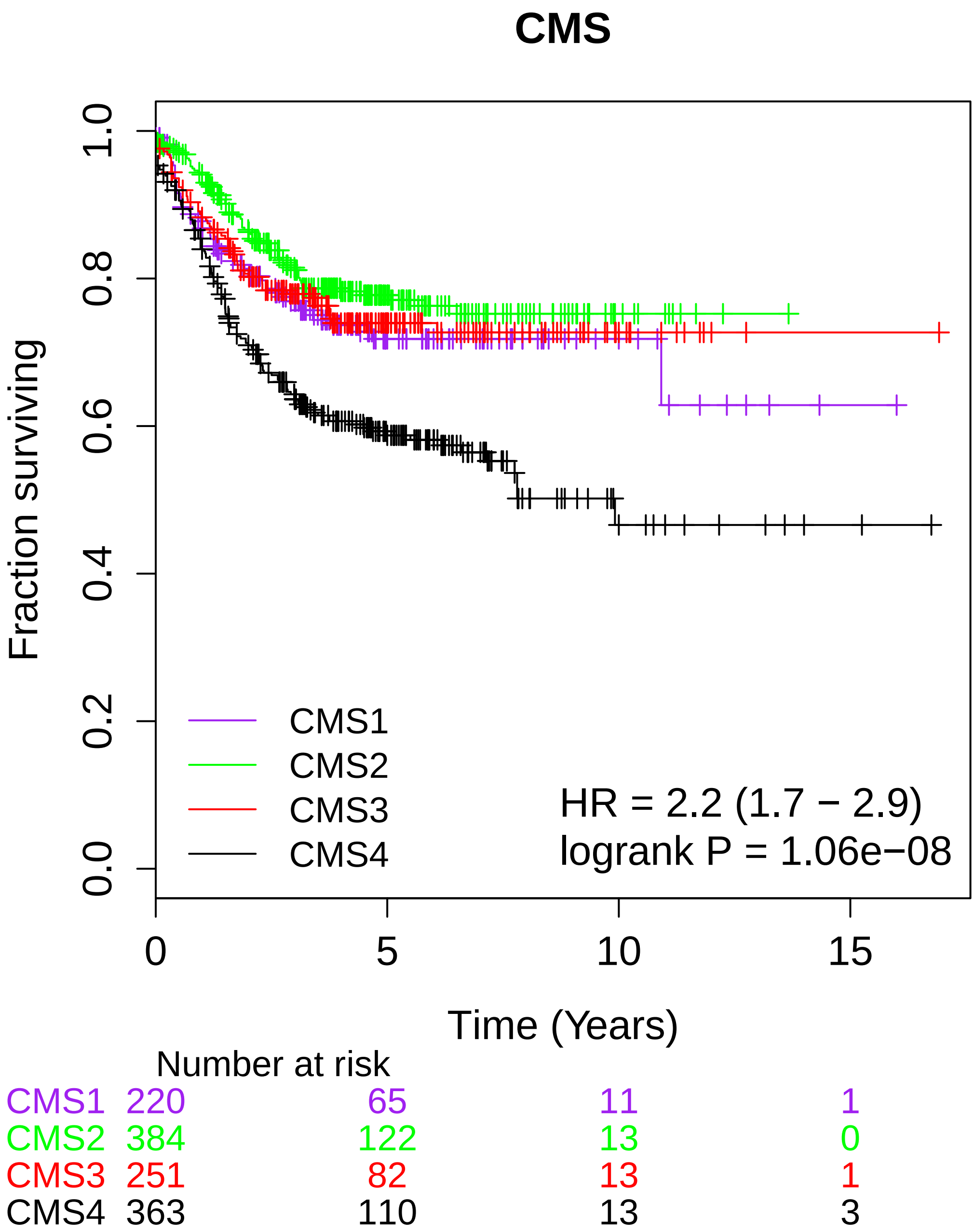
CMS
Fraction surviving
1.0
0.8
0.6
0.4
0.2
0.0
CMS1
CMS2
CMS3
CMS4
HR = 2.2 (1.7 − 2.9)
logrank P = 1.06e−08
0
5
10
15
Time (Years)
Number at risk
CMS1 220 65 11 1
CMS2 384 122 13 0
CMS3 251 82 13 1
CMS4 363 110 13 3

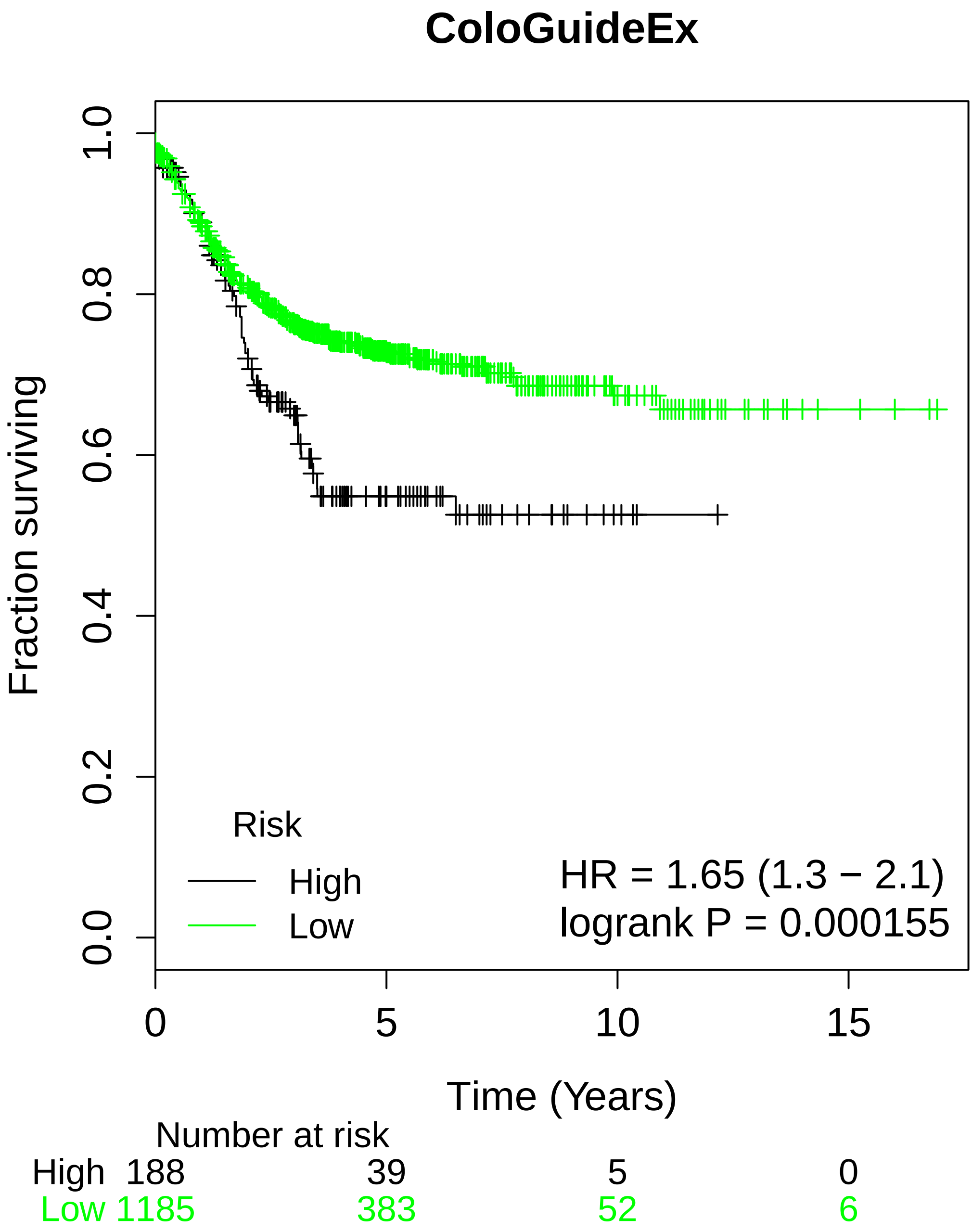

ColoGuideEx
Fraction surviving
1.0
0.8
0.6
0.4
0.2
0.0
Risk
High
Low
HR = 1.65 (1.3 − 2.1)
logrank P = 0.000155
0
5
10
15
Time (Years)
Number at risk
High 188 39 5 0
Low 1185 383 52 6

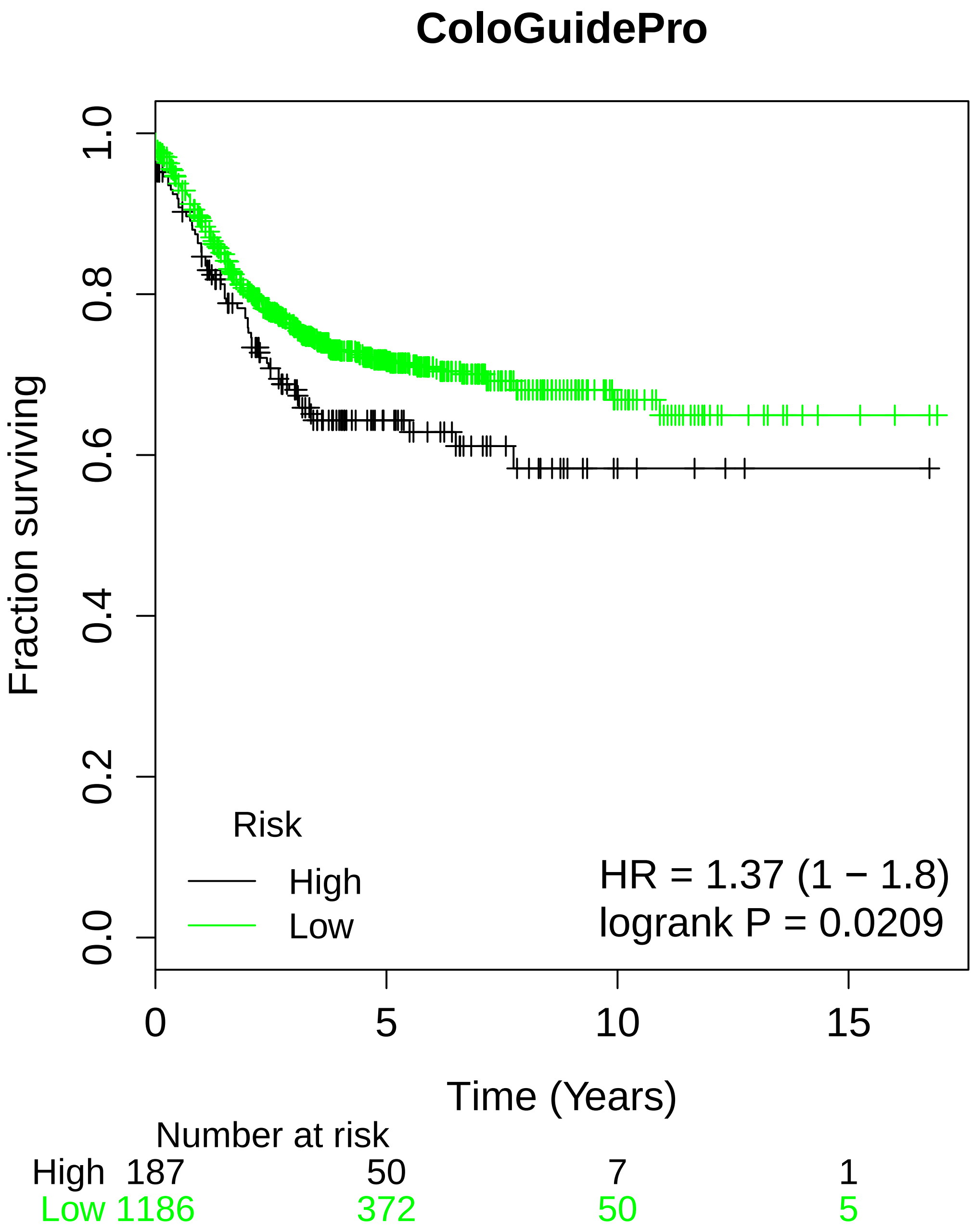
ColoGuidePro
Fraction surviving
1.0
0.8
0.6
0.4
0.2
0.0
Risk
High
Low
HR = 1.37 (1 − 1.8)
logrank P = 0.0209
0
5
10
15
Time (Years)
Number at risk
High 187 50 7 1
Low 1186 372 50 5

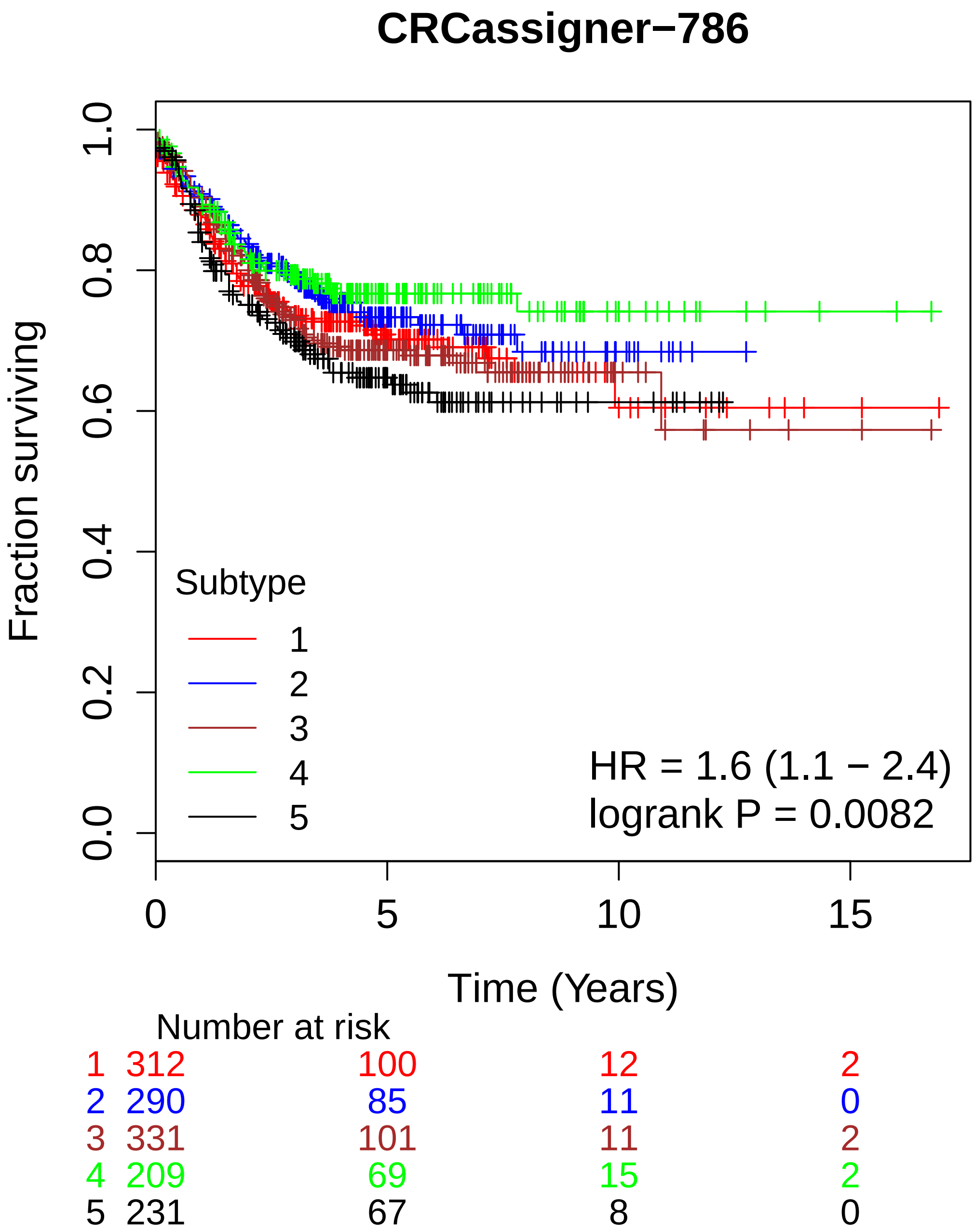
CRCassigner−786
Fraction surviving
1.0
0.8
0.6
0.4
0.2
0.0
Subtype
1
2
3
4
5
HR = 1.6 (1.1 − 2.4)
logrank P = 0.0082
0
5
10
15
Time (Years)
Number at risk
1 312 100 12 2
2 290 85 11 0
3 331 101 11 2
4 209 69 15 2
5 231 67 8 0

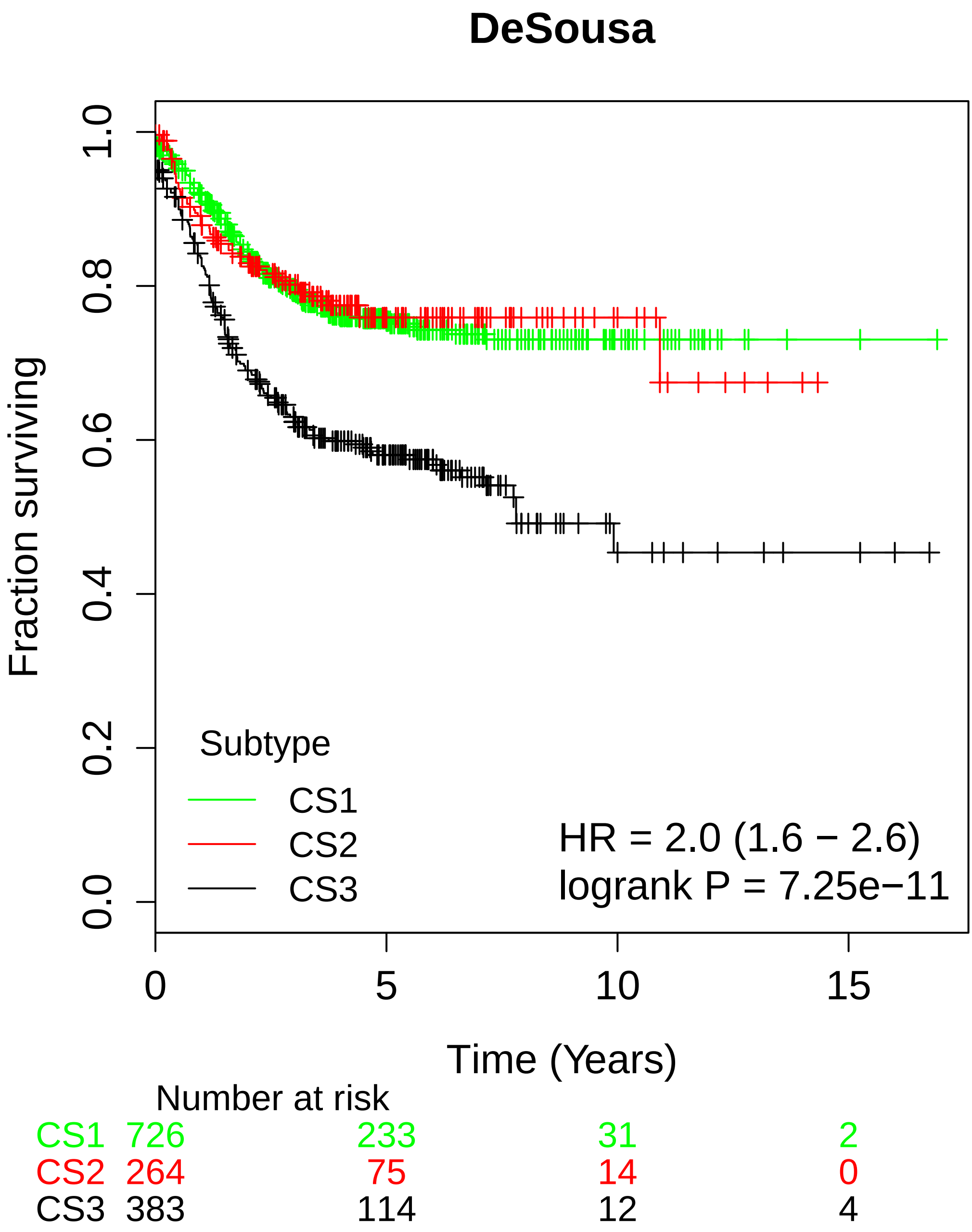

DeSousa
Fraction surviving
1.0
0.8
0.6
0.4
0.2
0.0
Subtype
CS1
CS2
CS3
HR = 2.0 (1.6 − 2.6)
logrank P = 7.25e−11
0
5
10
15
Time (Years)
Number at risk
CS1 726 233 31 2
CS2 264 75 14 0
CS3 383 114 12 4

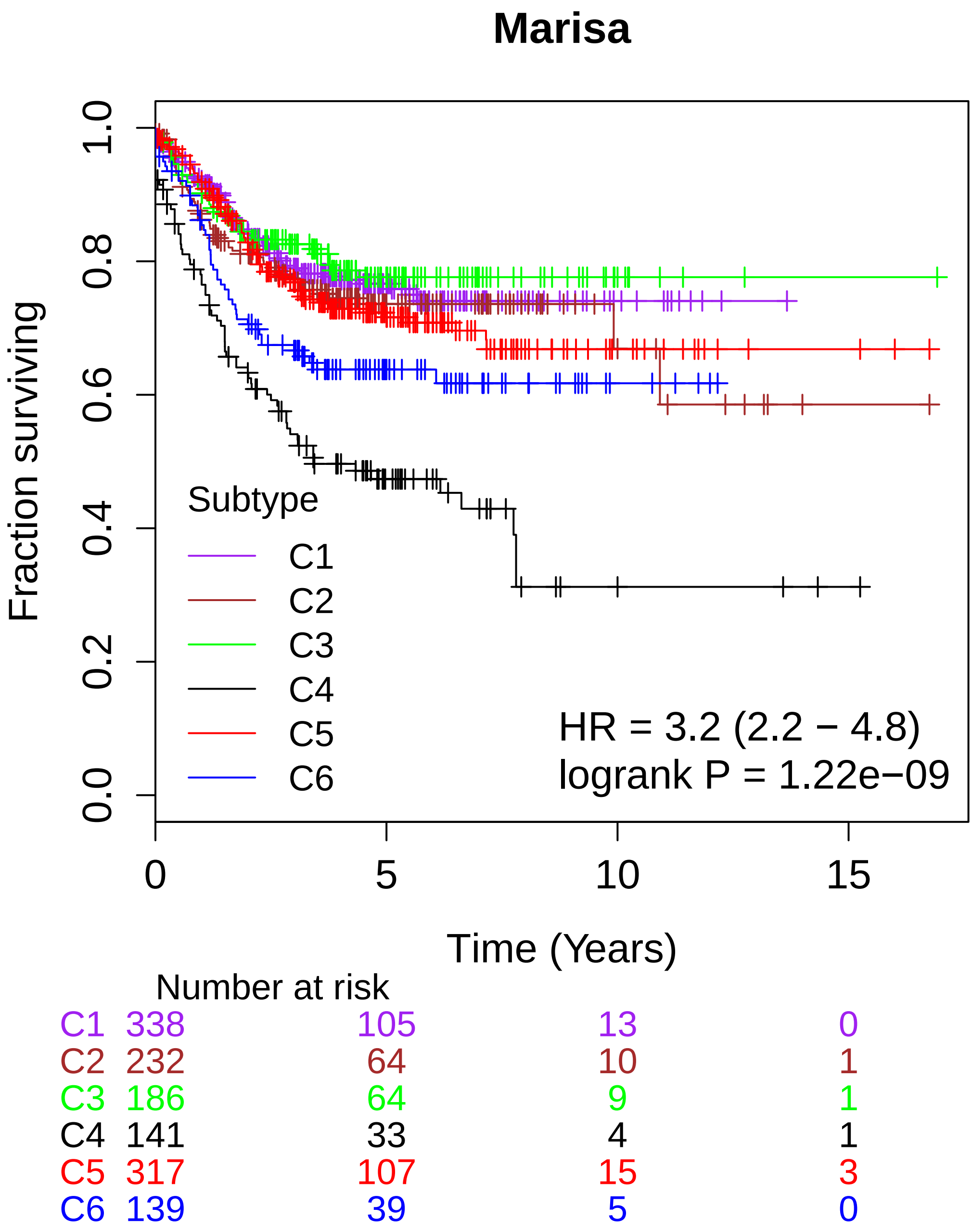
Marisa
Fraction surviving
1.0
0.8
0.6
0.4
0.2
0.0
Subtype
C1
C2
C3
C4
C5
C6
HR = 3.2 (2.2 − 4.8)
logrank P = 1.22e−09
0
5
10
15
Time (Years)
Number at risk
C1 338 105 13 0
C2 232 64 10 1
C3 186 64 9 1
C4 141 33 4 1
C5 317 107 15 3
C6 139 39 5 0

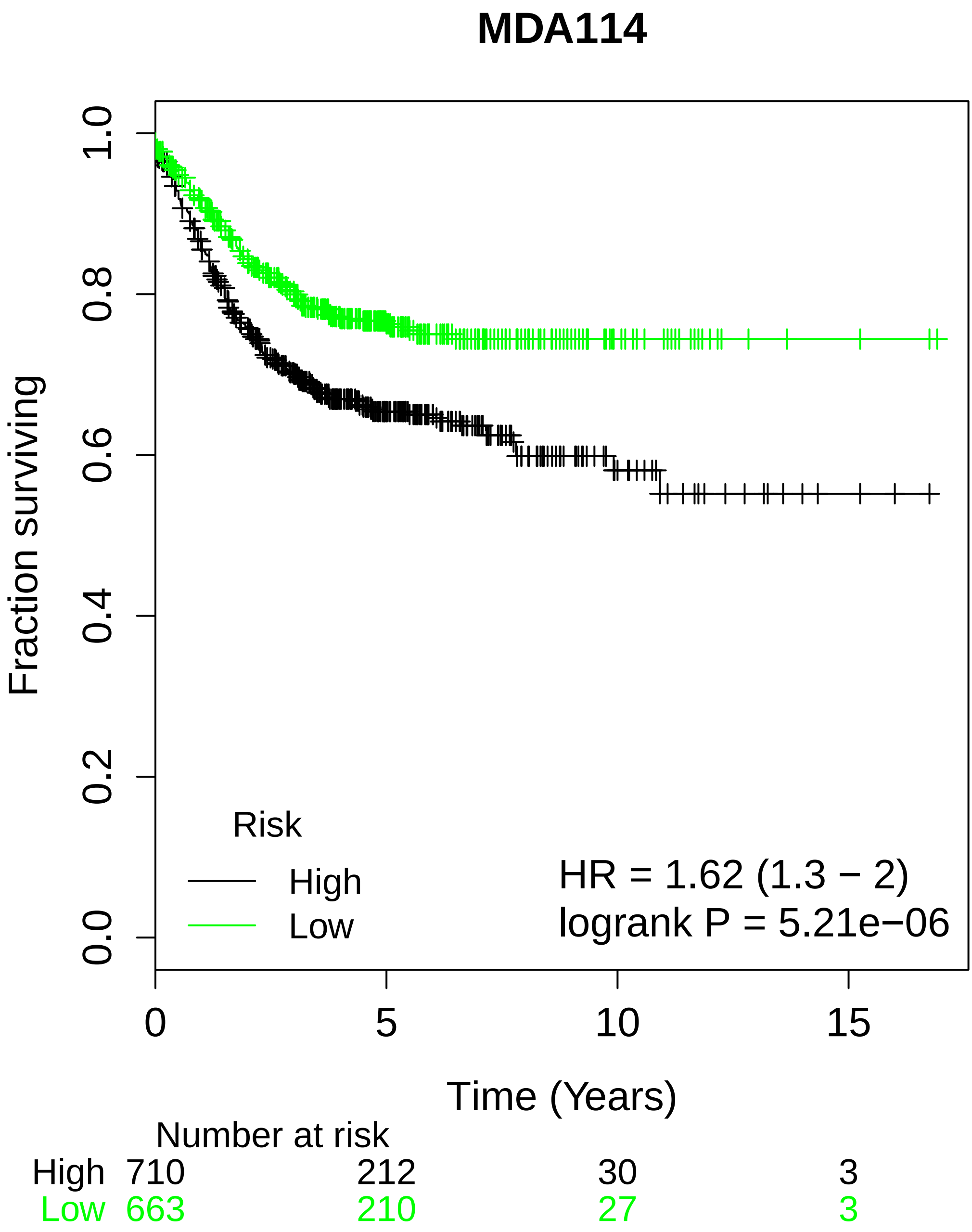
MDA114
Fraction surviving
1.0
0.8
0.6
0.4
0.2
0.0
Risk
High
Low
HR = 1.62 (1.3 − 2)
logrank P = 5.21e−06
0
5
10
15
Time (Years)
Number at risk
High 710 212 30 3
Low 663 210 27 3

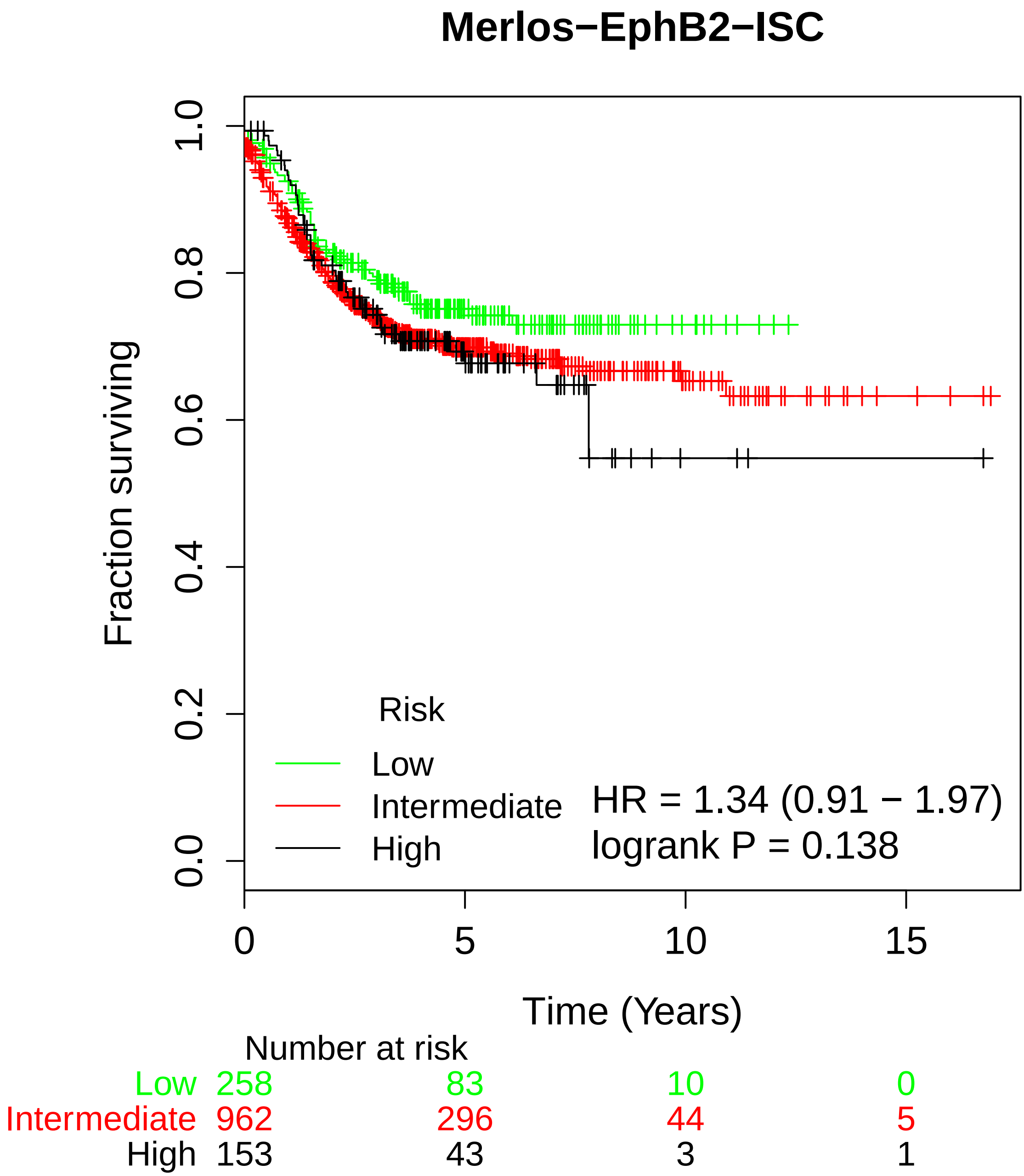

Merlos−EphB2−ISC
Fraction surviving
1.0
0.8
0.6
0.4
0.2
0.0
Risk
Low
Intermediate
High
HR = 1.34 (0.91 − 1.97)
logrank P = 0.138
0
5
10
15
Time (Years)
Number at risk
Low 258 83 10 0
Intermediate 962 296 44 5
High 153 43 3 1

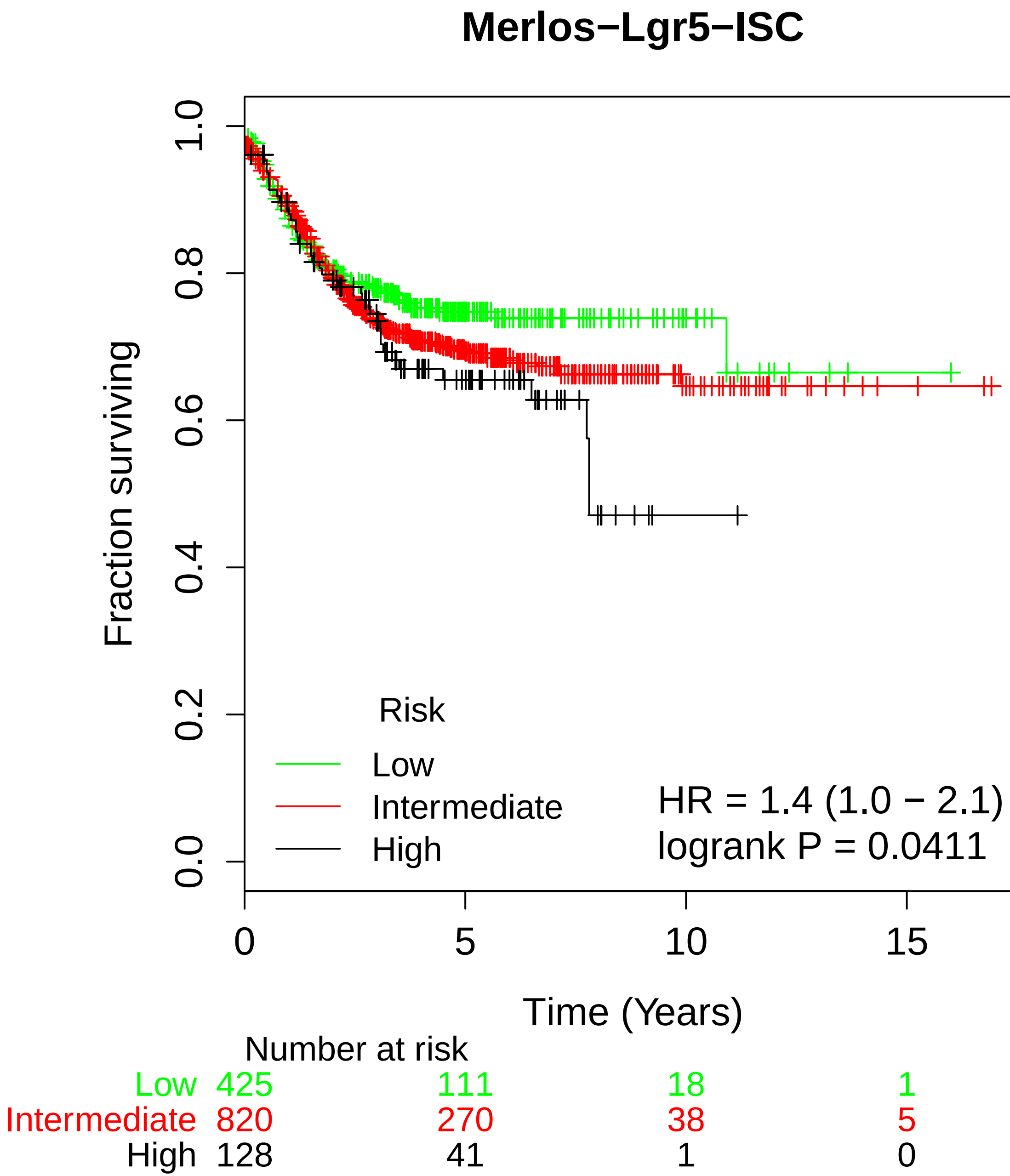
Merlos−Lgr5−ISC
Fraction surviving
1.0
0.8
0.6
0.4
0.2
0.0
0
5
10
15
Time (Years)
Risk
Low
Intermediate
High
HR = 1.4 (1.0 − 2.1)
logrank P = 0.0411
Number at risk
Low 425 111 18 1
Intermediate 820 270 38 5
High 128 41 1 0

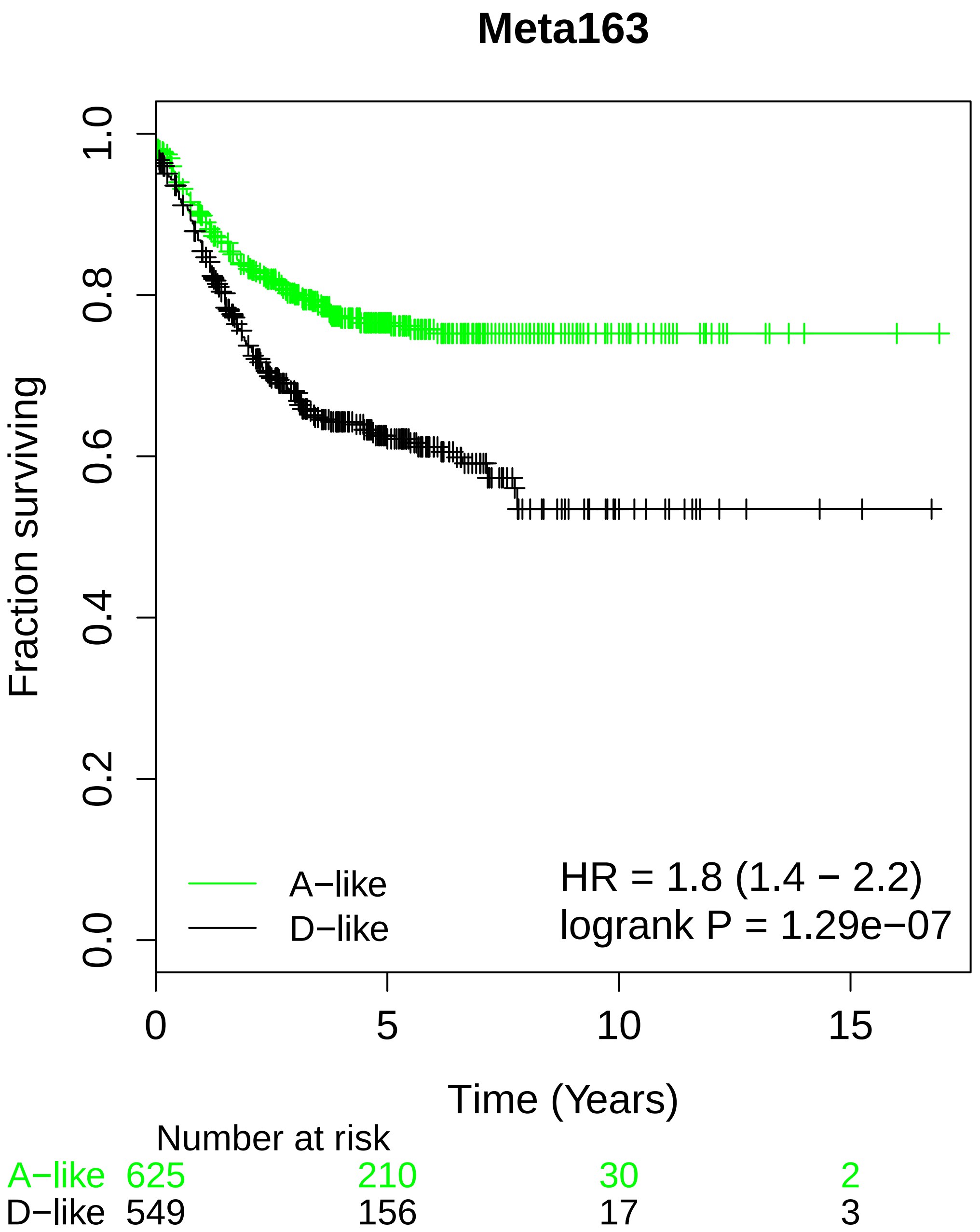
Meta163
Fraction surviving
1.0
0.8
0.6
0.4
0.2
0.0
0
5
10
15
Time (Years)
A−like
D−like
HR = 1.8 (1.4 − 2.2)
logrank P = 1.29e−07
Number at risk
A−like 625 210 30 2
D−like 549 156 17 3

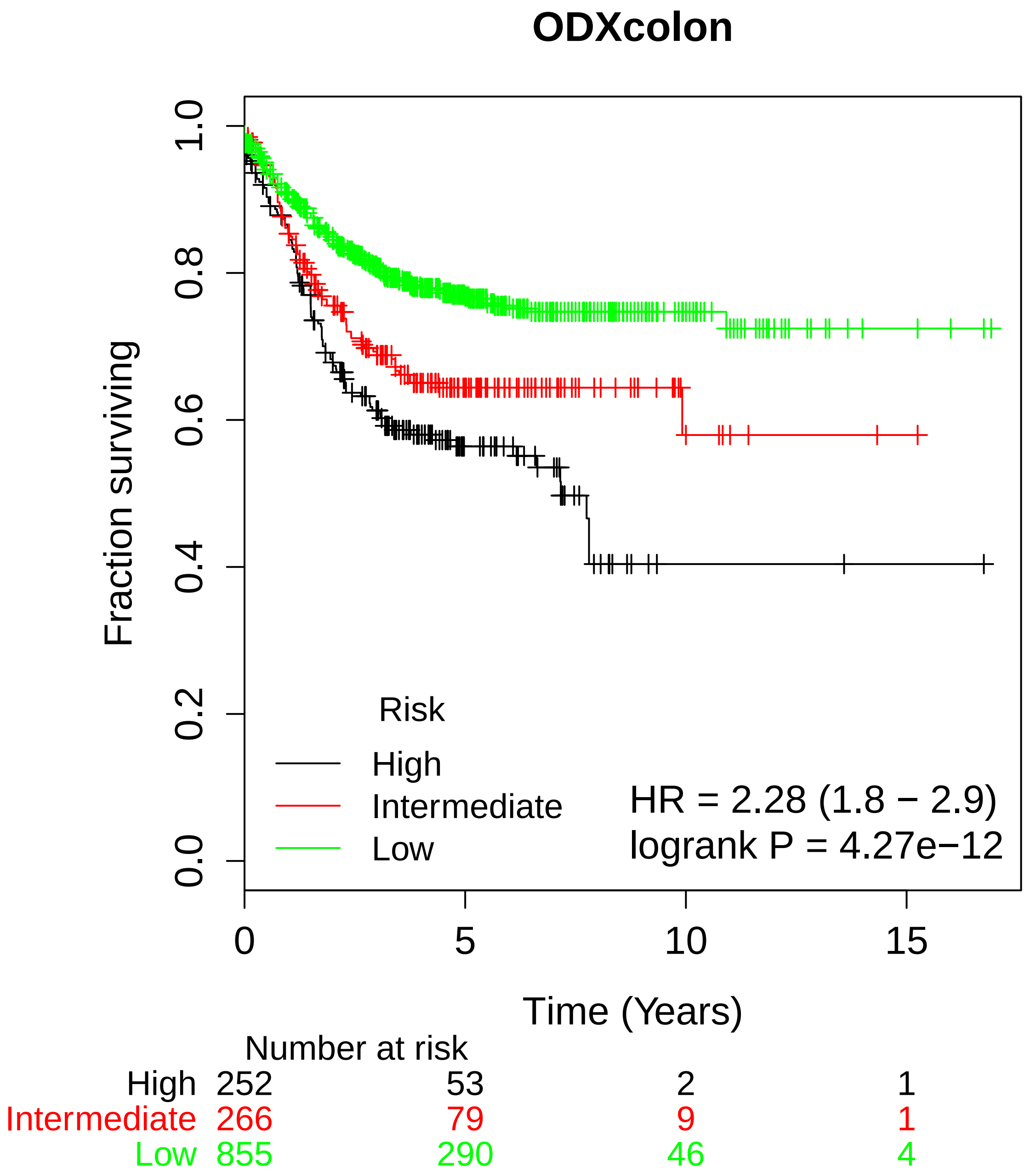

ODXcolon
Fraction surviving
1.0
0.8
0.6
0.4
0.2
0.0
0
5
10
15
Time (Years)
Risk
High
Intermediate
Low
HR = 2.28 (1.8 − 2.9)
logrank P = 4.27e−12
Number at risk
High 252 53 2 1
Intermediate 266 79 9 1
Low 855 290 46 4

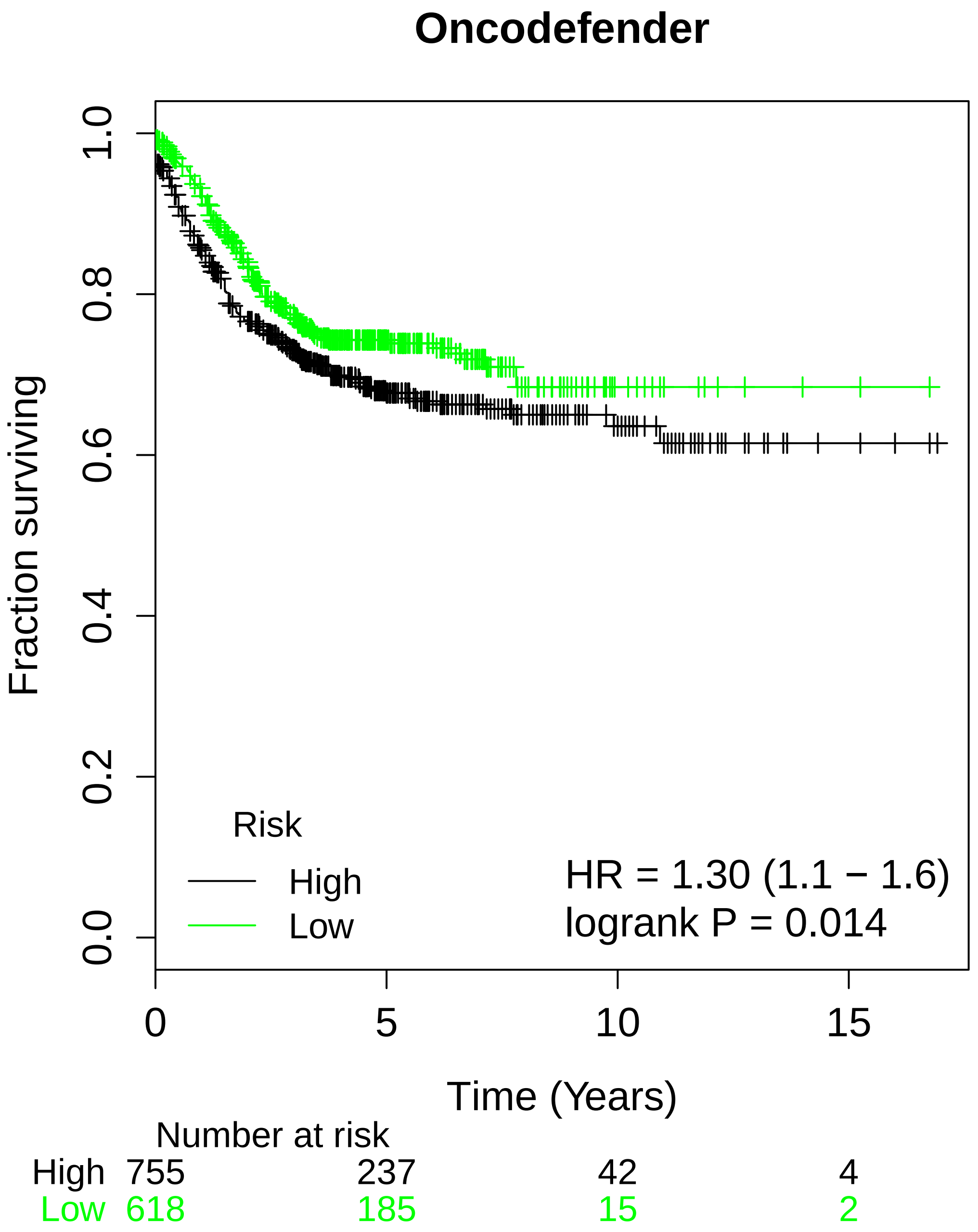
Oncodefender
Fraction surviving
1.0
0.8
0.6
0.4
0.2
0.0
0
5
10
15
Time (Years)
Risk
High
Low
HR = 1.30 (1.1 − 1.6)
logrank P = 0.014
Number at risk
High 755 237 42 4
Low 618 185 15 2

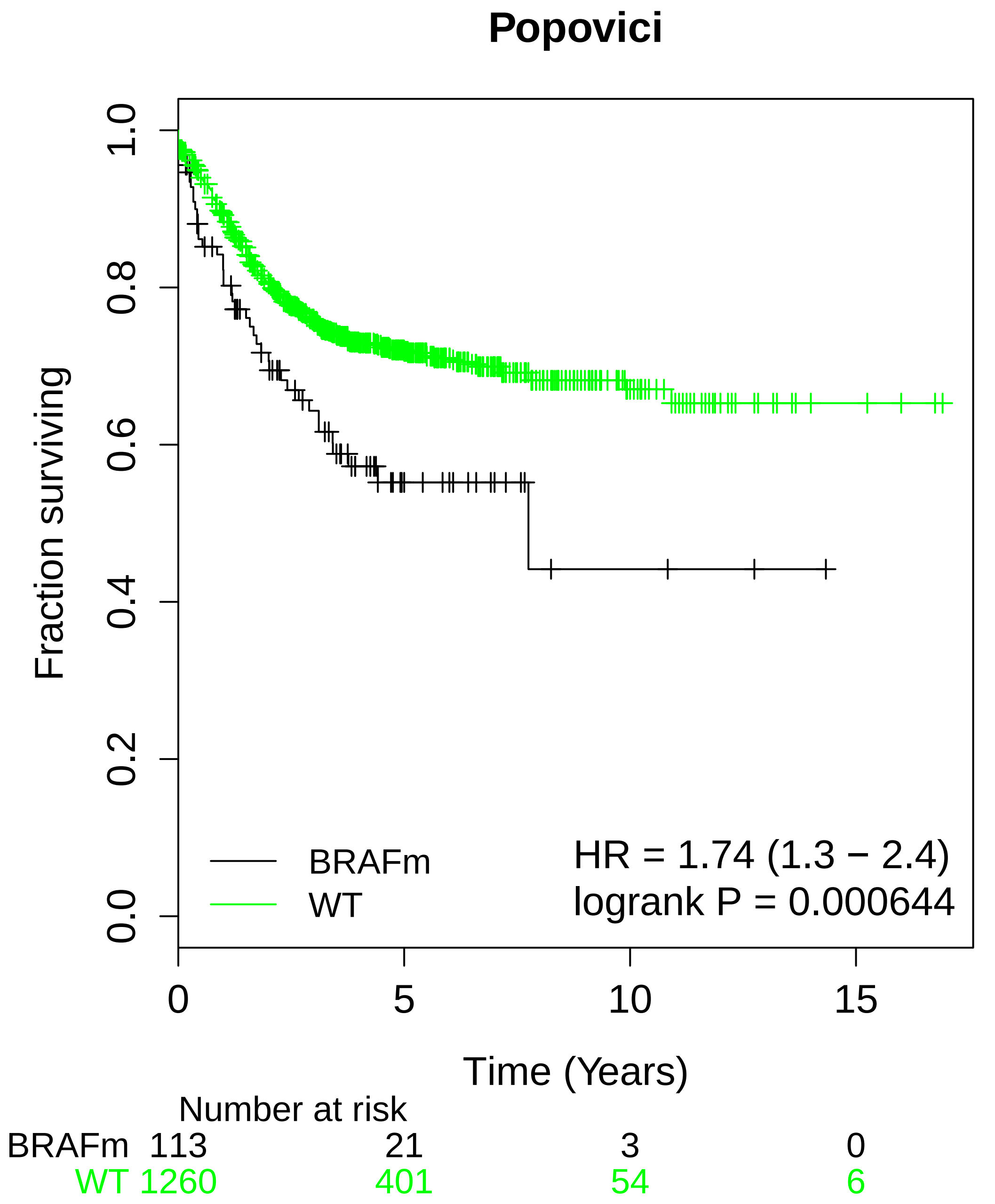

Popovici
Fraction surviving
1.0
0.8
0.6
0.4
0.2
0.0
BRAFm
WT
HR = 1.74 (1.3 − 2.4)
logrank P = 0.000644
0
5
10
15
Time (Years)
Number at risk
BRAFm 113 21 3 0
WT 1260 401 54 6

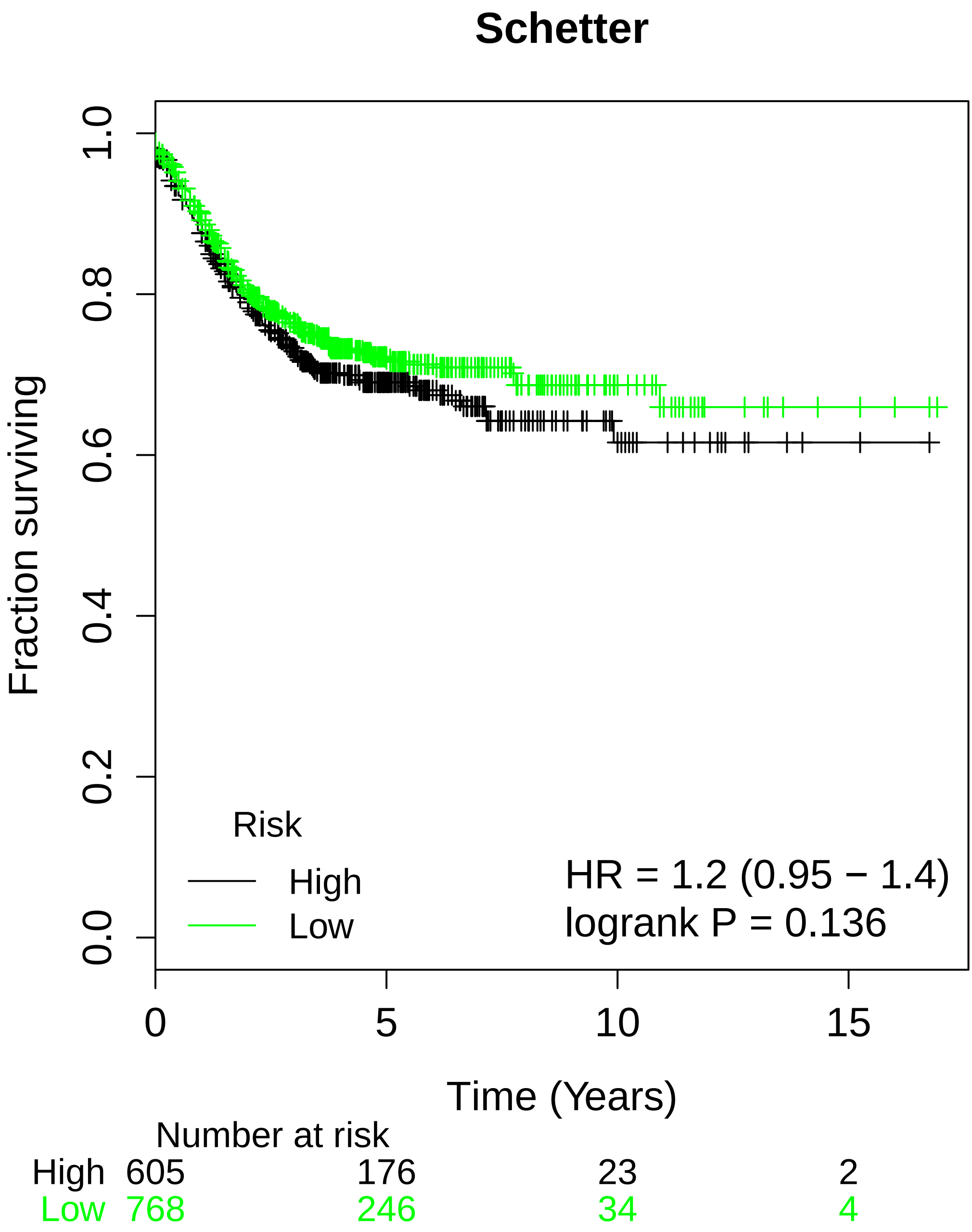

Schetter
Fraction surviving
1.0
0.8
0.6
0.4
0.2
0.0
0
5
10
15
Time (Years)
Risk
High
Low
HR = 1.2 (0.95 − 1.4)
logrank P = 0.136
Number at risk
High 605 176 23 2
Low 768 246 34 4

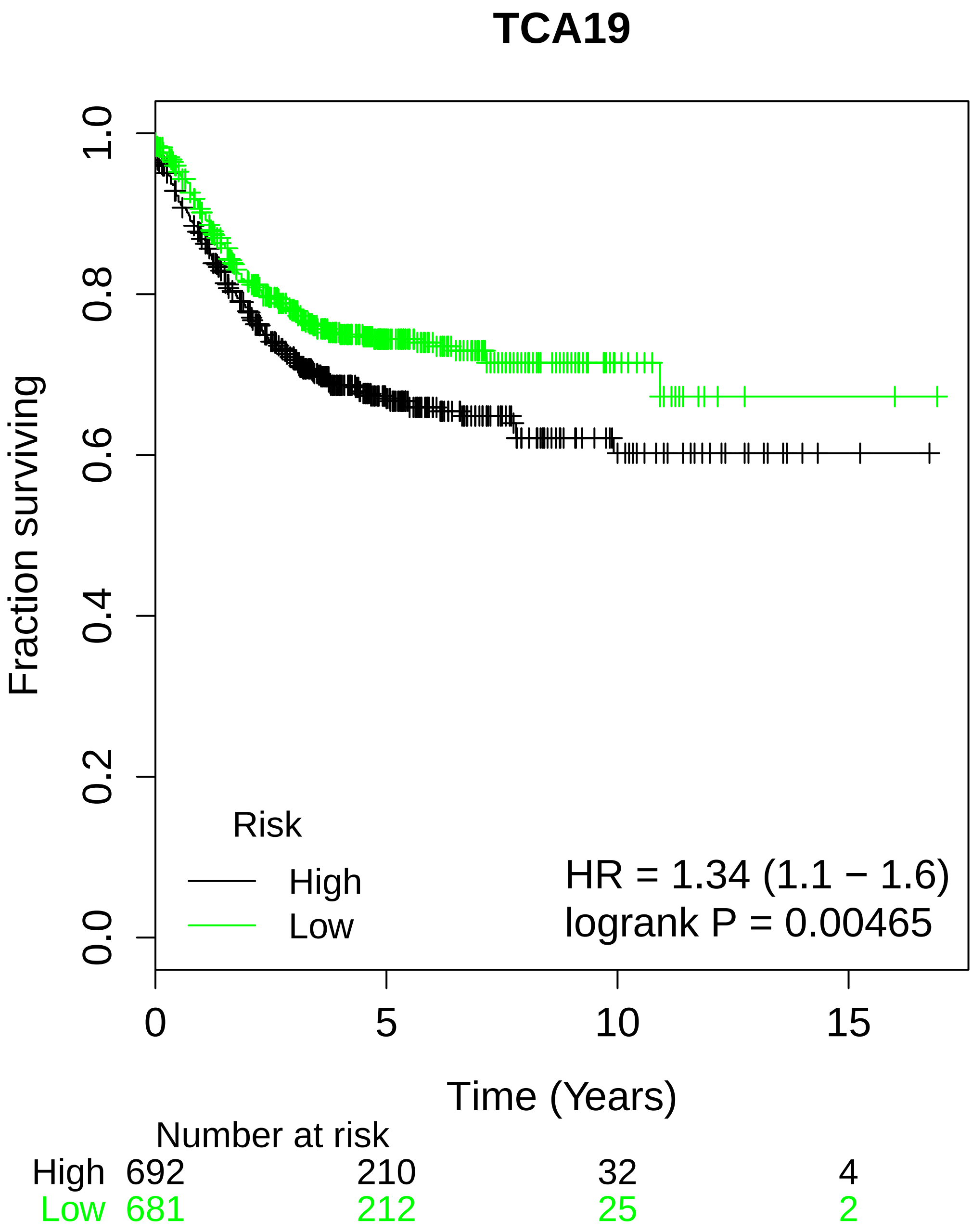
TCA19
Fraction surviving
1.0
0.8
0.6
0.4
0.2
0.0
Risk
High
Low
HR = 1.34 (1.1 − 1.6)
logrank P = 0.00465
0
5
10
15
Time (Years)
Number at risk
High 692 210 32 4
Low 681 212 25 2

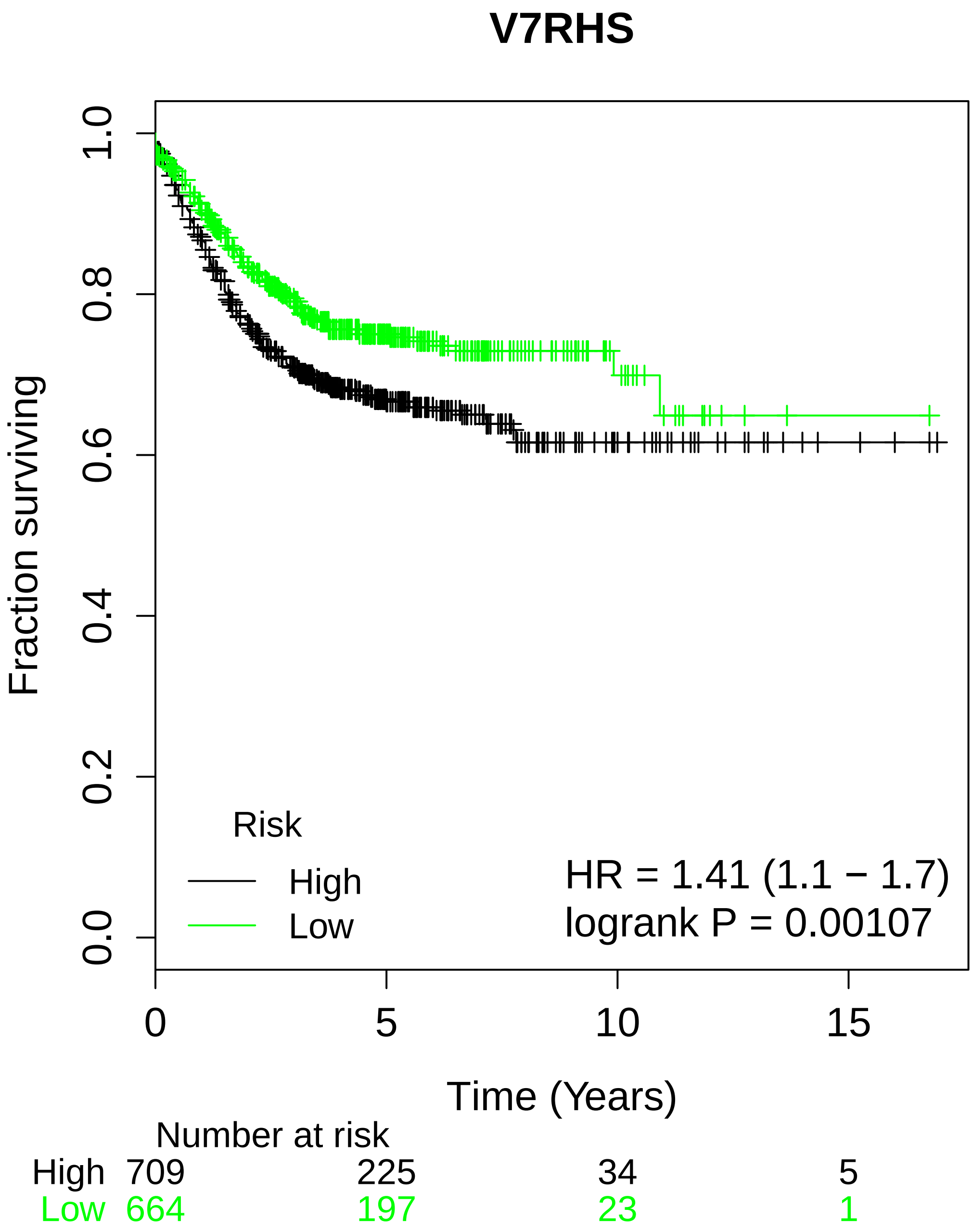
V7RHS
Fraction surviving
1.0
0.8
0.6
0.4
0.2
0.0
Risk
High
Low
HR = 1.41 (1.1 − 1.7)
logrank P = 0.00107
0
5
10
15
Time (Years)
Number at risk
High 709 225 34 5
Low 664 197 23 1

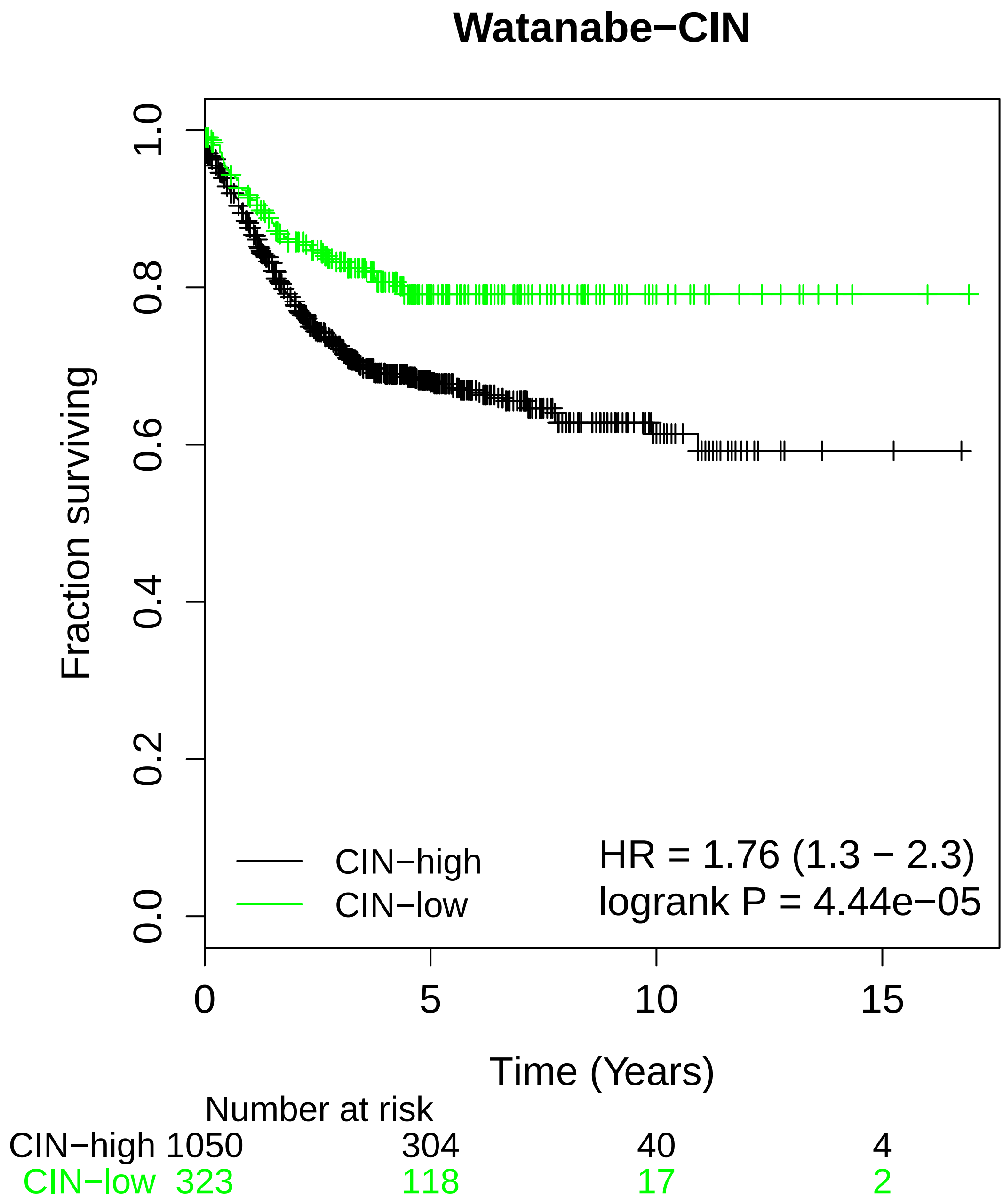
Watanabe−CIN
Fraction surviving
0.0
0.2
0.4
0.6
0.8
1.0
0
5
10
15
Time (Years)
CIN−high
CIN−low
HR = 1.76 (1.3 − 2.3)
logrank P = 4.44e−05
Number at risk
CIN−high 1050 304 40 4
CIN−low 323 118 17 2

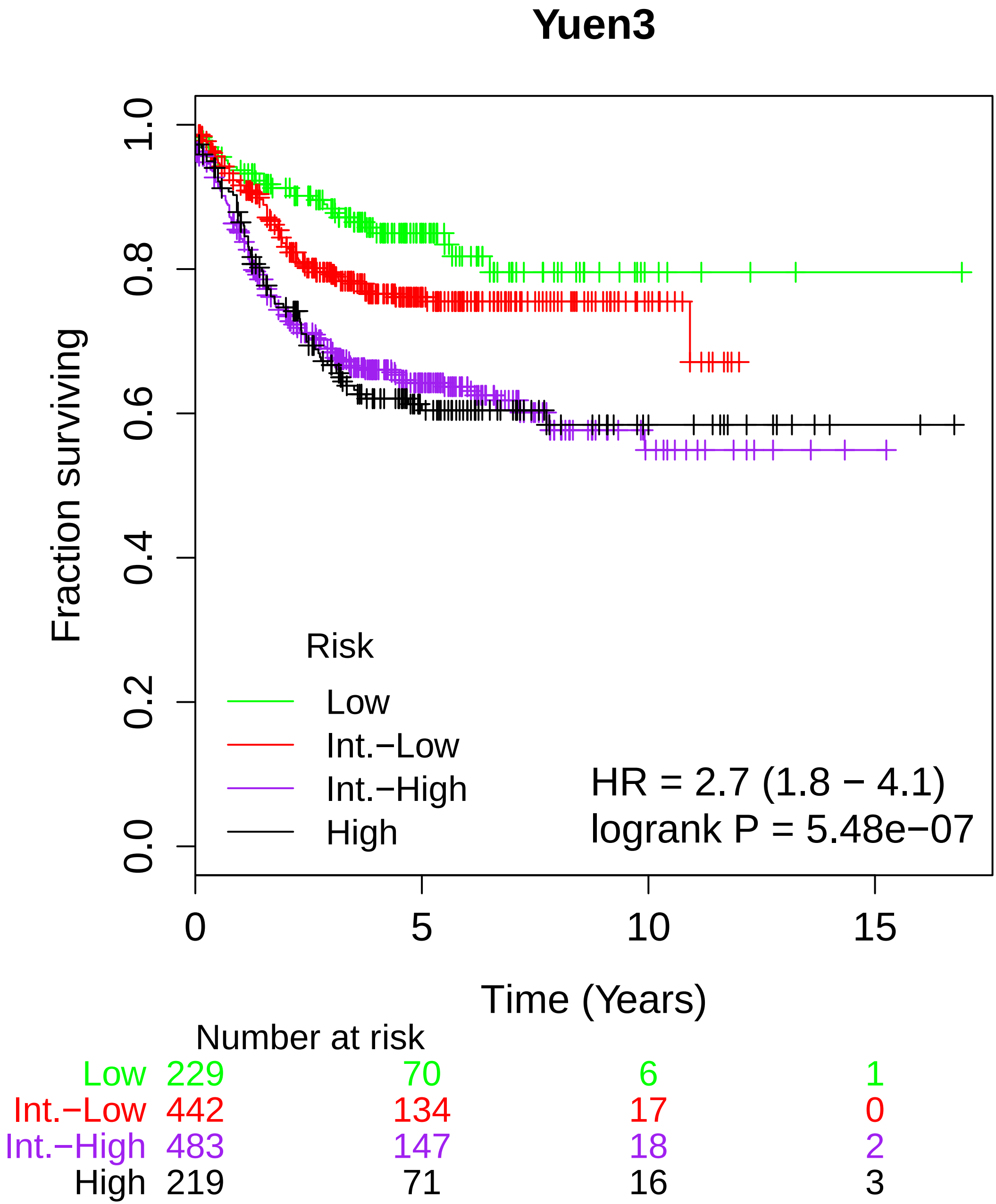
Yuen3
Fraction surviving
1.0
0.8
0.6
0.4
0.2
0.0
Risk
Low
Int.−Low
Int.−High
High
HR = 2.7 (1.8 − 4.1)
logrank P = 5.48e−07
0
5
10
15
Time (Years)
Number at risk
Low 229 70 6 1
Int.−Low 442 134 17 0
Int.−High 483 147 18 2
High 219 71 16 3
